# Virological response and resistance among HIV-infected children receiving long-term antiretroviral therapy without virological monitoring in Uganda and Zimbabwe: Observational analyses within the randomised ARROW trial

**DOI:** 10.1371/journal.pmed.1002432

**Published:** 2017-11-14

**Authors:** Alexander J. Szubert, Andrew J. Prendergast, Moira J. Spyer, Victor Musiime, Philippa Musoke, Mutsa Bwakura-Dangarembizi, Patricia Nahirya-Ntege, Margaret J. Thomason, Emmanuel Ndashimye, Immaculate Nkanya, Oscar Senfuma, Boniface Mudenge, Nigel Klein, Diana M. Gibb, A. Sarah Walker, the ARROW Trial Team

**Affiliations:** 1 MRC Clinical Trials Unit at University College London, London, United Kingdom; 2 Queen Mary University of London, London, United Kingdom; 3 Joint Clinical Research Centre, Kampala, Uganda; 4 Makerere University College of Health Sciences, Kampala, Uganda; 5 Paediatric Infectious Diseases Clinic/Baylor-Uganda, Kampala, Uganda; 6 University of Zimbabwe, Harare, Zimbabwe; 7 MRC/UVRI Uganda Research Unit on AIDS, Entebbe, Uganda; 8 Flow Cytometry Laboratory, Harare, Zimbabwe; 9 University College London Great Ormond Street Institute of Child Health, London, United Kingdom; University of Southampton, UNITED KINGDOM

## Abstract

**Background:**

Although WHO recommends viral load (VL) monitoring for those on antiretroviral therapy (ART), availability in low-income countries remains limited. We investigated long-term VL and resistance in HIV-infected children managed without real-time VL monitoring.

**Methods and findings:**

In the ARROW factorial trial, 1,206 children initiating ART in Uganda and Zimbabwe between 15 March 2007 and 18 November 2008, aged a median 6 years old, with median CD4% of 12%, were randomised to monitoring with or without 12-weekly CD4 counts and to receive 2 nucleoside reverse transcriptase inhibitors (2NRTI, mainly abacavir+lamivudine) with a non-nucleoside reverse transcriptase inhibitor (NNRTI) or 3 NRTIs as long-term ART. All children had VL assayed retrospectively after a median of 4 years on ART; those with >1,000 copies/ml were genotyped. Three hundred and sixteen children had VL and genotypes assayed longitudinally (at least every 24 weeks). Overall, 67 (6%) switched to second-line ART and 54 (4%) died. In children randomised to WHO-recommended 2NRTI+NNRTI long-term ART, 308/378 (81%) monitored with CD4 counts versus 297/375 (79%) without had VL <1,000 copies/ml at 4 years (difference = +2.3% [95% CI −3.4% to +8.0%]; *P* = 0.43), with no evidence of differences in intermediate/high-level resistance to 11 drugs. Among children with longitudinal VLs, only 5% of child-time post–week 24 was spent with persistent low-level viraemia (80–5,000 copies/ml) and 10% with VL rebound ≥5,000 copies/ml. No child resuppressed <80 copies/ml after confirmed VL rebound ≥5,000 copies/ml. A median of 1.0 (IQR 0.0,1.5) additional NRTI mutation accumulated over 2 years’ rebound. Nineteen out of 48 (40%) VLs 1,000–5,000 copies/ml were immediately followed by resuppression <1,000 copies/ml, but only 17/155 (11%) VLs ≥5,000 copies/ml resuppressed (*P* < 0.0001). Main study limitations are that analyses were exploratory and treatment initiation used 2006 criteria, without pre-ART genotypes.

**Conclusions:**

In this study, children receiving first-line ART in sub-Saharan Africa without real-time VL monitoring had good virological and resistance outcomes over 4 years, regardless of CD4 monitoring strategy. Many children with detectable low-level viraemia spontaneously resuppressed, highlighting the importance of confirming virological failure before switching to second-line therapy. Children experiencing rebound ≥5,000 copies/ml were much less likely to resuppress, but NRTI resistance increased only slowly. These results are relevant to the increasing numbers of HIV-infected children receiving first-line ART in sub-Saharan Africa with limited access to virological monitoring.

**Trial registration:**

ISRCTN Registry, ISRCTN24791884

## Abstract 2

In an analysis based on the ARROW trial, Alex Szubert and colleagues report on virological and drug resistance outcomes in children with HIV infection.

## Author summary

**Why was this study done?:**

- Although WHO guidelines recommend that HIV-infected children be monitored using HIV viral load tests when they are receiving treatment, availability remains limited.
- Most HIV-infected children on treatment therefore get either no or infrequent viral load tests; they may get CD4 count measurements and regular clinical assessments.
- We wanted to find out what implications this might have for children’s long-term response to treatment, in terms of controlling the virus and development of resistance to antiretroviral drugs.

**What did the researchers do and find?:**

- We measured viral load and drug resistance using stored samples from a large randomised controlled trial of 1,206 HIV-infected children starting treatment in Uganda and Zimbabwe who were randomised to monitoring with or without CD4 counts, as well as to 2 different first-line antiretroviral drug regimens. Two-thirds received the treatment regimen that WHO now recommends long-term (2 nucleoside reverse transcriptase inhibitors and 1 non-nucleoside reverse transcriptase inhibitor).
- After an average of 4 years on treatment, we found no evidence that CD4 monitoring strategy affected viral suppression or resistance. Overall, 74% of the 753 children randomised to current WHO-recommended regimens were fully suppressed.
- We also investigated how viral load changed and resistance developed over time in a subset of children taking current WHO-recommended regimens. After the first 24 weeks on treatment, only 5% and 10% of child-time was spent with persistent lower-level viral load (80–5,000 copies/ml) or rebound to ≥5,000 copies/ml, respectively. Even among children with high viral load, intermediate- to high-level drug resistance to key second-line drugs was found only in a minority, although children with virological rebound had slight increases in resistance over 2 years.

**What do these findings mean?:**

- In this study, children on currently recommended WHO regimens did very well, whether or not they were monitored using CD4 counts. Therefore, getting treatment to more children remains the most important activity, even if viral load and/or CD4 monitoring is not available.
- Considerable variability in viral loads and substantial resuppression, particularly in children with single measurements 1,000–5,000 copies/ml, means that care needs to be taken not to switch too early to second-line drugs in order to maximise the time that children can successfully spend on first-line treatment.
- Among children experiencing confirmed viral rebound ≥5,000 copies/ml, resistance increased only slightly over 2 years, providing reassurance that annual VL monitoring, as recommended by WHO, is adequate in resource-limited settings.

## Introduction

The Joint United Nations Programme on HIV/AIDS (UNAIDS) has set an ambitious target for 2020: that 90% of people living with HIV know their status, 90% of those diagnosed receive antiretroviral therapy (ART), and 90% receiving ART have viral load (VL) suppression [1]. There are an estimated 1.8 million HIV-infected children worldwide, >80% of whom live in sub-Saharan Africa. Treating children is challenging, given their more limited access to antiretroviral drugs compared with adults; in 2015, only 49% of HIV-infected children globally were estimated to be receiving ART [2]. Current WHO guidelines recommend VL monitoring 6 and 12 months after initiating ART, and every 12 months thereafter [3]. A threshold of >1,000 copies/ml is used to define virological failure, which, if confirmed and adherence has been addressed, should be followed by the switch to second-line ART. Current guidelines also recommend stopping routine CD4 monitoring, provided patients are clinically stable and virologically suppressed. However, despite this push to increase VL monitoring and reduce reliance on immunological and clinical failure criteria, the availability of VL monitoring remains limited in low-income settings (estimated at 25% of people living with HIV in 2014 [4]), and VL is less frequently measured in low-income settings than in well-resourced ones (typically annually versus 3-monthly). Therefore, it is important to understand virological outcomes among children who are not managed using real-time VL monitoring, as this reflects the reality in most ART programmes in resource-limited settings.

Although management without VL monitoring has enabled ART rollout, children with virological failure risk developing resistance, which may reduce efficacy of second-line therapy. In a systematic review of 30 studies from low-income settings in 2011 [5], 80% and 88% of children failing first-line ART had 1 or more International AIDS Society (IAS)-USA major nucleoside reverse transcriptase inhibitor (NRTI) and non-nucleoside reverse transcriptase inhibitor (NNRTI) resistance-associated mutations, respectively. In 2012, WHO reported that 60% and 67% of adults and children in sub-Saharan Africa failing first-line ART at 12 months had NRTI and NNRTI resistance, respectively [6]. The only large randomised trial to date of second-line ART in African adults found that those receiving NRTI-based second-line regimens had equivalent virological suppression to those receiving regimens based on 2 new drug classes [7], suggesting that NRTI cross-resistance may have less relevance than feared. However, longitudinal VL and resistance data are rare in children starting first-line ART in resource-limited settings.

We therefore investigated virological outcomes and resistance in the ARROW randomised trial (ISRCTN24791884), which compared first-line ART regimens and CD4 monitoring strategies in children and adolescents in Uganda and Zimbabwe [8]. Overall, ARROW showed that CD4 monitoring, compared with clinical monitoring alone, provided some clinical benefit after the first year on ART, but 5-year survival was very high overall (96%), supporting prioritisation of ART rollout over access to laboratory monitoring. There was no evidence of differences between 3NRTI and 2NRTI+NNRTI long-term maintenance regimens in terms of clinical outcomes or CD4, but medium-term virological suppression was poorer with 3NRTI, supporting 2NRTI+NNRTI becoming the WHO-recommended first-line regimen for children as of 2013.

The ARROW trial provided a unique opportunity to evaluate long-term virological outcomes in stored samples from a large cohort of well-characterised children with high retention in whom VL monitoring was not undertaken in real-time. In the present study, we had 2 main objectives. The first was to directly compare children randomised to monitoring with and without CD4 counts in terms of long-term VL suppression and resistance. The second was to characterise the patterns of long-term virological control and resistance in children receiving first-line ART in sub-Saharan Africa without frequent, regular real-time VL monitoring (reflecting the reality in most ART programmes) in an observational analysis without regard to the trial randomisations. We focussed on children receiving currently WHO-recommended 2NRTI+NNRTI maintenance ART regimens.

## Materials and methods

The ARROW trial (ISRCTN24791884) recruited 1,206 previously untreated HIV-infected children and adolescents (aged 3 months to 17 years) eligible for ART using WHO 2006 criteria [8], between 15 March 2007 and 18 November 2008. Children were recruited from 3 centres in Uganda and 1 in Zimbabwe. Caregivers gave written informed consent for all children to enrol; if older children (8–17 years) were aware of their HIV status, they gave additional assent or consent following national guidelines. The ARROW trial and this current study were approved by Research Ethics Committees and Institutional Review Boards for Uganda (Joint Clinical Research Centre and Baylor College of Medicine and the Uganda National Council for Science and Technology [UNCST]), for Zimbabwe (Medical Research Council of Zimbabwe [MRCZ/A/1321]), and for the United Kingdom (University College London [0701/001]).

Children were randomised 1:1 to clinically driven monitoring (CDM; CD4 counts measured 12-weekly in real time but not returned to physicians) or laboratory plus clinical monitoring (LCM; CD4 counts measured 12-weekly) (CONSORT diagram in Figure 1 of [8]). Children were also randomised 1:1:1 in a factorial design to 3 first-line regimens: open-label lamivudine+abacavir+NNRTI continuously (Arm-A); induction-maintenance with 4-drug lamivudine+abacavir+NNRTI+zidovudine for 36 weeks, followed by lamivudine+abacavir+NNRTI (Arm-B); or lamivudine+abacavir+zidovudine (Arm-C; 3NRTI including long-term zidovudine). The NNRTI (nevirapine/efavirenz) was chosen by clinicians according to local availability (varying by country) and child’s age. After ≥36 weeks, eligible children taking lamivudine+abacavir twice daily were randomised to continue twice daily or move to once daily [9]. Following WHO guidelines at the time [10], switch to second-line ritonavir-boosted protease-inhibitor-containing ART was discouraged in the first year of therapy. Thereafter, switch was based on clinical criteria in all participants (new/recurrent WHO stage 4 event [9]; or WHO stage 3 event(s) at clinician discretion, particularly if recurrent or persistent) or on additional immunological criteria for LCM (confirmed on-ART CD4 <15% if aged 1–2 years, <10% if aged 3–4 years, <100 cells/μL if aged ≥5 years). VLs were not measured in real time nor used for any management. Children were followed until death or planned trial closure on 16 March 2012.

### Study design

**Fig 1:**
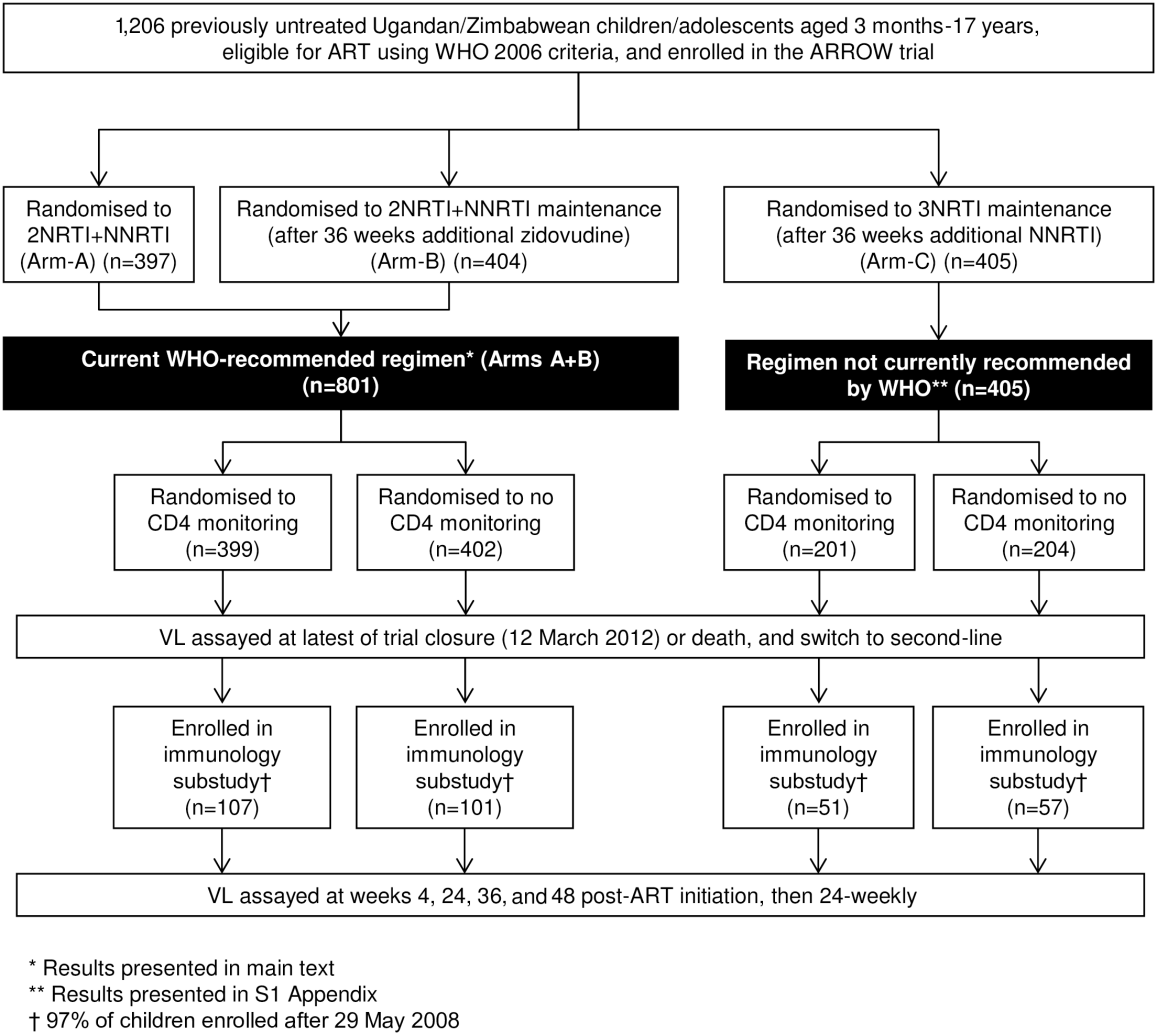
Flow diagram of children included in virological analyses. * Results presented in main text. ** Results presented in S1 Appendix. † 97% of children enrolled after 29 May 2008. Abbreviations: ART, antiretroviral therapy; NNRTI, non-nucleoside reverse transcriptase inhibitor; NRTI, nucleoside reverse transcriptase inhibitor; VL, viral load.

In the current study, we evaluated VL and resistance in 2 separate analyses (Fig 1). First, we undertook a cross-sectional analysis (“cross-sectional study”) of VL assayed in all children on cryopreserved plasma taken up to 6 months prior to the latest of trial closure (16 March 2012) or death and also at switch to second-line; samples with VL >1,000 copies/ml were genotyped. Second, we undertook a longitudinal study in a subgroup of 316 children randomised from 29 May 2008 (“longitudinal cohort”). In these children, retrospective VL was assayed longitudinally at weeks 4, 24, 36, and 48 post-ART initiation, then every 24 weeks, with a small number of additional VLs included from a previous analysis [10] (see S1 Appendix for assay completeness over time). Genotyping was undertaken on samples with VL >1,000 copies/ml at week 48 or 144. For both studies, we present results for children taking WHO-recommended 2NRTI+NNRTI regimens in the main text and results for all children, including those randomised to 3NRTI, in S1 Appendix. The sample size for the first study was determined by the size of the trial [8] and for the second study by the size of the longitudinal cohort that aimed to include all children randomised from 29 May 2008 when seed funding was obtained.

### Laboratory methods

VLs were assayed blind to randomised group using Abbott m2000sp/rt or Roche COBAS Amplicor Monitor ultrasensitive tests, v1.5, with lower limit of detection of 80 copies/ml because many samples were diluted 1:2 due to low volumes. Genotyping for reverse transcriptase was done blind to randomised group using an in-house assay at a WHO-designated laboratory (Joint Clinical Research Centre, Kampala, Uganda) using the ABI 3730xl sequencer. Major NRTI and NNRTI mutations were defined using IAS-USA 2013 [11], and drug susceptibility was predicted using the Stanford algorithm version 7 [12].

### Virological definitions

In the longitudinal cohort, we predefined complete VL response as <10,000 copies/ml or >1 log_10_ drop at week 4, <5,000 copies/ml at week 24, declining or <400 copies/ml at week 36, and subsequently <80 copies/ml at all measurements. VL measurements ≥80 copies/ml were classified as a blip if the child had a previous VL <80 copies/ml and either subsequently resuppressed <80 copies/ml (1 or 2 VL ≥80 copies/ml allowed; *n* = 150) or their last measurement was a single value ≥80 copies/ml (*n* = 31; S1 Appendix). Those not resuppressing <80 copies/ml but remaining <5,000 copies/ml were defined as persistent low-level viral load (pLLVL), whereas rebound was defined as confirmed VL ≥5,000 copies/ml (following WHO 2010 guidelines [13]). Each individual VL measurement on first-line ART was classified (S1 Table); in order to record the greatest degree of viral replication observed to have occurred, children could move from response to blip, pLLVL, and rebound states but not backwards through these states; i.e., if a child had ever experienced a VL blip, they were subsequently classified as having previously ‘blipped’.

### Statistical analysis

Other than the overall comparison of VL suppression and resistance by monitoring randomisation at trial closure (protocol secondary endpoints), all analyses were exploratory and not part of the prespecified protocol (S1 Protocol) or analysis plan. Categorical factors were compared between randomised and other groups using chi-squared tests, unless otherwise stated, based on complete cases. Log_10_VL trajectories in pLLVL and rebound from the first measurement in the state were estimated using multilevel models with child-level random intercepts and slopes (unstructured correlation for rebound; independent for pLLVL, as correlation parameter was not estimable). Resistance was summarised at the first VL in rebound, then at the last available genotype tested subsequently, using signed-rank tests to compare repeated genotypes within each child. As VLs may be performed irregularly in clinical practice, in the longitudinal cohort we also estimated the probability that a single or confirmed VL above various thresholds (80, 200, 400, 1,000, and 5,000 copies/ml) post–week 24 would have been followed by the next VL 5–30 weeks later returning below that threshold.

All analyses were performed using Stata 14.2 (StataCorp). All *P* values are 2-sided.

## Results

One thousand two hundred and six children initiated ART in ARROW, aged median 6.0 years (IQR 2.4,9.3) with median CD4% 12% (7%,17%) (Table 1). Over a median 4 years follow-up, 54 children (4%) died [9], 39/54 (72%) before week 48. Sixty-seven children (6%) switched to ritonavir-boosted protease-inhibitor-containing regimens (see S1 Appendix for details of VL in children who died or switched).

**Table 1:** Characteristics at ART initiation of all children in the trial; children randomised to 2NRTI+NNRTI followed in the longitudinal cohort; and those subsequently experiencing VL blips, pLLVL, and/or rebound.

Factor (at ART initiation)	All ARROW children *N* = 1,206	2NRTI+NNRTI longitudinal cohort *N* = 204* Median (IQR) or *n* (%)	2NRTI+NNRTI ever experienced VL blips *N* = 93 Median (IQR) or *n* (%)	2NRTI+NNRTI ever experienced pLLVL *N* = 20 Median (IQR) or *n* (%)	2NRTI+NNRTI ever experienced rebound *N* = 28 Median (IQR) or *n* (%)
Male	596 (49.4%)	97 (47.5%)	46 (49.5%)	8 (40.0%)	13 (46.4%)
Centre					
A	400 (33.2%)	70 (34.3%)	46 (49.5%)	1 (5.0%)	4 (14.3%)
B	188 (15.6%)	32 (15.7%)	9 (9.7%)	2 (10.0%)	8 (28.6%)
C	318 (26.4%)	53 (26.0%)	18 (19.4%)	11 (55.0%)	6 (21.4%)
D	300 (24.9%)	49 (24.0%)	20 (21.5%)	6 (30.0%)	10 (35.7%)
Age (years)	6.0 (2.4, 9.3)	5.9 (2.4, 9.4)	5.4 (2.4, 8.1)	5.2 (2.4, 10.4)	6.4 (1.5, 12.4)
Weight-for-age**	−2.2 (−3.3, −1.3)	−2.1 (−3.3, −1.3)	−2.0 (−3.0, −1.3)	−2.4 (−3.7, −1.4)	−2.1 (−3.3, −1.3)
Height-for-age**	−2.4 (−3.4, −1.5)	−2.6 (−3.4, −1.7)	−2.8 (−3.4, −1.7)	−2.5 (−3.5, −1.4)	−2.1 (−3.2, −1.3)
VL (copies/ml)	268,800 (89,200, 748,700)^0^	212,700 (61,400, 608,800)^1^	273,400 (116,400, 741,800)^1^	253,800 (105,800, 721,800)	316,000 (103,100, 1,149,300)
CD4%	12 (7, 17)	13 (7, 19)	13 (8, 18)	13 (6, 17)	7 (3, 16)
WHO stage					
1 or 2	354 (29.4%)	69 (33.8%)	30 (32.3%)	9 (45.0%)	10 (35.7%)
3 or 4	852 (70.6%)	135 (66.2%)	63 (67.7%)	11 (55.0%)	18 (64.3%)
Monitoring					
CD4 monitoring	600 (49.8%)	104 (51.0%)	47 (50.5%)	13 (65.0%)	14 (50.0%)
No CD4 monitoring	606 (50.2%)	100 (49.0%)	46 (49.5%)	7 (35.0%)	14 (50.0%)
Initial NNRTI					
Nevirapine	758 (62.9%)	110 (53.9%)	53 (57.0%)	12 (60.0%)	13 (46.4%)
Efavirenz	448 (37.1%)	94 (46.1%)	40 (43.0%)	8 (40.0%)	15 (53.6%)

* Excluding 2 children who died before 4 weeks (with baseline VL only) and 2 non-responders (never achieved VL <9,500 c/ml).

** Height-for-age calculated using WHO reference [14]; as weight-for-age only covers children aged <121 months, this was calculated using the UK reference that covers the full age range of ARROW children [15] (Spearman correlation between UK and WHO references = 0.99 in *n* = 971 children aged <121 months).

^0^ Assayed for 867/1,206 (72%) children.

^1^ Missing for 1 child.

Note: Children experiencing more than 1 of VL blips, pLLVL, and rebound included in all relevant columns.

Abbreviations: ART, antiretroviral therapy; NNRTI, non-nucleoside reverse transcriptase inhibitor; NRTI, nucleoside reverse transcriptase inhibitor; VL, viral load.

### Long-term virological response (cross-sectional study)

One thousand one hundred and thirty-two children (94%) were alive and in follow-up at trial closure with VL measurements available for 1,127/1,132 (99.6%) after median 3.9 years on ART (IQR 3.7,4.4). As expected, long-term virological suppression was significantly greater on the WHO-recommended 2NRTI+NNRTI maintenance regimen than 3NRTI maintenance (S1 Fig). All subsequent analyses therefore focussed on children randomised to the WHO-recommended 2NRTI+NNRTI maintenance regimen (see S1 Appendix for analyses in 3NRTI).

**Fig 2:**
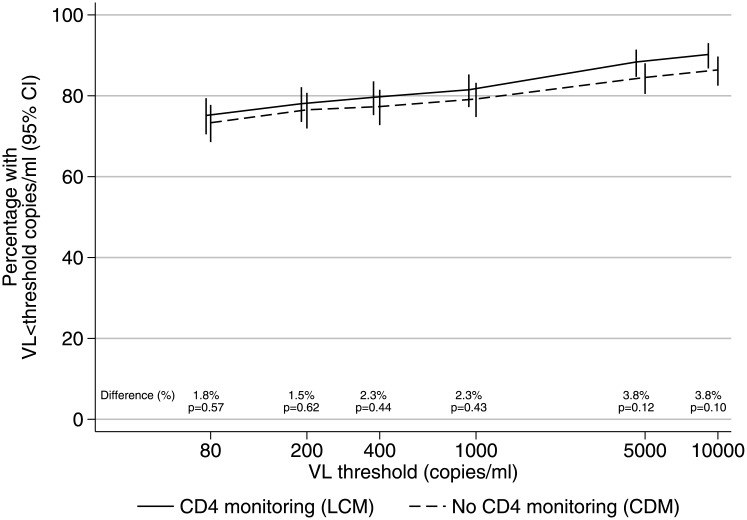
VL suppression in 2NRTI+NNRTI maintenance regimen after median 4 years on ART. Note: 95% CI for risk differences were <80 copies/ml [−4.4% to +8.0%], <200 copies/ml [−4.5% to +7.5%], <400 copies/ml [−3.6% to +8.2%], <1,000 copies/ml [−3.4% to +8.0%], <5,000 copies/ml [−1.1% to +8.7%], and <10,000 copies/ml [−0.8% to +8.4%]. Abbreviations: ART, antiretroviral therapy; CDM, clinically driven monitoring; LCM, laboratory plus clinical monitoring; NNRTI, non-nucleoside reverse transcriptase inhibitor; NRTI, nucleoside reverse transcriptase inhibitor; VL, viral load.

There was no difference in long-term virological suppression between those monitored with versus without CD4 counts (chi-squared *P* > 0.1 across thresholds shown in Fig 2). Among children receiving the WHO-recommended 2NRTI+NNRTI regimen, 284/378 (75%) monitored with CD4s versus 275/375 (73%) monitored without CD4s had VL <80 copies/ml after 4 years on ART (difference = +1.8% [95% CI −4.4% to +8.0%]; chi-squared *P* = 0.57), and 308 (81%) versus 297 (79%), respectively, had VL <1,000 copies/ml (difference = +2.3% [95% CI −3.4% to +8.0%]; chi-squared *P* = 0.43).

**Fig 3:**
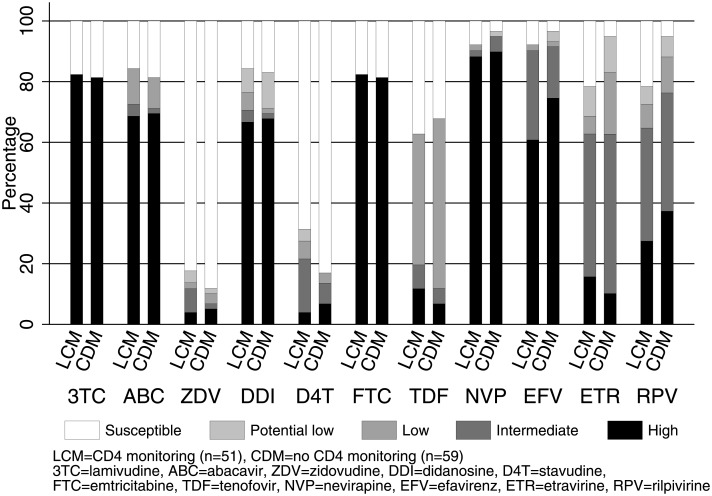
Predicted NRTI and NNRTI susceptibility in 2NRTI+NNRTI with VL >1,000 copies/ml after median 4 years on ART (*n* = 110). Note: risk differences and 95% CI between CD4 monitoring versus no CD4 monitoring with intermediate/high-level resistance were 3TC 1.0% [−13.4% to +15.4%], ABC 1.4% [−15.5% to +18.2%], ZDV 5.0% [−5.9% to +15.9%], DDI 1.1% [−16.1% to +18.3%], D4T 8.0% [−6.3% to +22.3%], FTC 1.0% [−13.4% to +15.4%], TDF 7.7% [−5.9% to +21.4%], NVP 4.7% [−14.6% to +5.2%], EFV 1.3% [−12.2% to +9.5%], ETR 0.0% [−18.1% to +18.2%], and RPV 11.6% [−28.6% to +5.5%]. Abbreviations: ART, antiretroviral therapy; CDM, clinically driven monitoring; LCM, laboratory plus clinical monitoring; NNRTI, non-nucleoside reverse transcriptase inhibitor; NRTI, nucleoside reverse transcriptase inhibitor; VL, viral load.

Of 110 children with VL >1,000 copies/ml and genotype on WHO-recommended 2NRTI+NNRTI (the vast majority on lamivudine+abacavir), only 17 (15%) had intermediate/high-level resistance to tenofovir and 10 (9%) to zidovudine, the key NRTI backbone drugs used in second-line treatment (Fig 3). There was no evidence of differences between CD4 monitoring groups in intermediate/high-level resistance to NRTIs or NNRTIs (exact *P* > 0.2). Prevalence of IAS-USA NRTI or NNRTI mutations was also similar (S2 Fig). 42 (82%) monitored with CD4s versus 48 (81%) without had the M184V mutation conferring resistance to lamivudine (difference = +1.0% [95% CI −13.4% to +15.4%]). Only 8 children (7%) (5 CD4-monitoring, 3 no CD4-monitoring) had the K65R NRTI mutation.

### VL control over time on first-line WHO-recommended ART without real-time VL monitoring (longitudinal cohort)

We next evaluated virological responses over time in the 208 of the 316 longitudinal cohort children randomised to 2NRTI+NNRTI maintenance. At ART initiation, these children were broadly similar to those in the whole trial [9] (Table 1). Two children (1%) died before week 4 and had only baseline VL. Of the remaining 206 children, 204 (99%) achieved an initial VL response and were included in subsequent analyses. Only 2 children (1%) switched to second-line and 2 (1%) died after week 4 (1 VL responder, 1 in rebound).

**Fig 4:**
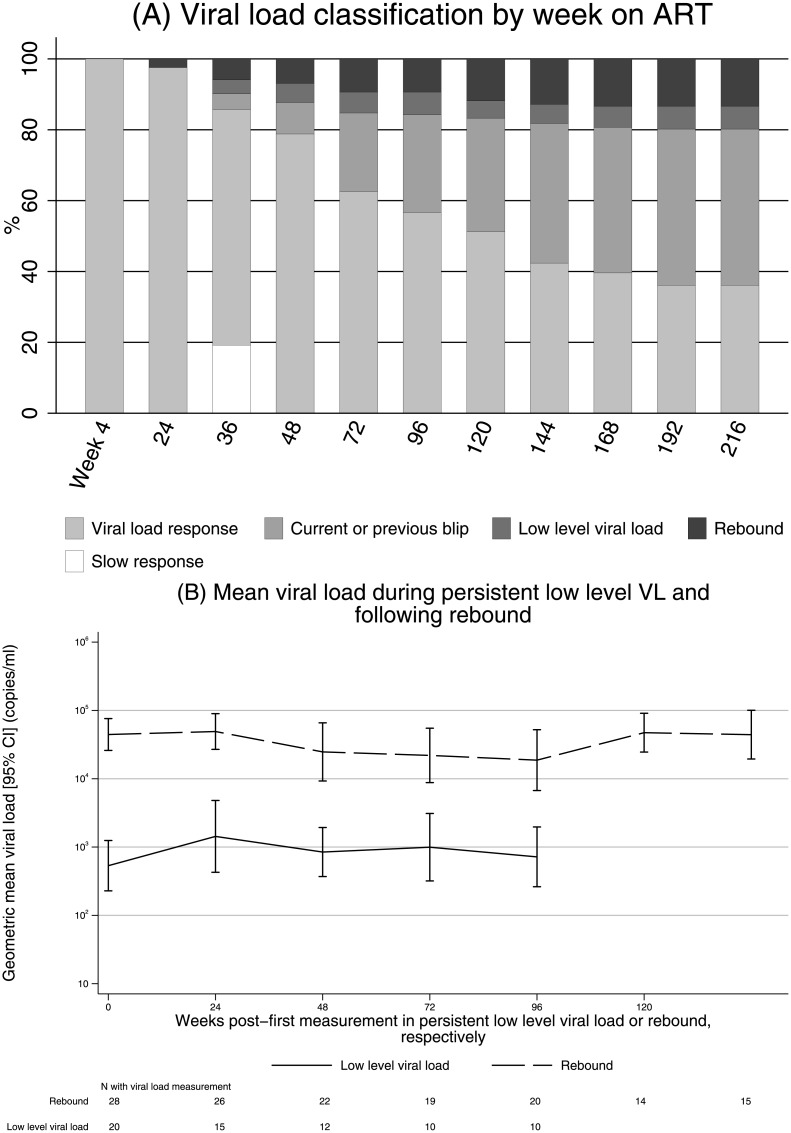
VL response in 204 children randomised to 2NRTI+NNRTI initially responding to ART. (a) VL classification by week on ART. (b) Mean VL during pLLVL and rebound. Note: (a) carrying forwards current state where VL was missing; carrying backwards, “VL response”, where week 4 VL was missing (*n* = 16). Abbreviations: ART, antiretroviral therapy; NNRTI, non-nucleoside reverse transcriptase inhibitor; NRTI, nucleoside reverse transcriptase inhibitor; pLLVL, persistent low-level viral load; VL, viral load.

VL measurements were classified as response, current or previous blip, pLLVL (80–5,000 copies/ml), or rebound (≥5,000 copies/ml). The differing levels of VL control over time are shown in Fig 4a. Interestingly 39 children (19%) were slow VL responders, i.e., still ≥80 copies/ml at week 36 but <80 copies/ml by week 48. After 72 weeks on ART, the percentage of children with either pLLVL or VL rebound stabilised and then remained constant; however, an increasing percentage had experienced at least 1 viral blip. In total, 93 children (46%) experienced 132 single (*n* = 118, 89%) or double (*n* = 14, 11%) blips ≥80 copies/ml, but the median (IQR) VL during these blips was only 200 (110–560) copies/ml, and children who had blipped did not progress faster to pLLVL or rebound than children remaining with complete VL response (rate 1.2 versus 4.7 per 100 child-years, respectively; S3 Fig). Overall, post–week 24, these children randomised to WHO-recommended 2NRTI+NNRTI spent 60% of child-time as complete VL responders and 85% as either complete VL responders or having had blips; 5% of time was spent with pLLVL and 10% with rebound >5,000 copies/ml. As for the cross-sectional cohort, there was no evidence of any difference in VL response between those monitored with or without CD4 counts throughout (exact *P* > 0.1, S3 Table).

Overall, 20 children (10%) experienced pLLVL. Geometric mean VL at the start of pLLVL was 780 copies/ml (95% CI 420–1,460) and did not increase over the subsequent (Kaplan-Meier) median >156 weeks (IQR 37,>156) spent with pLLVL through the end of follow-up (change −0.02 log_10_ per year [95% CI −0.20 to +0.16], *P* = 0.81; Fig 4b). In addition, some children with pLLVL resuppressed, albeit transiently; 21/105 (20%) measurements in pLLVL were <80 copies/ml.

Twenty-eight children (14%) experienced rebound to ≥5,000 copies/ml, most commonly following pLLVL (rate of progression from pLLVL to rebound 21.3/100 child-years, S3 Fig). Geometric mean VL at rebound was 41,910 copies/ml (95% CI 27,100–64,810); VL did not increase over the median of 95 weeks (IQR 25,137) spent with rebound (change −0.11 log_10_ per year [95% CI −0.27 to +0.05], *P* = 0.18; Fig 4b). In contrast to pLLVL, no measurements after confirmed rebound ≥5,000 copies/ml were <80 copies/ml.

### Development of resistance on first-line 2NRTI+NNRTI without routine VL monitoring

**Fig 5:**
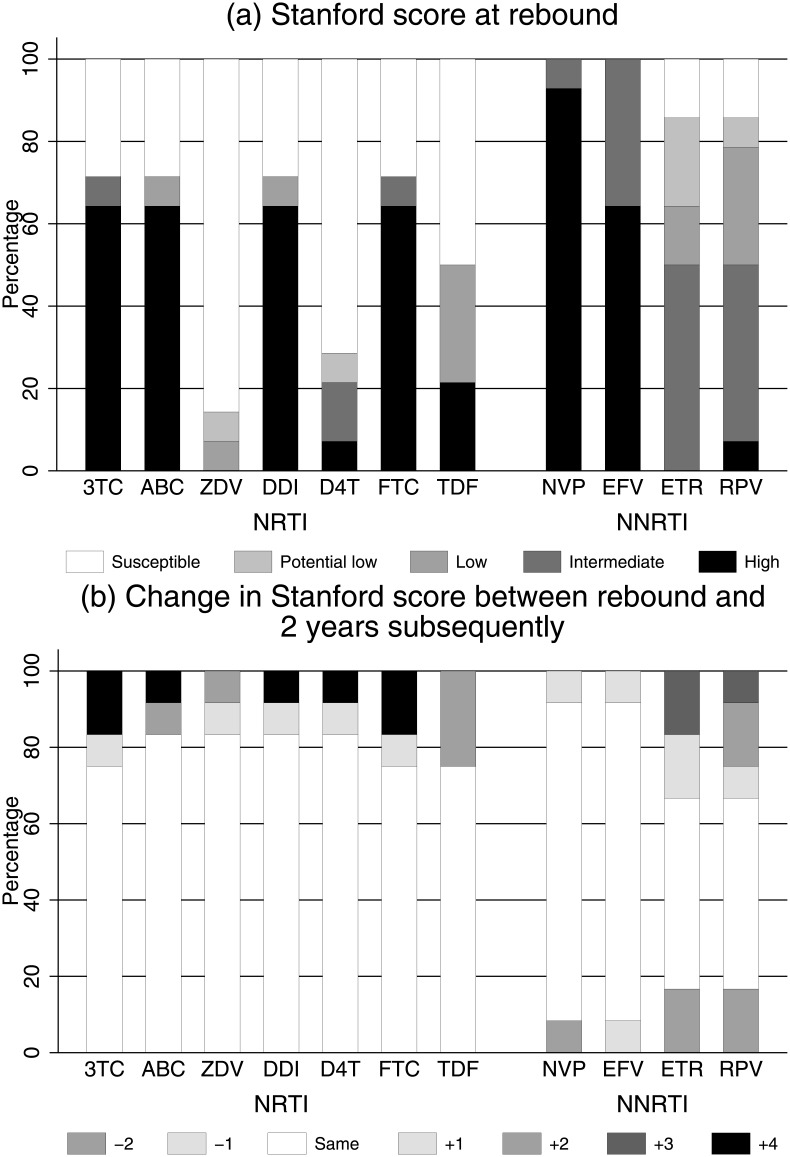
(a) Predicted drug susceptibility at rebound and (b) change in susceptibility over a median 2 years in those randomised to 2NRTI+NNRTI maintenance (*n* = 12). Abbreviations: 3TC, lamivudine; ABC, abacavir; D4T, stavudine; DDI, didanosine; EFV, efavirenz; ETR, etravirine; FTC, emtricitabine; NNRTI, non-nucleoside reverse transcriptase inhibitor; NRTI, nucleoside reverse transcriptase inhibitor; NVP, nevirapine; RPV, rilpivirine; TDF, tenofovir; ZDV, zidovudine.

In 12 children on 2NRTI+NNRTI with genotyping performed both at rebound and a median 2.3 (range 0.7–2.8) years subsequently, IAS-USA major NRTI mutations increased from a median (IQR) of 2 (0,2.5) to 2.5 (1.5,3.5) (median +1.0 [IQR 0.0,1.5] additional NRTI mutations per child; signed-rank *P* = 0.009). However, these additional NRTI mutations had little impact on predicted drug susceptibility, with only 1 child moving from no more than low-level to high-level resistance for both tenofovir and zidovudine (Fig 5). Only 5 children had repeated genotypes during pLLVL (median 2.1 [range 0.5–2.6] years between first and last genotype during pLLVL); none moved from no resistance or low-level resistance to intermediate or high-level resistance for tenofovir or zidovudine.

### VL resuppression without routine VL monitoring in children randomised to 2NRTI+NNRTI maintenance

Finally, we considered the implications of the children living with low-level VLs in the context of no VL monitoring or infrequent VL measurements, as occurs in ART programmes in low-income settings. Among children in the longitudinal cohort randomised to 2NRTI+NNRTI, 36 (18%) of 203 single VL measurements ≥1,000 copies/ml were immediately followed by a subsequent VL <1,000 copies/ml. However, this depended on the level of VL: whereas 19 (40%) of 48 single VLs 1,000–4,999 copies/ml were immediately followed by a VL<1,000 copies/ml, this occurred in 17 (11%) of 155 single VLs ≥5,000 copies/ml (chi-squared *P* < 0.001, S4 Table). Even with confirmed VL 1,000–4,999 (i.e., 2 consecutive values ≥1,000 copies/ml with the first <5,000 copies/ml), 4/35 (11%) were immediately followed by a VL <1,000 copies/ml. In contrast, although 7/155 (5%) of single VL ≥5,000 copies/ml immediately resuppressed <80 copies/ml, no measurements after confirmed rebound ≥5,000 copies/ml were <80 copies/ml.

## Discussion

There are few long-term longitudinal data on virological and resistance outcomes among children initiating first-line ART in sub-Saharan Africa. In this study, we compared virological outcomes in a large cohort of children with moderately advanced HIV disease receiving first-line ART without real-time VL monitoring in Uganda and Zimbabwe. Overall, virological outcomes were good, with almost three-quarters of children on current WHO-recommended abacavir+lamivudine+NNRTI regimens fully suppressed after 4 years of ART and only 6% switching to second-line treatment. We found no evidence of a difference in VL suppression or drug susceptibility between children managed with or without CD4 monitoring. Virological blips were relatively common, but pLLVL and rebound ≥5,000 copies/ml occurred in a minority; only 15% of child-time after week 24 was spent with viraemia in children taking NNRTI+2NRTI regimens. Even among children with VL >1,000 copies/ml, intermediate/high-level drug resistance to key second-line NRTIs was found only in a minority. Children with virological rebound did have a small increase in genotypic NRTI resistance over 2 years. Overall, these results suggest that, where available, annual VL monitoring as recommended by WHO is a reasonable and pragmatic approach in low- and middle-income countries.

The ARROW trial showed that CD4 monitoring (without routine VL monitoring) provided clinical benefit over clinical monitoring alone after the first year on ART. However, event rates were very low (new WHO stage 4 disease/death 0.4 per 100 child-years with CD4 monitoring versus 1.3 without) and 5-year survival was very high (96%). We have now extended these findings, by showing that virological suppression in the cohort as a whole was high. After a median of 4 years, 80% of children randomised to WHO-recommended 2NRTI+NNRTI–based regimens had VL <1,000 copies/ml and 74% had undetectable virus. This is similar to a recent small cohort of Kenyan children monitored for 4 years, in whom two-thirds maintained long-term virological control [16]. Virological suppression from most paediatric [17,18] cohorts falls short of the 90% target set by UNAIDS, highlighting the need for simpler, better-tolerated regimens and effective adherence interventions.

VL did not increase significantly over time in children with pLLVL or rebound; rather, it appeared that viraemia reached an equilibrium (approximately 1,000 and approximately 40,000 copies/ml, respectively), similar to the set-point that is characteristic of untreated HIV infection [19], perhaps due to the capacity for HIV-specific CD8+ T-cell responses to control viral replication on suboptimal ART [20], before potentially changing again to a new equilibrium in the rebound state, driven by further loss of adherence or by immune escape from previously effective T-cell responses [21]. Future studies of HIV-specific T-cell responses in ART-treated children are warranted to investigate these hypotheses further.

Children with virological failure in ARROW had frequent NNRTI and lamivudine resistance, as expected, but retained high susceptibility to both tenofovir and zidovudine, the recommended second-line NRTI options. Perhaps more importantly, a median of 2 years’ viral replication at approximately 40,000 copies/ml led to an increase of only 1 major NRTI mutation and did not substantively alter drug susceptibility. In the PENPACT-1 trial, which compared switch at VL 1,000 versus 30,000 copies/ml in Europe and the Americas, NNRTI resistance had already developed when VL reached >1,000 copies/ml [22], despite 3-monthly VL monitoring. Although NRTI thymidine analogue mutations accumulated with ongoing replication in the 30,000 copies/ml switch threshold group in PENPACT-1, this occurred less frequently in children taking abacavir+lamivudine. Children took a median 80 weeks for VL to increase from 1,000 to 30,000 copies/ml, but all those switching from 2NRTI+NNRTI to second-line at VL >30,000 copies/ml resuppressed within 24 weeks and had similar VL suppression at 5 years compared with those switching at >1,000 copies/ml. Similarly, responses to second-line therapy were good despite modest accumulation of resistance in the Kenyan study [16]. Even when failure is confirmed virologically, clinicians are often reluctant to switch ART because of limited and more complex second-line options (particularly appropriate formulations); limited access to resistance testing to inform decision-making; concerns regarding adherence; and, in resource-limited settings, very limited availability of third-line ART [23]. ARROW provides some reassurance that delays in switching of up to 2 years should have limited implications, particularly since the risk of onward transmission from children is low and the only large randomised trial addressing the role of NRTIs with substantial predicted cross-resistance in second-line treatment suggested they retained considerable activity in adults [7,24].

Over the longer term, substantial efforts are being made to ensure virological monitoring is available in all settings. However, challenges in training, procurement, specimen transport, and financial resources have limited its roll-out [25]. Coverage was estimated at 25% in 2014 [4] and is only estimated to increase to around 47% by 2019 [26]; furthermore, monitoring is mostly restricted to high-level facilities and after the first year is not recommended more often than yearly in most low-income countries. No virological monitoring was undertaken in ARROW; furthermore, VL suppression did not differ between children with or without CD4 monitoring, showing that good virological outcomes are achievable even with clinical monitoring, which can be done at low-level health facilities. Most children switching to second-line ART had detectable VL. Together, these findings highlight that absence of VL monitoring should not be a barrier to providing first-line or switching to second-line ART, given the considerable morbidity and mortality benefits of treatment.

Where virological monitoring is used, it is important to consider the implications of detectable viraemia in children. Current guidelines recommend switching to second-line ART at a confirmed VL above 1,000 copies/ml. However, we found that it was not uncommon for single, or even confirmed, VLs above this threshold to be immediately followed by return to values below the threshold without adherence counselling (since all VLs were measured retrospectively). This was particularly true for VLs between 1,000–5,000 copies/ml—precisely those levels where switch might be considered to have the greatest potential to prevent resistance accumulation. Resuppression on NNRTI-based regimens despite NNRTI resistance has previously been noted in cohorts [27] and a treatment interruption study [28]. This highlights the importance of maximising efforts to extend the durability of first-line ART, for example through enhanced adherence counselling and peer support, given detectable VL. Furthermore, it demonstrates the importance of repeating VL testing before switching from first-line to second-line ART in low-income countries, given the limited treatment regimens available, particularly where VL is 1,000–5,000 copies/ml. Importantly, no children with confirmed VL ≥5,000 copies/ml resuppressed <80 copies/ml. Outside a clinical trial such as ARROW, blips may also be caused by ART interruptions following drug stock-outs. Whilst blips have previously been shown to have no deleterious effect on subsequent VL rebound or CD4 responses [29], their impact on markers of underlying inflammation remains unclear in children and requires further study.

Study limitations include the fact that this was an exploratory analysis within a clinical trial that was conducted using 2006 WHO treatment thresholds, which have now been superseded by universal ART initiation criteria; whether virological outcomes would be different in children treated using current guidelines is unknown. However, given that VLs would be expected to be lower and CD4 percentages higher with earlier treatment, our results plausibly reflect a ‘worst-case’ scenario moving forwards. Genotyping was not conducted at ART initiation, and so may have missed preexisting resistance before ART. A reported 6.8% of those in the WHO African Region with available pre-ART genotypes had 1 or more mutations in any drug class before therapy initiation in 2010 [6], although prevalence of transmitted drug resistance is likely to be considerably lower here, since children were born between 1990–2008. Further resistance could have been identified by sequencing at lower thresholds; all genotyping was performed in Uganda based on a 1,000 copies/ml genotyping threshold. Our study was conducted within a clinical trial where drug supplies were continuous and participants received greater support than in a treatment programme; therefore, incidence of virological failure could have been lower. However, in contrast, most existing paediatric resistance data are from programmes/clinics with real-time VL monitoring; one might expect that resistance may be more prevalent without real-time VL monitoring.

In summary, we found that virological and resistance outcomes were good in this study, and that neither VL suppression nor predicted susceptibility of second-line treatment options were impacted by monitoring with or without CD4 counts over median 4 years follow-up in children initiating ART in Uganda and Zimbabwe. Despite being managed without real-time VL monitoring, only 20% of children on WHO-recommended 2NRTI+NNRTI first-line ART experienced pLLVL or rebound, during which time VL levels were stably maintained over relatively long periods. In rebound, genotypic resistance increased only slightly over 2 years, providing reassurance that annual VL monitoring, as recommended by WHO, is a reasonable and pragmatic approach for HIV-infected children on first-line ART in resource-limited settings.

## Supporting information

S1 Table Longitudinal cohort VLs and classification. Abbreviation: VL, viral load.
(XLS)

S2 Table Cross-sectional study VLs. Abbreviation: VL, viral load.
(XLS)

S3 Table VL response, blips, pLLVL, and rebound over weeks on ART by CD4 monitoring randomisation in 2NRTI+NNRTI. Abbreviations: ART, antiretroviral therapy; NNRTI, non-nucleoside reverse transcriptase inhibitor; NRTI, nucleoside reverse transcriptase inhibitor; pLLVL, persistent low-level viral load; VL, viral load.
(PDF)

S4 Table Probabilities of single VL measurements at different levels being followed 5–30 weeks later by a VL at various thresholds in children randomised to 2NRTI+NNRTI maintenance. Abbreviations: NNRTI, non-nucleoside reverse transcriptase inhibitor; NRTI, nucleoside reverse transcriptase inhibitor; VL, viral load.
(PDF)

S1 Fig VL suppression after median 4 years on ART according to different thresholds (a) by ART-regimen randomisation in all children and (b) by CD4 monitoring in 3NRTI. Abbreviations: ART, antiretroviral therapy; NRTI, nucleoside reverse transcriptase inhibitor; VL, viral load.
(PDF)

S2 Fig Prevalence of major IAS drug resistance mutations in 2NRTI+NNRTI maintenance (Arms A and B) with VL >1,000 copies/ml after a median of 4 years on ART. Abbreviations: ART, antiretroviral therapy; IAS, International AIDS Society; NNRTI, non-nucleoside reverse transcriptase inhibitor; NRTI, nucleoside reverse transcriptase inhibitor; VL, viral load.
(PDF)

S3 Fig Rates (per 100 child-years) of progression from VL response to blip, pLLVL, and rebound with (a) 2NRTI+NNRTI and (b) 3NRTI. Abbreviations: NNRTI, non-nucleoside reverse transcriptase inhibitor; NRTI, nucleoside reverse transcriptase inhibitor; pLLVL, persistent low-level viral load; VL, viral load.
(PDF)

S1 Appendix Appendix. (PDF)

S1 Protocol Protocol. (PDF)

S1 STROBE Checklist STROBE statement. (DOC)

## Abbreviations

ART: antiretroviral therapy
CDM: clinically driven monitoring
IAS: International AIDS Society
LCM: laboratory plus clinical monitoring
NNRTI: non-nucleoside reverse transcriptase inhibitor
NRTI: nucleoside reverse transcriptase inhibitor
pLLVL: persistent low-level viral load
UNAIDS: Joint United Nations Programme on HIV/AIDS
VL: viral load
