# Supplementary material for: Virological response and resistance among HIV-infected children receiving long-term antiretroviral therapy without virological monitoring in Uganda and Zimbabwe: Observational analyses within the randomised ARROW trial
Source: PLoS Med. 2017 Nov 14;14(11):e1002432. doi: 10.1371/journal.pmed.1002432 (PMC5685482; doi:10.1371/journal.pmed.1002432)
Supplement: S3 Table — Abbreviations: ART, antiretroviral therapy; NNRTI, non-nucleoside reverse transcriptase inhibitor; NRTI, nucleoside reverse transcriptase inhibitor; pLLVL, persistent low-level viral load; VL, viral load. (PDF) [file pmed.1002432.s003.pdf]

S3 Table. VL response, blips, pLLVL and rebound over weeks on ART by CD4 monitoring randomisation in 2NRTI+NNRTI

	Week 4	Week 24	Week 36	Week 48	Week 72	Week 96	Week 120	Week 144	Week 168	Week 192	Week 216
**CD4-monitoring**	N=104	N=104	N=104	N=104	N=104	N=104	N=104	N=104	N=104	N=104	N=104
VL response	104 (100.0%)	100 (96.2%)	90 (86.5%)	84 (80.8%)	67 (64.4%)	62 (59.6%)	54 (51.9%)	42 (40.4%)	38 (36.5%)	35 (33.7%)	35 (33.7%)
Current/previous blip	0 (0.0%)	0 (0.0%)	5 (4.8%)	7 (6.7%)	19 (18.3%)	23 (22.1%)	30 (28.9%)	41 (39.4%)	43 (41.3%)	45 (43.3%)	45 (43.3%)
pLLVL	0 (0.0%)	0 (0.0%)	4 (3.8%)	7 (6.7%)	9 (8.7%)	10 (9.6%)	8 (7.7%)	8 (7.7%)	9 (8.7%)	10 (9.6%)	10 (9.6%)
Rebound	0 (0.0%)	4 (3.8%)	5 (4.8%)	6 (5.8%)	9 (8.7%)	9 (8.7%)	12 (11.5%)	13 (12.5%)	14 (13.5%)	14 (13.5%)	14 (13.5%)
**no CD4-monitoring**	N=100	N=100	N=99	N=99	N=99	N=99	N=99	N=99	N=98	N=98	N=98
VL response	100 (100.0%)*	99 (99.0%)	84 (84.8%)	76 (76.8%)	60 (60.6%)	53 (53.5%)	50 (50.5%)	44 (44.4%)	42 (42.9%)	38 (38.8%)	38 (38.8%)
Current/previous blip	0 (0.0%)	0 (0.0%)	4 (4.0%)	11 (11.1%)	26 (26.3%)	33 (33.3%)	35 (35.4%)	39 (39.4%)	40 (40.8%)	44 (44.9%)	44 (44.9%)
pLLVL	0 (0.0%)	0 (0.0%)	4 (4.0%)	4 (4.0%)	3 (3.0%)	3 (3.0%)	2 (2.0%)	3 (3.0%)	3 (3.1%)	3 (3.1%)	4 (3.1%)
Rebound	0 (0.0%)	1 (1.0%)	7 (7.1%)	8 (8.1%)	10 (10.1%)	10 (10.1%)	12 (12.1%)	13 (13.1%)	13 (13.3%)	13 (13.3%)	13 (13.3%)
Exact p	P=1.00	P=0.37	P=0.92	P=0.53	P=0.23	P=0.10	P=0.27	P=0.55	P=0.38	P=0.30	P=0.30

Carrying forwards current state where VL missing; carrying backwards, “VL response,” where week 4 missing (n = 16)
Note: pLLVL=persistent low level VL. P-values from exact tests.
