# Supplementary material for: Virological response and resistance among HIV-infected children receiving long-term antiretroviral therapy without virological monitoring in Uganda and Zimbabwe: Observational analyses within the randomised ARROW trial
Source: PLoS Med. 2017 Nov 14;14(11):e1002432. doi: 10.1371/journal.pmed.1002432 (PMC5685482; doi:10.1371/journal.pmed.1002432)
Supplement: S4 Table — Abbreviations: NNRTI, non-nucleoside reverse transcriptase inhibitor; NRTI, nucleoside reverse transcriptase inhibitor; VL, viral load. (PDF) [file pmed.1002432.s004.pdf]

S4 Table. Probabilities of single VL measurements at different levels being followed 5-30 weeks later by a VL at various thresholds in children randomised to 2NRTI+NNRTI maintenance

		Subsequent viral load (copies/ml)						
		**<80**	**80-199**	**200-399**	**400-999**	**1000-4999**	**≥5000**	**Total**
**Single viral load (copies/ml)**	**<80**	990 (87.4)	72 (6.4)	21 (1.9)	17 (1.5)	18 (1.6)	15 (1.3)	1133 (100.0)
	**80-199**	85 (82.5)	5 (4.9)	3 (2.9)	2 (1.9)	2 (1.9)	6 (5.8)	103 (100.0)
	**200-399**	29 (72.5)	4 (10.0)	1 (2.5)	1 (2.5)	4 (10.0)	1 (2.5)	40 (100.0)
	**400-999**	15 (46.9)	1 (3.1)	4 (12.5)	4 (12.5)	4 (12.5)	4 (12.5)	32 (100.0)
	**1000-4999**	14 (29.2)	1 (2.1)	2 (4.2)	2 (4.2)	16 (33.3)	13 (27.1)	48 (100.0)
	**≥5000**	7 (4.5)	5 (3.2)	3 (1.9)	2 (1.3)	13 (8.4)	125 (80.6)	155 (100.0)
