# Supplementary material for: Virological response and resistance among HIV-infected children receiving long-term antiretroviral therapy without virological monitoring in Uganda and Zimbabwe: Observational analyses within the randomised ARROW trial
Source: PLoS Med. 2017 Nov 14;14(11):e1002432. doi: 10.1371/journal.pmed.1002432 (PMC5685482; doi:10.1371/journal.pmed.1002432)
Supplement: S1 Fig — Abbreviations: ART, antiretroviral therapy; NRTI, nucleoside reverse transcriptase inhibitor; VL, viral load. (PDF) [file pmed.1002432.s005.pdf]

S1 Fig. VL suppression after median 4 years on ART according to different thresholds

(a) by ART-regimen randomisation in all children (b) by CD4 monitoring in 3NRTI

(a) by ART-regimen randomisation in all children

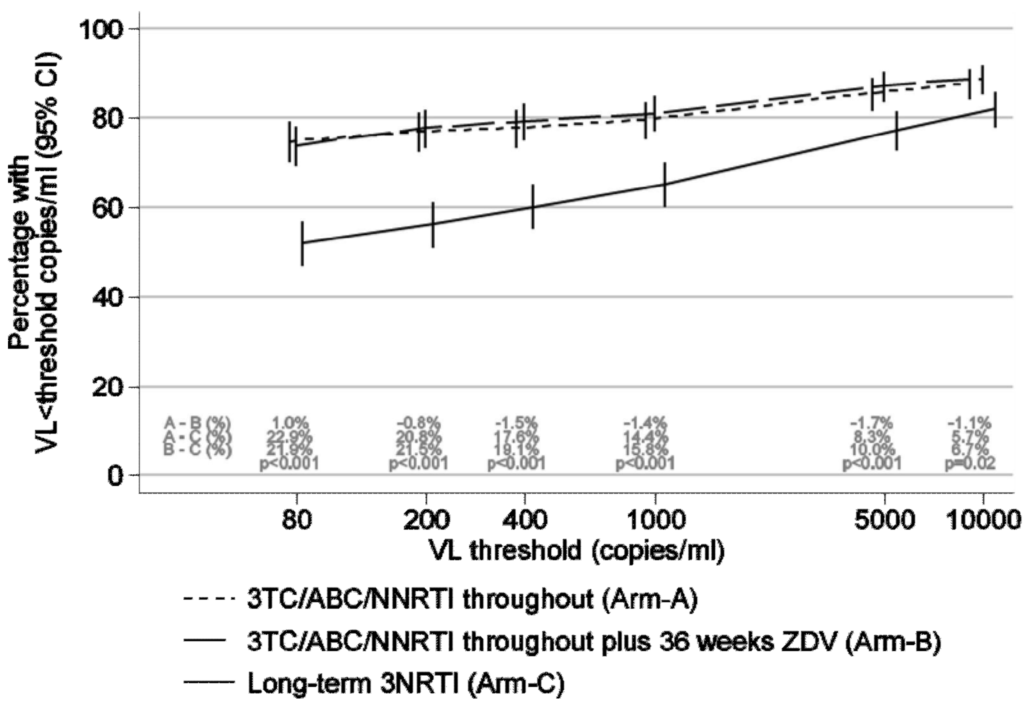

(b) by CD4 monitoring in 3NRTI

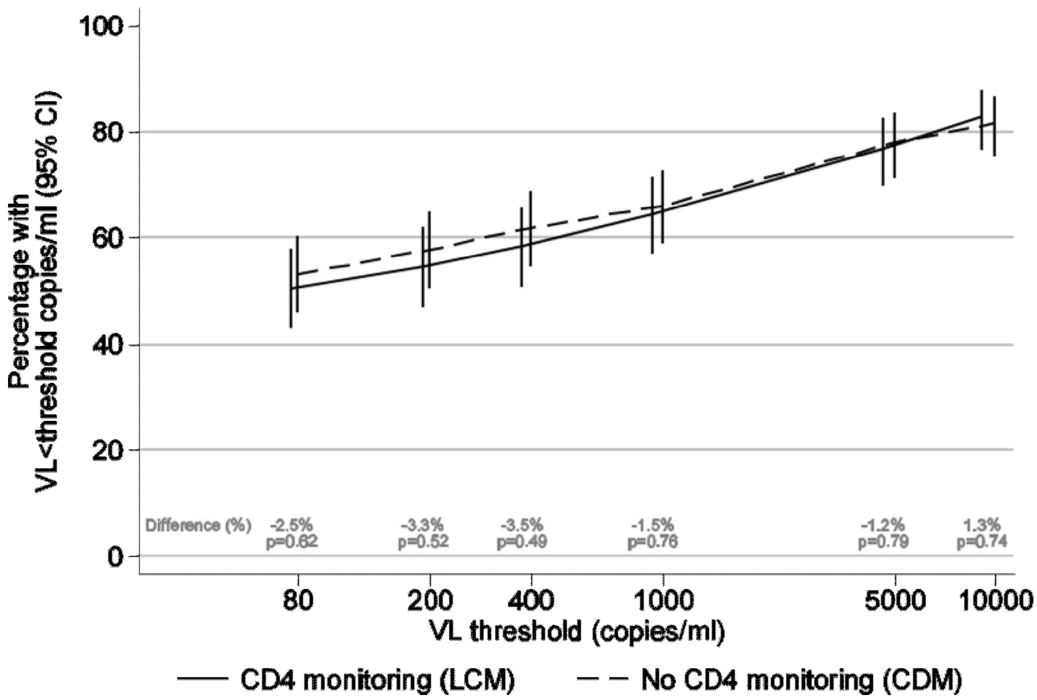

Note: impact of CD4 monitoring in 2NRTI+NNRTI shown in main Fig. 1.
