# Supplementary material for: Virological response and resistance among HIV-infected children receiving long-term antiretroviral therapy without virological monitoring in Uganda and Zimbabwe: Observational analyses within the randomised ARROW trial
Source: PLoS Med. 2017 Nov 14;14(11):e1002432. doi: 10.1371/journal.pmed.1002432 (PMC5685482; doi:10.1371/journal.pmed.1002432)
Supplement: S2 Fig — Abbreviations: ART, antiretroviral therapy; IAS, International AIDS Society; NNRTI, non-nucleoside reverse transcriptase inhibitor; NRTI, nucleoside reverse transcriptase inhibitor; VL, viral load. (PDF) [file pmed.1002432.s006.pdf]

S2 Fig. Prevalence of major IAS drug resistance mutations in 2NRTI+NNRTI maintenance (Arms A and B) with VL>1000 copies/ml after median 4 years on ART

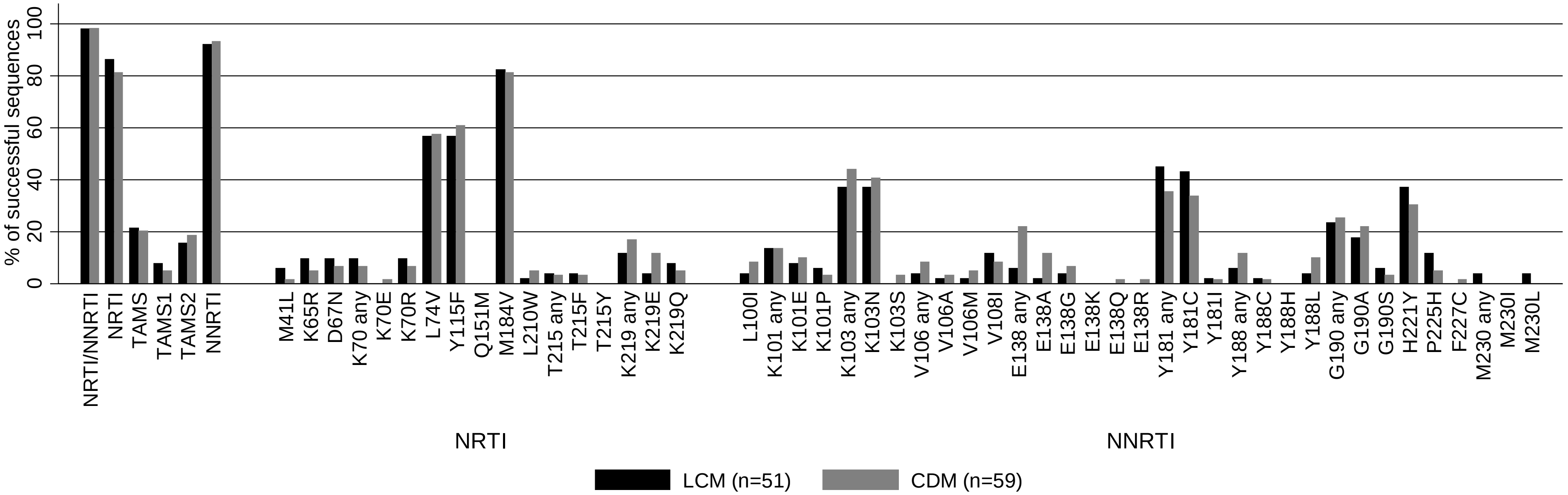

LCM vs CDM:
E138 any: p=0.03; E138A: p=0.07. All others p>0.1
LCM=CD4 monitoring, CDM=no CD4 monitoring

Note: impact of 2NRTI+NNRTI on IAS drug resistance mutations shown in S1 Fig.
