# Supplementary material for: Virological response and resistance among HIV-infected children receiving long-term antiretroviral therapy without virological monitoring in Uganda and Zimbabwe: Observational analyses within the randomised ARROW trial
Source: PLoS Med. 2017 Nov 14;14(11):e1002432. doi: 10.1371/journal.pmed.1002432 (PMC5685482; doi:10.1371/journal.pmed.1002432)

S3 Fig. Rates (per 100 child-years) of progression from VL response to blip, pLLVL and rebound with (a) 2NRTI+NNRTI (b) 3NRTI

(a) 2NRTI+NNRTI

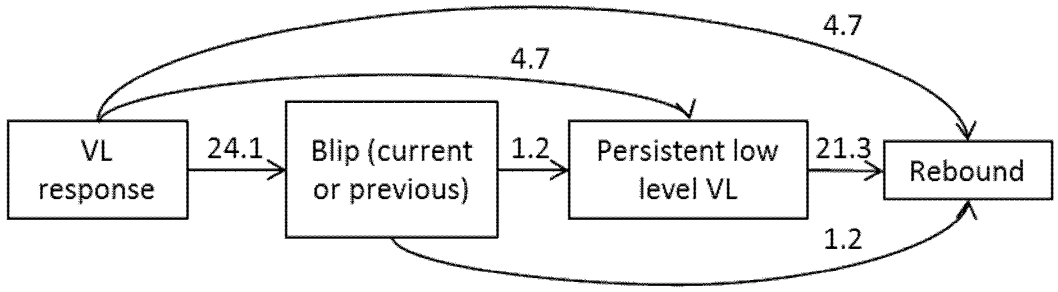

(b) 3NRTI

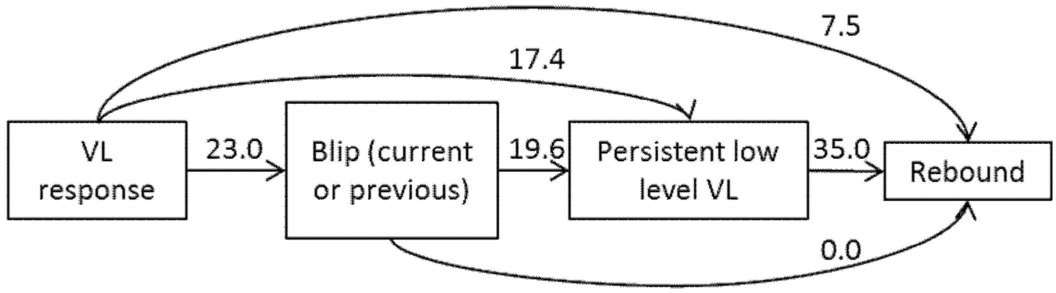

Supplement: S3 Fig — Abbreviations: NNRTI, non-nucleoside reverse transcriptase inhibitor; NRTI, nucleoside reverse transcriptase inhibitor; pLLVL, persistent low-level viral load; VL, viral load. (PDF) [file pmed.1002432.s007.pdf]
