# Supplementary material for: Virological response and resistance among HIV-infected children receiving long-term antiretroviral therapy without virological monitoring in Uganda and Zimbabwe: Observational analyses within the randomised ARROW trial
Source: PLoS Med. 2017 Nov 14;14(11):e1002432. doi: 10.1371/journal.pmed.1002432 (PMC5685482; doi:10.1371/journal.pmed.1002432)

Contents

Supplementary Methods 2

Supplementary Results 3

Table A1. Characteristics at ART initiation of all children in the trial, all children followed in the longitudinal cohort and all those subsequently experiencing viral load blips, low level viral load and/or rebound 7

Table A2. VL response, blips, pLLVL and rebound over weeks on ART by CD4 monitoring randomisation in 3NRTI 9

Table A3. VL at pLLVL and rebound and subsequent change over time: all children 10

Table A4. Independent predictors of children experiencing VL blips, pLLVL, rebound and either pLLVL or rebound 11

Table A5. Probabilities of (a) single and (b) confirmed VL measurements at different levels being followed 5-30 weeks later by a VL at various thresholds: all children 13

Fig A1. Overall predicted NRTI and NNRTI susceptibility in 3NRTI (Arm C) with VL>1000 copies/ml after median 4 years on ART by CD4 monitoring strategy (n=108) 14

Fig A2. Prevalence of major IAS drug resistance mutations in triple NRTI maintenance (Arm-C) with VL>1000 copies/ml after median 4 years on ART 15

Fig A3. Viral load response in 107 children randomised to 3NRTI initially responding to ART . 16

Supplementary Methods

Virological definitions

Viral load (VL) measurements ≥80 copies/ml were classified as a blip if the child had a previous VL <80 copies/ml and either (1) subsequently re-suppressed <80 copies/ml (one or two VL≥80 copies/ml allowed; n=150) or (2) their last measurement was a single value ≥80 copies/ml (n=31). The latter is because, firstly, blips were much more common than pLLVL, and so, on the balance of probability, these observations would be more likely to be blips than pLLVL. Secondly, excluding them from analyses completely (effectively ignoring this last VL observation) would necessarily provide an estimate of the percentage of children experiencing blips that was biased downwards, because at least some of these children would indeed have blipped (and a smaller percentage of others would have pLLVL). Therefore by including them all as blips, the percentage of children who remain complete VL responders is not over-estimated.

Predictors of children experiencing different patterns of virological control

In the longitudinal cohort of 316 children, predictors of children ever experiencing VL blips, persistent low-level VL (pLLVL, as defined in main Methods), rebound ≥5000 copies/ml (confirmed) and either pLLVL or rebound were identified using logistic regression. As a first step, backward elimination with exit p=0.10 was used on pre-ART factors (as shown in Table 1), forcing the randomised groups into the models. Weight-for-age was not considered because it was highly associated with height-for-age (ρ=0.73). Final models fitted to each of the four VL outcomes included all predictors identified for any outcome to enable comparison of effects across outcomes.

Supplementary Results

VL in children who died

15 (3 CD4-monitoring, 12 no CD4-monitoring; 12 2NRTI+NNRTI maintenance, 3 3NRTI) children died after 48 weeks on ART; all had VL assayed within 6 months prior to death. Two (1 CD4-monitoring, 1 no CD4-monitoring; both 2NRTI+NNRTI maintenance) were <80 copies/ml; in the remainder the median VL was 49800 copies/ml, with a trend towards lower VL in the 2 children dying in CD4-monitoring (median 5060 copies/ml) compared to the 11 dying in no CD4-monitoring (median 80770 copies/ml; ranksum p=0.08) (median 43010 copies/ml 2NRTI+NNRTI maintenance, 80770 copies/ml 3NRTI, ranksum p=0.50).

VL in children who switched to ritonavir-boosted protease-inhibitor-containing regimens

67 (6%) children switched to ritonavir-boosted protease-inhibitor-containing regimens during follow-up, 4 for toxicity and 63 for first-line clinical/immunological failure after a median 120 weeks on ART (range 49-247). 35 (6%) CD4-monitoring vs 28 (5%) no CD4-monitoring switched for first-line clinical/immunological failure (exact p=0.37). 43 (5%) 2NRTI+NNRTI vs 20 (5%) 3NRTI switched for first-line clinical/immunological failure (exact p=0.79). There were non-significant trends towards higher VLs at switch in children monitored using CD4s: 5 (14%) monitored with and 9 (32%) monitored without CD4s had VL<5000 copies/ml at switch (chi-squared p=0.09); 4 (11%) and 7 (25%) had VL <1000 copies/ml (p=0.16) and 3 (9%) and 6 (21%) <80 copies/ml (p=0.15). There was no evidence of a difference according to ART regimen: 8 (19%) 2NRTI+NNRTI vs 6 (30%) 3NRTI had VL<5000 copies/ml at switch (chi-squared p=0.31)

Long-term virological response (cross-sectional study) (all children)

There was no evidence of a difference in long-term virological suppression between 3NRTI children randomised to monitoring with or without CD4s (p>0.4, S1 Fig). There was also no evidence of differences between monitoring groups in intermediate/high level resistance to

NRTI/NNRTI (exact p>0.1) except for zidovudine, where a greater proportion monitored using CD4s had intermediate/high level resistance than those monitored without CD4s (p=0.02) (Fig A1). Prevalence of IAS-USA NRTI or NNRTI mutations was also similar (Fig A2).

VL completeness in the longitudinal cohort

Of the 316 children in the complete longitudinal cohort (2NRTI+NNRTI and 3NRTI) (Table A1), 3(1%) children died before week-4 and had only baseline VL. Of the remaining 313 children, 311(99%) achieved an initial VL response and were included in subsequent analyses. Only 4 (1%) children switched to second-line and 6 (2%) died after week-4 (4 VL responders, 2 in rebound).

Of the 311 initial VL responders in the longitudinal cohort, VLs were available for 99.7%(n=310) at ART initiation; 92.9%(n=289), 85.4%(n=264), 97.1%(n=298) and 94.8%(n=291) of those alive and in follow-up at weeks 4, 24, 36 and 48 post-ART initiation, respectively; and 90.6% of subsequent 24-weekly VLs on first-line ART.

VL control over time

In contrast to the stable percentage of children randomised to 2NRTI+NNRTI with either pLLVL or VL rebound after 72 weeks on ART (Figure 3(a)), among children on 3NRTI the percentages with pLLVL or rebound continued to increase throughout follow-up (Fig A3(a)). Overall, post-week-24, children randomised to 3NRTI spent 49% of child-time as complete VL responders and 67% as either complete VL responders or having had one or more blips; 16% of time was spent with pLLVL and 18% with rebound ≥5000 copies/ml. There was a trend towards fewer children randomised to 3NRTI experiencing blips compared to 2NRTI+NNRTI (37(35%) 3NRTI vs 93 (46%) 2NRTI+NNRTI; exact p=0.07), possibly because of the greater rates of pLLVL and rebound with 2NRTI which meant that children did not have blips recorded. However, the median (IQR) VL during blips was similarly low at 190 (100-1440) copies/ml. As for the cross-sectional cohort, there was no evidence of any difference in VL response between those

monitored with and without CD4s throughout (p>0.2, Table A2).

Overall 39 (36%) children randomised to 3NRTI experienced pLLVL, significantly more than 2NRTI+NNRTI (20 (10%); p<0.001). Geometric mean VL at the start of pLLVL was 1250 copies/ml [95%CI 832-1890] for 3NRTI, similar to 2NRTI+NNRTI, and did not increase over the subsequent median 85 weeks (IQR 35,>157) spent with pLLVL (change -0.16 log10 per year (95% CI -0.36 to +0.04) p=0.12; Fig A3(b)). In addition, children randomised to 3NRTI with pLLVL also experienced VL re-supression; 22/153 (14%) measurements in pLLVL were <80 copies/ml, albeit this re-suppression was usually transient. 31 (29%) children randomised to 3NRTI experienced rebound to ≥5000 copies/ml, significantly more than 2NRTI+NNRTI (28 (14%); p=0.002). Geometric mean VL at rebound was 31,200 copies/ml [95%CI 20,100-48,420] in 3NRTI, similar to 2NRTI+NNRTI. VL did not increase over the median of 72 weeks (IQR 24,93) spent with rebound (change -0.12 log10 per year (95% CI -0.27 to +0.02, p=0.10; Fig A3(b)). In contrast to pLLVL, no measurements in rebound were <80 copies/ml.

In joint models including all 311 children in the longitudinal study with initial VL response (2NRTI+NNRTI and NNRTI), there was no evidence that the initial VL at pLLVL/rebound, or the subsequent rate of VL change, differed between 2NRTI+NNRTI and 3NRTI (p>0.1, Table A3).

Independent predictors of loss of VL control in all children

In multivariable models including all children (Table A4), children remained independently more likely to experience low-level VL/rebound on triple NRTI than on 2NRTI+NNRTI maintenance (adjusted-p<0.001) and if ART was initiated in older childhood (adjusted-p=0.02) or with higher VL (adjusted-p=0.04). Conversely, children were slightly more likely to experience blips on 2NRTI+NNRTI than 3NRTI (adjusted-p=0.04). There was marginal evidence that children were less likely to experience pLLVL/rebound if ART was initiated with higher CD4% (adjusted-p=0.097), or if the child/mother had previously received single-dose nevirapine for prevention of transmission (adjusted-p=0.06, similarly to observed previously in this cohort); children were

less likely to experience blips if ART was initiated in older childhood (adjusted-p=0.06) or their primary carer was their mother (adjusted-p=0.051). Interestingly, on 2NRTI+NNRTI maintenance, there was a suggestion that those receiving additional zidovudine induction for the first 36 weeks were less likely to rebound (adjusted-p=0.07; 10% (11/109) compared with 18% (17/95) of children on 2NRTI+NNRTI throughout).

VL re-suppression without routine VL monitoring in all children

Among all children in the longitudinal cohort (randomised to 2NRTI+NNRTI or 3NRTI), 18% of single VL measurements ≥1000 copies/ml were immediately followed by a subsequent VL <1000 copies/ml. However, this depended on the level of VL: whereas 35% of single VLs 1000-4999 copies/ml were immediately followed by a VL<1000 copies/ml, this occurred in only 11% of all single VLs ≥5000 copies/ml (Table A5).

Table A1. Characteristics at ART initiation of all children in the trial, all children followed in the longitudinal cohort and all those subsequently experiencing viral load blips, low level viral load and/or rebound

Factor (at ART initiation)	All ARROW children N=1206	All longitudinal cohort N=311* Median (IQR) or n (%)	Ever experienced viral load blips N=130 Median (IQR) or n (%)	Ever experienced low level viral load N=59 Median (IQR) or n (%)	Ever experienced rebound N=59 Median (IQR) or n (%)
Male	596 (49.4%)	146 (46.9%)	62 (47.7%)	25 (42.4%)	29 (49.2%)
Centre					
A	400 (33.2%)	104 (33.4%)	64 (49.2%)	18 (30.5%)	13 (22.0%)
B	188 (15.6%)	50 (16.1%)	14 (10.8%)	6 (13.6%)	13 (22.0%)
C	318 (26.4%)	79 (25.4%)	26 (20.0%)	18 (30.5%)	15 (25.4%)
D	300 (24.9%)	78 (25.1%)	26 (20.0%)	15 (25.4%)	18 (30.5%)
**At ART initiation**					
Age (years)	6.0 (2.4, 9.3)	5.4 (2.2, 9.4)	5.3 (2.3, 8.4)	6.5 (2.7, 10.2)	7.8 (1.7, 12.3)
Weight-for-age**	-2.2 (-3.3, -1.3)	-2.2 (-3.3, -1.3)	-2.2 (-3.3, -1.5)	-2.1 (-3.9, -1.5)	-2.2 (-3.3, -1.5)
Height-for-age**	-2.4 (-3.4, -1.5)	-2.5 (-3.4, -1.6)	-2.8 (-3.4, -1.9)	-2.5 (-3.4, -1.8)	-2.1 (-3.3, -1.3)
Viral load (copies/ml)	268800 (89200, 748700)[0]	214800 (67000, 641200)[1]	278000 (94300, 704300)[1]	249100 (92200, 726200)	275100 (94800, 1039200)
CD4%	12 (7, 17)	13 (8, 19)	13 (10, 18)	13 (9, 16)	10 (5, 15)
WHO stage					
1/2	354 (29.4%)	100 (32.2%)	39 (30.0%)	17 (28.8%)	17 (28.8%)
3/4	852 (70.6%)	211 (67.8%)	91 (70.0%)	42 (71.2%)	42 (71.2%)
Monitoring					
CD4 monitoring	600 (49.8%)	154 (49.5%)	64 (49.2%)	30 (50.8%)	30 (50.8%)
No CD4 monitoring	606 (50.2%)	157 (50.5%)	66 (50.8%)	29 (49.2%)	29 (49.2%)
ART strategy					
Arm A (3TC/ABC/NNRTI throughout)	397 (32.9%)	95 (30.5%)	44 (33.8%)	11 (18.6%)	17 (28.8%)
Arm B (36 weeks ZDV)	404 (33.5%)	109 (35.0%)	49 (37.7%)	9 (15.3%)	11 (18.6%)
Arm C (long-term ZDV, 3NRTI after week-36)	405 (33.6%)	107 (34.4%)	37 (28.5%)	39 (66.1%)	31 (52.5%)
Initial NNRTI					
Nevirapine	758 (62.9%)	178 (57.2%)	80 (61.5%)	33 (55.9%)	29 (49.2%)
Efavirenz	448 (37.1%)	133 (42.8%)	50 (38.5%)	26 (44.1%)	30 (50.8%)

Previous single dose nevirapine to mother and/or child for prevention of transmission	92 (7.6%)	25 (8.0%)	14 (10.8%)	1 (1.7%)	2 (3.4%)
Primary carer					
Mother	674 (55.9%)[2]	178 (57.2%)	70 (53.8%)	34 (57.6%)	34 (57.6%)
Other	530 (43.9%)	133 (42.8%)	60 (46.2%)	25 (42.4%)	25 (42.4%)

* excluding 3 children who died before 4 weeks (with baseline VL only) and 2 non-responders (never achieved VL <9500 c/ml)

** Height-for-age calculated using WHO reference[14]; as weight-for-age only covers children <121 months, this was calculated using the UK reference which covers the full age range of ARROW children[15] (Spearman correlation between UK and WHO references=0.99 in n=971 children <121 months)

[0] assayed for 867/1206 (72%) children

[1] missing for 1 child

[2] missing for 2 children

Note: Children experiencing more than one of viral load blips, low-level viral load and rebound included in all relevant columns

Table A2. VL response, blips, pLLVL and rebound over weeks on ART by CD4 monitoring randomisation in 3NRTI

	Week 4	Week 24	Week 36	Week 48	Week 72	Week 96	Week 120	Week 144	Week 168	Week 192	Week 216
**CD4-monitoring**	N=50	N=48	N=48	N=48	N=48	N=48	N=48	N=48	N=48	N=48	N=48
VL response	49 (98.0%)*	46 (95.8%)	34 (70.8%)	33 (68.8%)	24 (50.0%)	19 (39.6%)	18 (37.5%)	14 (29.2%)	13 (27.1%)	11 (22.9%)	11 (22.9%)
Current/previous blip	0 (0.0%)	0 (0.0%)	3 (6.3%)	3 (6.3%)	7 (14.6%)	10 (20.8%)	9 (18.8%)	12 (25.0%)	13 (27.1%)	14 (29.2%)	14 (29.2%)
pLLVL	0 (0.0%)	0 (0.0%)	8 (16.7%)	7 (14.6%)	10 (20.8%)	11 (22.9%)	10 (20.8%)	8 (16.7%)	6 (12.5%)	7 (14.6%)	7 (14.6%)
Rebound	0 (0.0%)	2 (4.2%)	3 (6.3%)	5 (10.4%)	7 (14.6%)	8 (16.7%)	11 (22.9%)	14 (29.2%)	16 (33.3%)	16 (33.3%)	16 (33.3%)
**no CD4-monitoring**	N=57	N=57	N=56	N=56	N=55	N=55	N=55	N=55	N=55	N=55	N=55
VL response	56 (98.2%)*	55 (96.5%)	47 (83.9%)	44 (78.6%)	27 (49.1%)	12 (38.2%)	20 (36.4%)	18 (32.7%)	16 (29.1%)	15 (27.3%)	15 (27.3%)
Current/previous blip	0 (0.0%)	0 (0.0%)	1 (1.8%)	3 (5.4%)	13 (23.6%)	16 (29.1%)	12 (21.8%)	13 (23.6%)	12 (21.8%)	12 (21.8%)	12 (21.8%)
pLLVL	0 (0.0%)	0 (0.0%)	4 (7.1%)	4 (7.1%)	8 (14.5%)	8 (14.5%)	12 (21.8%)	11 (20.0%)	13 (23.6%)	14 (25.5%)	14 (25.5%)
Rebound	0 (0.0%)	2 (3.5%)	4 (7.1%)	5 (8.9%)	7 (12.7%)	10 (18.2%)	11 (20.0%)	13 (23.6%)	14 (25.5%)	14 (25.5%)	14 (25.5%)
Exact p	P=1.00	P=1.00	P=0.27	P=0.60	P=0.66	P=0.65	P=0.98	P=0.90	P=0.46	P=0.44	P=0.44

Carrying forwards current state where VL missing; carrying backwards, "VL response," where week 4 missing (n = 6)

*Two children did not achieve an initial VL response at week 4 but achieved response at week 24

Note: pLLVL=persistent low level VL. P-values from exact tests. P>0.08 pooling all ART strategy groups and comparing CD4-monitoring vs no CD4 monitoring in all randomised children.

Table A3. VL at pLLVL and rebound and subsequent change over time: all children

	pLLVL				Rebound			
	Estimate (95% CI)	p	Estimate (95% CI)	p	Estimate (95% CI)	p	Estimate (95% CI)	p
Log10 VL at start								
Overall	3.02 (2.87,3.16)	P<0.001			4.55 (4.42,4.69)	P<0.001		
2NRTI+NNRTI	-		2.88 (2.64,3.13)	P<0.001	-		4.62 (4.43,4.81)	P<0.001
3NRTI	-		3.10 (2.91,3.28)	P<0.001	-		4.50 (4.31,4.68)	P<0.001
			Heterogeneity p=0.17				Heterogeneity p=0.37	
Change in log10 VL per year								
Overall	-0.09 (-0.22,+0.04)	P=0.19			-0.11 (-0.22,-0.01)	P=0.04		
2NRTI+NNRTI	-		+0.00 (-0.19,+0.20)	P=0.96	-		-0.10 (-0.26,+0.05)	P=0.18
3NRTI	-		-0.15 (-0.33,+0.03)	P=0.10	-		-0.12 (-0.28,+0.03)	P=0.12
			Heterogeneity p=0.24				Heterogeneity p=0.86	

Note: table shows results for log10 VL, the scale on which the modelling was performed. Absolute values at the start of pLLVL/rebound are back-transformed to the absolute scale (effectively being a geometric mean) in the main Results text.

Table A4. Independent predictors of children experiencing VL blips, pLLVL, rebound and either pLLVL or rebound

	VL blips		pLLVL		Rebound		pLLVL or rebound	
	**aOR [95% CI]**	**p**	**aOR [95% CI]**	**P**	**aOR [95% CI]**	**p**	**aOR [95% CI]**	**p**
Age at ART initiation (per year older)	0.93 [0.87-1.00]	0.06	1.06 [0.97-1.16]	0.18	1.12 [1.02-1.23]	0.02	1.10 [1.01-1.19]	0.02
VL at ART initiation (per log_{10} higher)	1.32 [0.87-1.99]	0.19	1.52 [0.87-2.66]	0.14	1.60 [0.91-2.80]	0.102	1.67 [1.04-2.69]	0.04
CD4% at ART initiation (per 10% higher)	1.33 [0.94-1.89]	0.11	0.96 [0.61-1.49]	0.85	0.51 [0.33-0.81]	0.004	0.72 [0.49-1.06]	0.097
CD4 monitoring, vs no CD4 monitoring	1.15 [0.70-1.88]	0.58	0.91 [0.49-1.71]	0.77	0.88 [0.47-1.64]	0.69	0.89 [0.52-1.53]	0.67
ART strategy, vs Arm C (3NRTI maintenance)		0.11*		<0.001		<0.001		<0.001
Arm A (2NRTI+NNRTI throughout)	1.88 [1.01-3.50]	0.046	0.20 [0.09-0.43]	<0.001	0.47 [0.23-0.97]	0.04	0.29 [0.15-0.56]	<0.001
Arm B (36 weeks ZDV, 2NRTI+NNRTI maintenance)**	1.61 [0.89-2 92]	0.12	0.13 [0.06-0.30]	<0.001	0.21 [0.09-0.48]	<0.001	0.16 [0.08-0 32]	<0.001
Previous single dose nevirapine to mother and/or child for prevention of transmission	2.00 [0.78-5.12]	0.15	0.11 [0.01-0.88]	0.04	0.42 [0.08-2.06]	0.28	0.28 [0.07-1.07]	0.06
Mother as primary carer, vs other	0.58 [0.33-1.00]	0.051	1.21 [0.60-2.43]	0.59	1.33 [0.66-2.68]	0.43	1.11 [0.61-2.02]	0.73
Centre, vs. A		<0.001		0.71		0.02		0.07
B	0.21 [0.09-0.49]	<0.001	0.79 [0.29-2.21]	0.66	3.93 [1.48-10.44]	0.006	2.41 [1.01-5.74]	0.047
C	0.28 [0.15-0.53]	<0.001	1.39 [0.62-3.08]	0.42	1.83 [0.78-4.30]	0.16	2.16 [1.06-4.41]	0.03
D	0.24 [0.12-0.46]	<0.001	1.21 [0.52-2.81]	0.66	2.86 [1.22-6.67]	0.02	2.09 [1.00-4.35]	0.0501

Backwards elimination on pre-ART factors (shown in Table 1; exit p=0.10). Final models fitted to each of the four outcomes (VL blips, pLLVL, rebound, either pLLVL or rebound) included all predictors identified for any outcome to enable comparison of effects. Weight-for-age was not considered because it was highly associated with height-for-age (ρ=0.73)

*Arms A/B combined (2NRTI+NNRTI maintenance) vs Arm C (3NRTI maintenance): aOR=0.58 [0.34-0.98] p=0.04

**Arm B (36 weeks ZDV, 2NRTI+NNRTI maintenance) vs Arm A (2NRTI+NNRTI throughout): p=0.61 (VL blips), p=0.38 (pLLVL), p=0.07 (rebound; aOR=0.45 [0.19-1.08]), p=0.11 (pLLVL or rebound)

aOR=adjusted odds ratio

Table A5. Probabilities of (a) single and (b) confirmed VL measurements at different levels being followed 5-30 weeks later by a VL at various thresholds: all children

(a) Probability of single VL measurement ≥80, ≥200, ≥400, ≥1000 and ≥5000 copies/ml resuppressing < various thresholds

		Subsequent viral load (copies/ml)						
		**<80**	**80-199**	**200-399**	**400-999**	**1000-4999**	**≥5000**	**Total**
**Single viral load (copies/ml)**	**<80**	1347 (86.0)	99 (6.3)	28 (1.8)	27 (1.7)	31 (2.0)	34 (2.2)	1566 (100.0)
	**80-199**	119 (76.8)	10 (6.5)	7 (4.5)	5 (3.2)	6 (3.9)	8 (5.2)	155 (100.0)
	**200-399**	36 (58.1)	8 (12.9)	2 (3.2)	4 (6.5)	9 (14.5)	3 (4.8)	62 (100.0)
	**400-999**	22 (36.1)	4 (6.6)	9 (14.8)	6 (9.8)	9 (14.8)	11 (18.0)	61 (100.0)
	**1000-4999**	24 (21.8)	1 (0.9)	4 (3.6)	9 (8.2)	29 (26.4)	43 (39.1)	110 (100.0)
	**≥5000**	13 (4.3)	9 (3.0)	4 (1.3)	8 (2.7)	36 (12.0)	231 (76.7)	301 (100.0)

(b) Probability of confirmed VL measurement resuppressing < various thresholds

		Subsequent viral load (copies/ml)						
		**<80**	**80-199**	**200-399**	**400-999**	**1000-4999**	**≥5000**	**Total**
**Confirmed viral load (copies/ml)**	**Confirmed ≥80 but not confirmed ≥200**	18 (36.7)	5 (10.2)	4 (8.2)	5 (10.2)	7 (14.3)	10 (20.4)	49 (100.0)
	**Confirmed ≥200 but not confirmed ≥400**	9 (36.0)	1 (4.0)	2 (8.0)	2 (8.0)	8 (32.0)	3 (12.0)	25 (100.0)
	**Confirmed ≥400 but not confirmed ≥1000**	1 (3.0)	1 (3.0)	3 (9.1)	5 (15.2)	8 (24.2)	15 (45.5)	33 (100.0)
	**Confirmed ≥1000 but not confirmed ≥5000**	3 (3.4)	2 (2.3)	5 (5.7)	5 (5.7)	26 (29.5)	47 (53.4)	88 (100.0)
	**Confirmed ≥5000**	0 (0.0)	4 (2.2)	1 (0.5)	1 (0.5)	17 (9.3)	159 (87.4)	182 (100.0)

e.g. confirmed ≥80 copies/ml but not confirmed ≥200 copies/ml: ≥1 of the measurements <200 copies/ml

Fig A1. Overall predicted NRTI and NNRTI susceptibility in 3NRTI (Arm C) with VL>1000 copies/ml after median 4 years on ART by CD4 monitoring strategy (n=108)

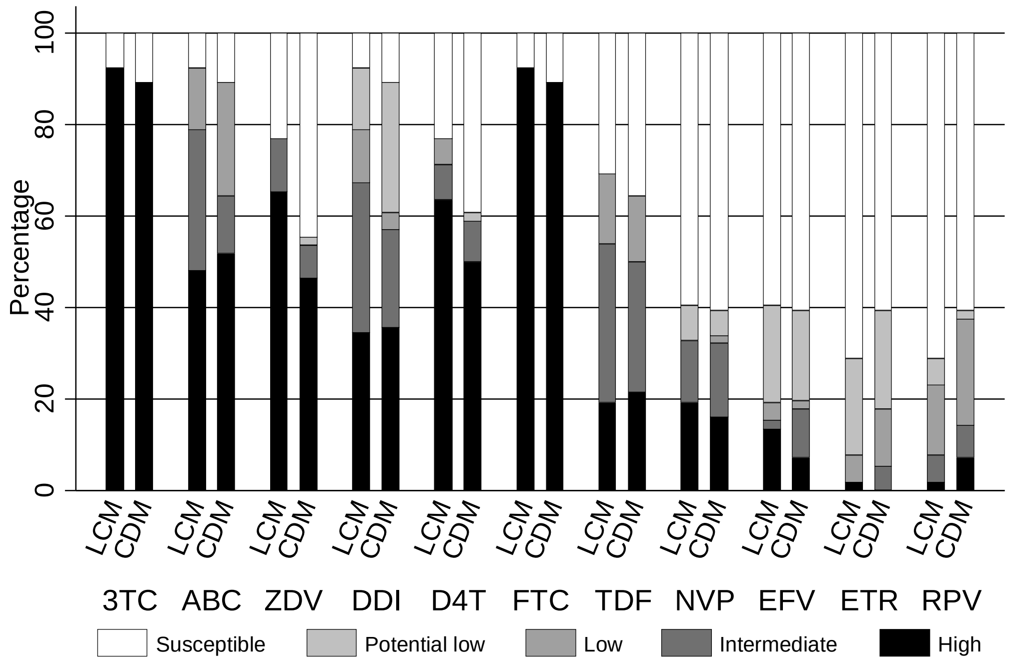

3TC=lamivudine, ABC=abacavir, ZDV=zidovudine, DDI=didanosine, D4T=stavudine, FTC=emtricitabine, TDF=tenofovir, NVP=nevirapine, EFV=efavirenz, ETR=etravirine, RPV=rilpivirine

LCM=CD4 monitoring, CDM=no CD4 monitoring

Note: impact of 2NRTI+NNRTI on predicted drug susceptibility shown in main Fig 5.

Fig A2. Prevalence of major IAS drug resistance mutations in triple NRTI maintenance (Arm-C) with VL>1000 copies/ml after median 4 years on ART

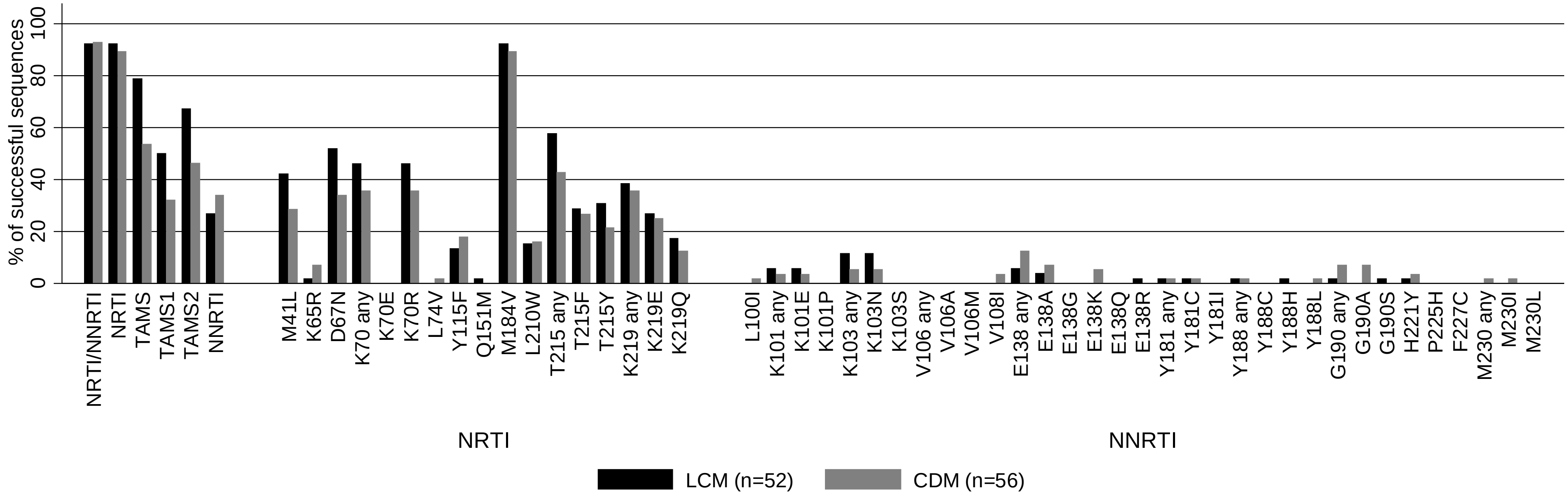

LCM vs CDM:
TAMS: p=0.008; TAMS1: p=0.08; TAMS2: p=0.03; D67N: p=0.08. All others p>0.1

LCM=CD4 monitoring, CDM=no CD4 monitoring

Fig A3. Viral load response in 107 children randomised to 3NRTI initially responding to ART

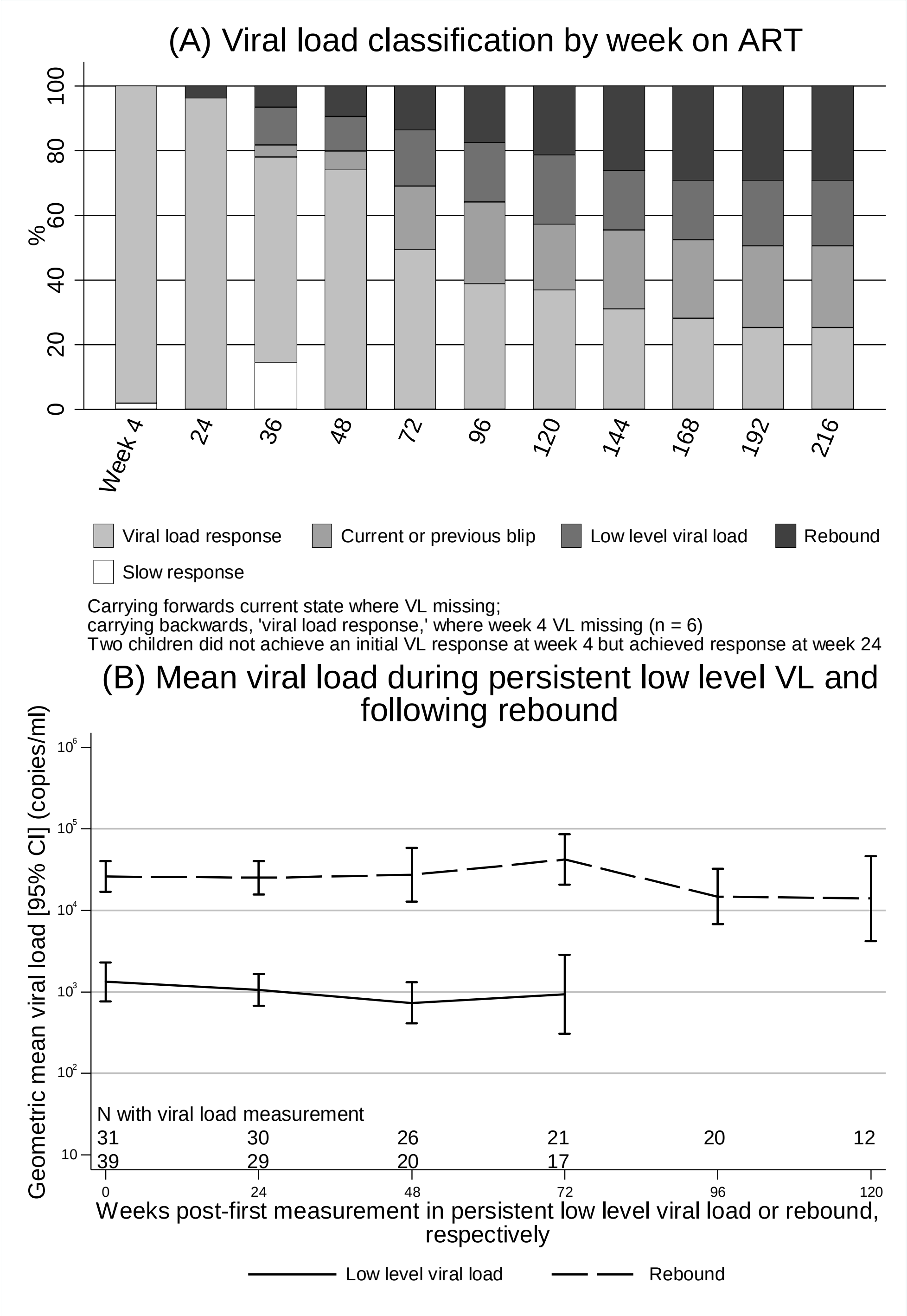

Supplement: S1 Appendix — (PDF) [file pmed.1002432.s008.pdf]
