# Supplementary material for: Virological response and resistance among HIV-infected children receiving long-term antiretroviral therapy without virological monitoring in Uganda and Zimbabwe: Observational analyses within the randomised ARROW trial
Source: PLoS Med. 2017 Nov 14;14(11):e1002432. doi: 10.1371/journal.pmed.1002432 (PMC5685482; doi:10.1371/journal.pmed.1002432)
Supplement: S1 Protocol — (PDF) [file pmed.1002432.s009.pdf]

<u>A</u>nti<u>R</u>etroviral <u>R</u>esearch f<u>O</u>r <u>W</u>atoto

ACRONYM: ARROW

Full title of trial:

A randomised trial of monitoring practice and induction maintenance drug regimens in the management of antiretroviral therapy in children with HIV infection in Africa.

ISRCTN24791884

Protocol number 2.1

Protocol date 14 March 2010

....28/5/2010

Authorised signature Date

Name: Professor Ian Weller

(Chair of Trial Steering Committee for final protocols and amended final protocols)

ARROW TRIAL

Name of person authorised to sign final protocol and protocol amendments for the sponsor (Chair of Trial Steering Committee)

Professor Ian Weller
Department of Sexually Transmitted Diseases
Mortimer Market Centre
off Capper Street
London WC1E 6AU

iweller@gum.ucl.ac.uk
Tel: +44 20 7380 9660
Fax: +44 20 7380 9669

Principal Investigators in bold and Investigators responsible for the conduct of the ARROW Trial

Dr Paula Munderi
Dr Patricia Nahirya-Ntege
MRC /UVRI Uganda Research Unit on AIDS
P. O. Box 49
Entebbe, Uganda

paula.munderi@mrcuganda.org
Patricia.Nahirya@mrcuganda.org
Tel: + 256 41 321 461
Fax: + 256 41 321137

Professor Peter Mugyenyi
Dr Victor Musiime
Joint Clinical Research Centre
PO Box 10005
Kampala, Uganda

pmugyenyi@jcrc.co.ug
musiimev@yahoo.co.uk
Tel: + 256 414 270622/283 / 273515
Fax: + 256 414 342632

Professor Addy Kekitiinwa
Dr Philippa Musoke
Dr Sabrina Bakeera-Kitaka
Baylor College of Medicine Children's Foundation
Block 5, Mulago Hospital
PO Box 72052
Kampala, Uganda

akekitiinwa@baylor-uganda.org
PMusoke@mujhu.org
skitaka@baylor-uganda.org
Tel: + 256 41 7119101 / 112 / 200
Fax: +256 41 7119116

Professor Kusum Nathoo
Dr Mutsawashe F. Bwakura-Dangarembizi
Department of Paediatrics and Child Health
University of Zimbabwe Medical School
Box A178 Avondale
Harare, Zimbabwe

knathoo@medsch.uz.ac.zw
knathoo@mweb.co.zw
dangas@zol.co.zw
mbwakura@medsch.uz.ac.zw
Tel: + 263 4 791631 ext 2224
Fax: + 263 4 700877/791995 /724912

Professor Diana Gibb
Professor Abdel Babiker
MRC Clinical Trials Unit
222 Euston Road
London NW1 2DA

d.gibb@ctu.mrc.ac.uk
a.babiker@ctu.mrc.ac.uk
Tel: +44 20 7670 4714
Fax: +44 20 7670 4815

Trial manager and monitor

Dr Margaret Thomason
MRC Clinical Trials Unit
222 Euston Road
London NW1 2DA
m.thomason@ctu.mrc.ac.uk
Tel: +44 20 7670 4615
Fax: +44 20 7670 4814/8

The trial will be conducted in compliance with the protocol, GCP and regulatory requirements.

Trial statistician

Dr Sarah Walker **s.walker@ctu.mrc.ac.uk**
Mr Adrian Cook a.cook@ctu.mrc.ac.uk
MRC Clinical Trials Unit
222 Euston Road
London NW1 2DA
Tel: +44 20 7670 4726
Fax: +44 20 7670 4818

Development of AntiRetroviral Therapy in Africa (DART) Trial collaborators

Professor Heiner Grosskurth heiner.grosskurth@mrcuganda.org
Dr Francis Ssali franssali@jcrc.co.ug
Professor James Hakim jhakim@mweb.co.zw
Dr Andrew Reid reid@uzcrc.co.zw
Professor Charles Gilks gilksc@who.int
Professor Janet Darbyshire j.darbyshire@ctu.mrc.ac.uk

Trial Funders

The trial is funded by the Medical Research Council (MRC UK), and Department for International Development, UK (DFID) and drugs are supplied by GlaxoSmithKline Ltd.

Changes from Protocol version 2.0

Minor Changes

Contact information, updated contact number for Professor Peter Mugyenyi, updated address, contact number and email for Professor Addy Kekitiinwa and her team at Baylor.

Section 1.3 (flowsheets), Section 7.4 - add storage of any blood that would normally be discarded, (as plasma) taken from sick children for diagnostic purposes (no additional blood draw). Add quantiferon storage at time of TB diagnosis and 3 months after diagnosis

Section 1.3 (flowsheets, Immunology substudy), Section 7.4 - add Quantiferon storage at weeks 120, 168, 216 (48 weekly after week 72) in the Immunology substudy in Uganda and Zimbabwe. Clarify that PBMC storage continues 48 weekly (at weeks 192, 240) after week 144 (but at different timepoints to Quantiferon so that blood draw does not increase.)

Section 4.1 and 4.2 - additional secondary endpoints "new WHO 3 or 4 event or death" and "new or recurrent WHO 3 or 4 event or death". Clarification that clinical endpoints will be evaluated including and excluding oral/oesophageal candida (invariably non-fatal). CD4 is now primary endpoint for the comparison of first-line ART strategies.

Sections 4.1.3, 5.1.3, 5.1.4, 6.4.3: revision of accrual for randomisation to once versus vs twice daily lamivudine+abacavir from 1000 to 630 (see below).

Section 7.4 Add Quantiferon storage at time of TB diagnosis and 3 months after diagnosis.

Section 7.7 Consent for withdrawal from the trial will be obtained (see Appendix 8.0)

Section 10.1 - revision of non-inferiority margin for the primary endpoint comparing LCM vs CDM given the far lower event rate observed than expected. Additional pre-specification of non-inferiority margin for the secondary endpoint "new WHO 3 or 4 event or death".

Section 10.3 - revision of non-inferiority margin for the primary endpoint comparing once versus vs twice daily lamivudine+abacavir given lower accrual as more children were ineligible due to already being on once daily than projected.

Appendix 2.0 - August 2009 clarifications from the Division of AIDS for grading the severity of adverse events added

Appendix 8.0 – Addition of the trial consent withdrawal form

Changes from Protocol version 1.1

Major Changes

Section 1.1 – Addition of third randomisation in ARROW of once daily versus twice daily and fourth randomisation to stop or continue cotrimoxazole

Section 1.2 – Addition of a flow diagram depicting the third and fourth randomisations

Section 1.3 (flowsheets) – change from 4 to 6 weekly nurse visits after 48 weeks. Addition of 96 week sample for PBMC storage (using existing blood draw at this scheduled visit)

Section 2.3.5 – Rationale for change from 4 to 6 weekly nurse visits after 48 weeks. Background to daily cotrimoxazole prophylaxis on ART and once versus twice daily Lamivudine+Abacavir

Section 3.0 – Addition of objectives for the third and fourth randomisations

Section 4.1.3, 4.1.4, 4.2 - Addition of primary and secondary endpoints for the third and fourth randomisations

Section 5.1.3, 5.1.4 - Design of the third and fourth randomisations

Section 6.4 – Addition of the inclusion and exclusion criteria, as well as patient population for the third and fourth randomisations

Section 7.3.1, 7.3.6, 7.3.7 – Details of the new randomisations, lists and registers

Section 7.4 – Conducting thick and thin films on children with suggestive symptoms on malaria

Section 7.7 – Update to the withdrawal from allocated strategy

Section 9.4, 9.4.1 – DMC monitoring of the third and fourth randomisations. Formal primary endpoint analysis at 48 weeks post randomisation into once daily versus twice daily

Section 10.3, 10.4 – Addition of the statistics for the third and fourth randomisations

Minor Changes

Section 7.4, 7.6 – Assessment of adherence to cotrimoxazole

Section 14.0 – Addition of new references, #47 up to #65

Appendix 6.7, 6.7.1, 6.7.2, 6.7.3 – Addition of patient information sheet for carers and children, and consent and assent forms, for the randomisation to stop or continue cotrimoxazole.

Appendix 6.8, 6.8.1, 6.8.2, 6.8.3 – Addition of patient information sheet for carers and children, and consent and assent forms, for the randomisation of once daily versus twice daily Lamivudine+Abacavir

Changes from Protocol version 1.0

Major Changes

Sections 1.1, 1.2, 2.3.2, 6.1, 7.3.1 - Inclusion of children aged 3 - 6 months without perinatal exposure to nevirapine (either for PMTCT or through breast-feeding)

Sections 1.3 (flowsheets), 7.1, 7.2, 7.4, Appendix 4 - Quantiferon for all children; storage of PBMCs and immunophenotyping for children enrolled into immunology substudy in Uganda. N.B. The total blood draw does not exceed the 8-10 mls in protocol version 1.0.

Section 6.2 - Exclusion of children with neutrophil count at screening $<0.5 \times 10^9$ /L (instead of $<0.75 \times 10^9$ /L) due to use of revised neutrophil toxicity gradings (see Appendix 2).

Appendix 2 - Use of WHO (2007) rather than DAIDS (2004) gradings for neutrophil toxicity to recognise that infants and children of African origin have a lower normal range of absolute neutrophil counts.

Appendix 3 - Use of modified CDC rather than WHO definitions for failure to thrive.

Minor Changes

Sections 1.3 (flowsheets), 7.4 - Addition of waist and hip measurements for all children at nurse visits, age for measurement of head circumference raised from under 2 to under 5 years of age

Section 7.8.1 - Removal of reporting of pregnancy on SAE form

Appendix 6 - Inclusion of more information on the risks and benefits of participating in ARROW in the template Patient Information Sheet

Appendix 6 - Addition of Assent form for children already enrolled who reach age 13 years

Appendix 7 - Amendments to Adherence CRFs

TABLE OF CONTENTS

1.0 Summary 12
1.1 Abstract 12
1.2 Trial Schema.. 14
1.3 Flow Sheets 16

2.0 Background and Rationale..23
2.1 Introduction 23
2.2 Ethical and practical issues.. 23
2.3 Scientific reasons 24
2.3.1 Scientific rationale for a paediatric trial 24
2.3.2 Inclusion of Infants under 6months of age 25
2.3.3 Monitoring Strategies: differences between adults and children.. 25
2.3.4 First line ART regimens 25
2.3.5 Simplification of long-term ART.. 28

3.0 Objectives 31

4.0 Endpoints..32
4.1 Primary Endpoints: 32
4.2 Secondary Endpoints: 32

5.0 Design 33
5.1 Type of design 33
5.1.1 Randomisation at enrolment to CDM or LCM arm.. 33
5.1.2 Randomisation to at enrolment ART strategies for first-line therapy 33
5.1.3 Randomisation after at least 36 weeks on ART to once or twice daily lamivudine+abacavir.. 34
5.1.4 Randomisation after at least 96 weeks of ART to continue or stop daily cotrimoxazole prophylaxis 34
5.2 Antiretroviral Therapy 34

6.0 Patient Population..34
6.1 Inclusion Criteria at ARROW enrolment.. 34
6.2 Exclusion criteria at ARROW enrolment.. 35
6.3 Number and source of subjects for ARROW enrolment 36
6.4 Once or twice daily lamivudine+abacavir randomisation.. 36
6.4.1 Inclusion criteria 36
6.4.2 Exclusion criteria 36
6.4.3 Number and source of subjects 37
6.5 Stop or continue cotrimoxazole prophylaxis randomisation.. 37
6.5.1 Inclusion criteria 37
6.5.2 Exclusion criteria 37
6.5.3 Number and source of subjects 37

7.0 Procedures and Management of Subjects..37
7.1 Screening Procedure pre ARROW enrolment.. 37
7.2 Baseline procedure (Week 0) 38
7.3 Randomisation and Enrolment 38
7.3.1 Randomisation List 38
7.3.2 First Randomisation to CDM or LCM 39
7.3.3 Second Randomisation to ART strategies for first-line therapy.. 39

7.3.4 Pregnancy 39
7.3.5 Enrolment of siblings into the trial 39
7.3.6 Randomisation to once or twice daily lamivudine+abacavir 39
7.3.7 Randomisation to stop or continue daily cotrimoxazole prophylaxis 40
7.4 Follow-up Evaluations 41
7.5 Changing ART 43
7.5.1 Substituting for Toxicity 43
7.5.2 Switching for failure of first-line therapy 43
7.6 Assessment of adherence 44
7.7 Withdrawal from allocated strategy 44
7.8 Recording and reporting of Adverse Events, Death, HIV progression and protocol violations 44
7.8.1 Serious Adverse Events (SAE), Serious Adverse Reactions (SAR) and Suspected Unexpected Serious Adverse Reactions (SUSAR): 44
7.8.2 Procedures for reporting of SAE, SAR and SUSAR: 45

8.0 Drug Regimens 47

8.1 Antiretroviral Therapy 47
8.1.1 First-line antiretroviral drugs 47
8.1.2 Second-line antiretroviral drugs 47
8.1.3 Provision of trial drugs 47
8.1.4 Modification of therapy for toxicity 48
8.1.5 Management of potential ABC or NVP reactions when children are taking both 49
8.2 Prophylaxis against Opportunistic Infections 50
8.3 Medications not permitted/ Precautions 50
8.4 Data on concomitant medications 50

9.0 Management of the Trial 50

9.1 Trial Steering Committee 50
9.2 Trial Management Group 50
9.3 Data Management and Monitoring 51
9.4 Data Monitoring Committee 51
9.4.1 Formal primary endpoint analysis for once versus twice daily lamivudine+abacavir 51
9.5 Endpoint Review Committee 52

10.0 Statistics 52

10.1 Monitoring Practice: LCM versus CDM 52
10.2 ART strategies for first line therapy 54
10.2.1 CD4 54
10.2.2 Clinical 54
10.2 Analysis Plan 54
10.3 Once versus twice daily lamivudine+abacavir 54
10.4 Stop versus continue cotrimoxazole prophlyaxis 55

11.0 Regulatory/ Ethics Approval 55

12.0 Confidentiality 56

13.0 Publication 56

14.0 References 56

APPENDICES

Appendix 1.0 Details of Antiretroviral Therapy 61

Appendix 2.0 Toxicity Gradings and Management 64

2.1 Table of Toxicity Gradings 64

2.2 Table of Clinical Signs, Symptoms, Monitoring and Management of Symptoms of Serious Adverse Effects of Antiretroviral Drugs that Require Drug Discontinuation (adapted from WHO guidelines Annex 11B).. 78

Appendix 3.0 Clinical Progression and WHO Definitions...........................80

3.1 Paediatric WHO clinical staging for HIV/AIDS for infants and children with established HIV infection 80

3.2 Presumptive and definitive criteria for recognizing HIV/AIDS-related clinical events in infants and children with established HIV infection 81

Appendix 4.0 Handling and Storage of Specimens 84

4.1 Bloods 85

4.1.1 Processing of EDTA blood for plasma and cell store 85

4.1.2 Storage of samples 85

4.1.3 Immunophenotyping 70

4.1.4 Collection of supernatants using the QuantiFERON®-TB Gold In-Tube (IT) System.. 70

4.1.5 Storage of Peripheral Blood Mononuclear Cells (PBMCs) 70

Appendix 5.0 Management of Safety 87

5.1 Abacavir Hypersensitivity... 87

5.1.1 Description of the Hypersensitivity Reaction.. 87

5.1.2 Management of Hypersensitivity Reactions.. 88

5.1.3 Special considerations following an interruption of abacavir therapy......... 88

5.1.4 Essential patient information 88

5.1.5 Reporting of Hypersensitivity Reactions... 89

5.1.6 Stevens Johnson Syndrome, Toxic Epidermal Necrolysis or Erythema Multiforme 89

5.1.7 Management of Rash that is Not Accompanied by Systemic Symptoms 89

5.2 Haematological Disorders with zidovudine 90

5.3 Nucleoside Reverse Transcriptase Inhibitors, including abacavir, lamivudine and zidovudine.. 90

5.3.1 Lactic Acidosis/Severe Hepatomegaly with Steatosis 90

5.3.2 Pancreatitis.. 90

5.3.3 Immune Reconstitution Syndrome.. 91

Appendix 6.0 Patient Information Sheets and Consent Forms (Templates)92

6.1 Information sheet for carers 92

6.2 Information sheet for children 97

6.3 Information about abacavir for carers 100

6.4 Screening consent form... 101

6.5 Trial consent form.. 102

6.6 Trial Assent form.. 97

6.7 Stop or Continue cotrimoxazole randomisation 98

6.7.1 Information sheet for carers.. 98

6.7.2 Information sheet for children 100
6.7.3 Stop or Continue Cotrimoxazole consent form 102
6.7.4 Stop or Continue Cotrimoxazole assent form.. 103
6.8 Once versus twice daily lamivudine+abacavir randomisation 104
6.8.1 Information sheet for carers.. 104
6.8.2 Information sheet for children 106
6.8.3 Once daily versus twice daily randomisation consent form................................ 108
6.8.4. Once daily versus twice daily randomisation assent form 109

Appendix 7.0 Adherence Questionnaires..116

7.1 Adherence questionnaire for carers 116
7.2 Adherence questionnaire for children/adolescents 118

Appendix 8.0 Trial consent withdrawal form 120

Glossary and Abbreviations

**3TC**	Lamivudine
**ABC**	Abacavir
**AE**	Adverse event
**ARROW**	AntiRetroviral Research fOr Watoto
**ART**	Antiretroviral Therapy
**AST**	Aspartate aminotransferase
**ALT**	Alanine aminotransferase
**CDC**	Centers for Disease Control
**CDM**	Clinically Driven Monitoring
**CRF**	Case Report Form
**D4T**	Stavudine
**DART**	Development of AntiRetroviral Therapy
**DFID**	Department for International Development (UK)
**ddI**	Didanosine
**DMC**	Data Monitoring Committee
**EFV**	Efavirenz
**ELISA**	Enzyme-Linked Immunosorbent Assay
**FACS**	Fluorescent Activated Cell Sorting
**GCP**	Good Clinical Practice
**GSK**	Glaxo Smith Kline Ltd.
**Hb**	Haemogolobin
**HLA**	Human Leukocyte Antigen
**JCRC**	Joint Clinical Research Centre, Kampala, Uganda
**KAL**	Kaletra
**LCM**	Laboratory and Clinical Monitoring
**MCV**	Mean cell volume
**MOP**	Manual of Operations
**MRC CTU**	Medical Research Council Clinical Trials Unit
**MRC UVRI**	Medical Research Council Uganda Virus Research Institute
**MTCT**	Mother-To-Child Transmission
**NRTI**	Nucleoside Reverse Transcriptase Inhibitor
**NNRTI**	Non-Nucleoside Reverse Transcriptase Inhibitor
**NVP**	Nevirapine
**OI**	Opportunistic Infection
**PBMC**	Peripheral Blood Mononuclear Cell
**PCR**	Polymerase Chain Reaction
**PEPFAR**	The President's Emergency Plan for AIDS Relief
**PI**	Protease Inhibitor
**PIDC**	Paediatric Infectious Disease Clinic
**PMTCT**	Prevention of mother to child transmission
**RNA**	Ribonucleic acid
**SAE**	Serious adverse event
**TDF**	Tenofovir
**TRECS**	T-cell receptor excision circles
**TSC**	Trial Steering Committee
**UNAIDS**	The Joint United Nations Programme on HIV/AIDS
**UZ CRC**	University of Zimbabwe Clinical Research Centre
**WBC**	White blood cells
**WHO**	World Health Organisation
**ZDV**	Zidovudine

1.0 Summary

1.1 Abstract

The ARROW (AntiRetroviral Research fOr Watoto) protocol describes an open-label randomised trial primarily evaluating two strategic approaches for management of antiretroviral therapy (ART) in 1200 symptomatic HIV infected infants and children in Uganda and Zimbabwe. The first strategy compares clinically driven monitoring (CDM) with laboratory plus clinical monitoring (LCM). The second approach compares a *continuous* first line ART three drug two class regimen, comprising two Nucleoside Reverse Transcriptase Inhibitors (NRTIs) plus one Non-Nucleoside Reverse Transcriptase Inhibitor (NNRTI), with *induction* with four drugs (two classes) followed by *maintenance* with three drugs. After at least 36 and 96 weeks on ART respectively, two further randomisations will assess simplification strategies which could improve long-term ART adherence (i) once versus twice daily lamivudine+abacavir NRTI drugs (ii) stopping versus continuing daily cotrimoxazole prophylaxis.

Eligible patients will be HIV infected children whose adult carers have appropriate access to ART; either enrolled in the adult DART (Development of AntiRetroviral Therapy) trial, on ART through another programme, or who do not require ART (e.g. if not HIV infected). At enrolment, trial entrants will be aged 3 months to 17 years (13 to 17 years to be capped at 10%),,must have no clinical or laboratory abnormalities contra-indicating the start of ART and meet WHO guidelines for starting antiretroviral treatment (see section 6.1). Infants aged 3 to 6 months who have been exposed to nevirapine during the perinatal period, either through prevention of mother to child transmission (PMTCT) or breast-feeding, will be excluded from the trial.

A total of 1200 children from 4 African sites (3 in Uganda, 1 in Zimbabwe) will be enrolled over 12-18 months and followed for 3½-5 years. In both CDM and LCM arms, children will have haematology, and liver function tests performed routinely but the results will only be returned to clinicians caring for children in the CDM arm if requested by the treating physician for clinical reasons, or if indicative of a grade 4 adverse event. The exception will be return of haemoglobin results on all children at week 8. CD4 counts will be performed both in CDM and LCM arms with results not returned to physicians for patients in the CDM arm but monitored independently by the Data Monitoring Committee (DMC).

The second randomisation, to ART strategies for first-line therapy, will also take place at enrolment. All children will receive two NRTIs - abacavir (ABC) and lamivudine (3TC) - plus an NNRTI (either efavirenz [EFV] or nevirapine [NVP]). The control arm (arm A) will continue with this 3-drug ART regimen for the duration of first-line therapy. The induction maintenance arms (Arms B and C) will receive a fourth drug, the NRTI zidovudine (ZDV), for the first 36 weeks. After 36 weeks, children in Arm B will stop taking ZDV and children in Arm C will stop taking NNRTI (i.e. both arms will reduce to 3 drugs) for the remainder of their first-line ART.

The third randomisation, to once daily versus continuation of twice daily lamivudine+abacavir, will take place after children have been on ART for at least 36 weeks. The fourth randomisation, to stop or continue daily cotrimoxazole prophylaxis, will take place after children (currently aged at least 3 years) have been on ART for at least 96 weeks. All children living in malaria endemic areas will have access to insecticide treated bednets.

The primary efficacy endpoint for LCM/CDM and induction-maintenance ART strategies will be progression to a new paediatric WHO HIV stage 4 disease event or death. The

decision to change to second-line ART will be based on clinical criteria alone for the CDM arm and on clinical plus CD4 criteria for the LCM arm. Second-line ART is likely to include a boosted PI with either NRTIs or an NNRTI, depending on the first-line regimen received, and will be provided to children failing within the timeframe of the trial. Children who fail the second-line regimen will be offered the best combination available from the first and second line drugs. The primary endpoints for the once versus twice daily lamivudine+abacavir randomisation are HIV RNA viral load (measured retrospectively at 48 weeks post randomisation) and adherence to ART at 96 weeks. For the cotrimoxazole prophylaxis randomisation, the primary endpoints are hospitalisations or death, and adherence to ART.

ARROW is a five-year trial. Recruitment will take place over 18 months with follow-up of 3½ years after the last patient is randomised.

1.2 Trial Schema

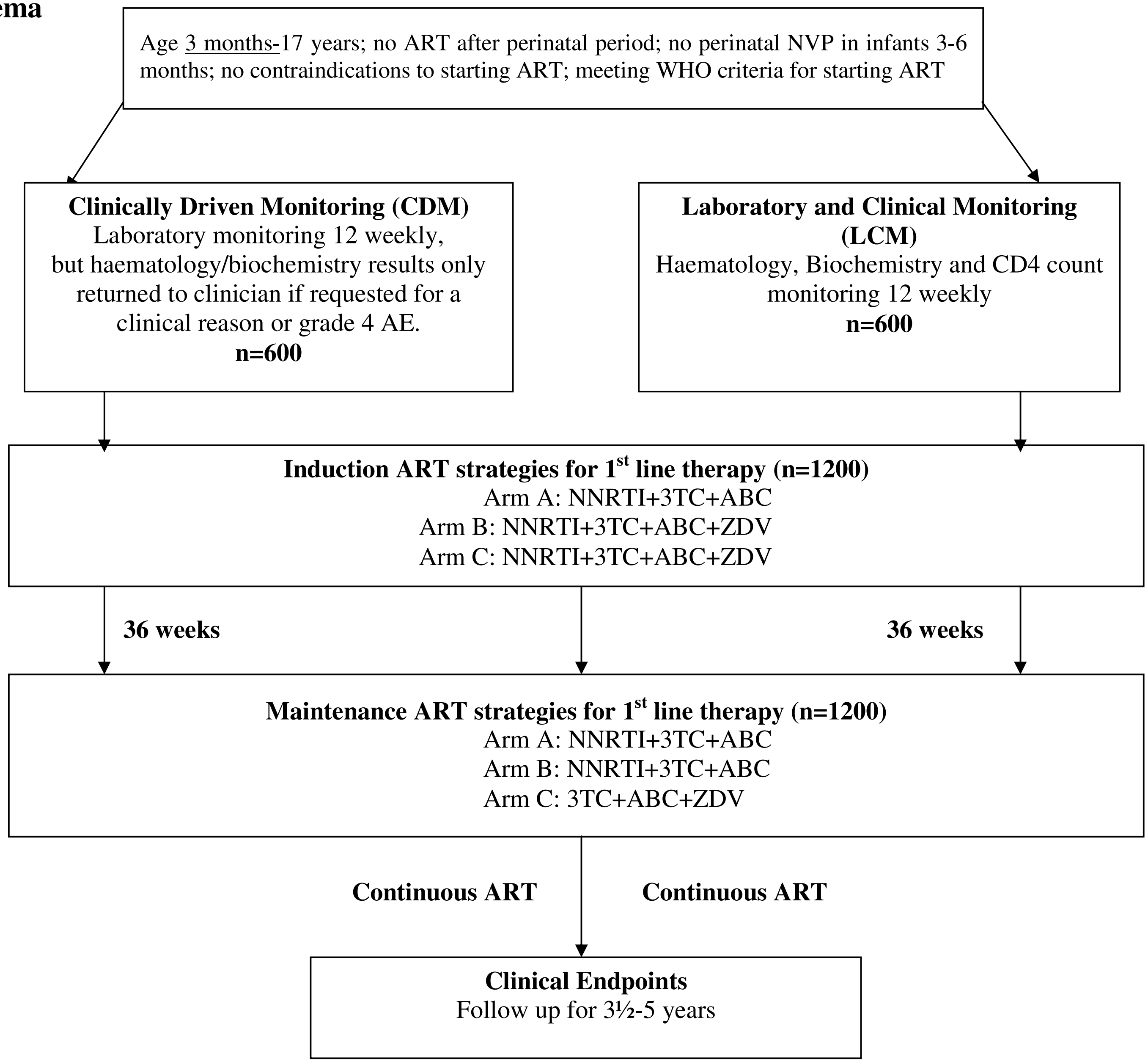

1.2.1 *Secondary randomisations: simplification of long-term ART*

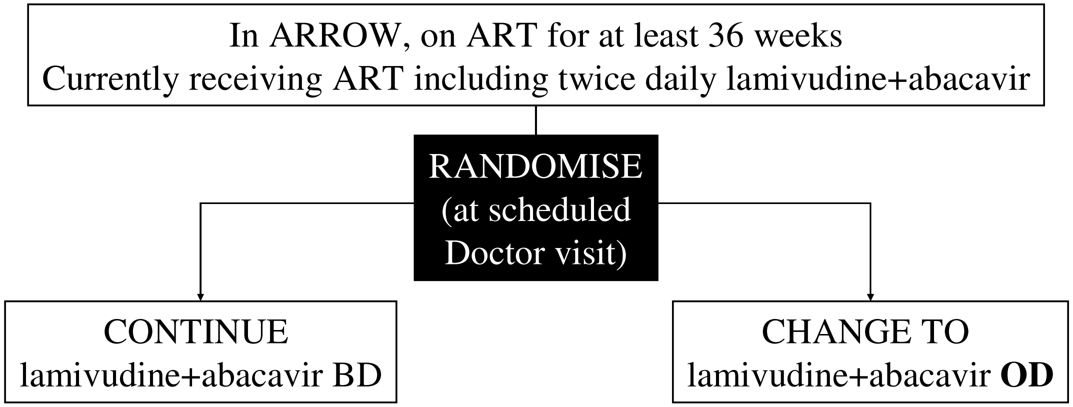

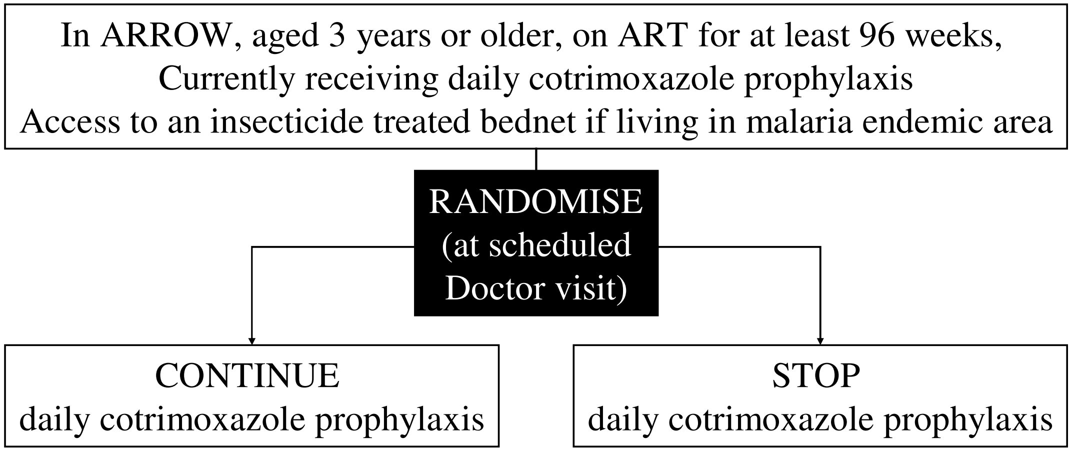

Note: no difference to flowsheets in section 1.3 below, as children will undergo the standard 12 weekly Doctor follow-up investigations

1.3 Flow Sheets

1.3.1 *Children not participating in the Immunology Sub-study (flow sheet as per protocol 1.0, nurse visits 6 weekly after 48 weeks)*

EVENTS	WEEK IN TRIAL																				
Doctor/Nurse visit*<br>Nurse visit<br>Doctor only visit*	Screening Week-2	Start therapy Week 0	2	4	8	12	16	20	24	28	32	36	40	44	48	54	60	6 weekly	12 weekly	Switch[8]
4/6 weeks drug supply until next visit*		X		X	X	X	X	X	X	X	X	X	X	X	X	X	X	X	X	X
Consent for screening and patient information	X																			
Informed consent		X																		
History & Physical[1]	X	X	X	X	X	X			X			X			X		X		X	X
Symptom check list (nurse)		X	X	X	X	X	X	X	X	X	X	X	X	X	X	X	X	X	X	X
Pregnancy Test[2]	X								X						X					
Haematology[3] 1.5-2mls	X			X	X	X			X			X			X		X		X	X
Biochemistry[4] 1.5-2mls	X			X	X	X			X			X			X		X		X	X
Lymphocyte Subsets[5]	X	X				X			X			X			X		X		X	X
Plasma storage[6] 4-5mls	X	X		X		X			X			X			X		X		X	X
Adherence assessment[7]				X	X	X	X	X	X	X	X	X	X	X	X	X	X	X	X	

* Participants will return 6-weekly (4-weekly before week 48) to see the nurse or doctor, return used drug containers and receive 4/6 weeks of antiretroviral therapy (ART) until their next visit. The doctor will prescribe ART and make decisions on any modifications of therapy as necessary. Children on NVP will dose escalate at week 2.

1 Clinical: including weight, height, head circumference (if under 5 years), Tanner staging every 24 weeks (if 10 years or older), adverse events and paediatric WHO staging for HIV.

2 At screening, and subsequently every 24 weeks for girls $\geq$ 12 years who have reached menses (Tanner Stage 4)

3 Haematology: Hb, MCV, WBC, Lymphocytes, Neutrophils, Platelets. All results are **blinded in CDM group** except at screening (week –2) when all results are returned to clinician, at week 8 when Hb results are returned or when results are requested for clinical reasons (see section 7.4, page 41).

4 Biochemistry: Urea, Creatinine, AST, ALT, Bilirubin. All results are **blinded in CDM group** except at screening (week –2) when all results are returned to clinician or when results are requested for clinical reasons (see section 7.4, page 41).

5 CD3, CD4, CD8 percentage and absolute, total lymphocyte count. All results are **blinded in CDM group** except at screening (week –2) when all results are returned to clinician

6 Plasma from a total of 5ml EDTA blood. Store DNA pellet at week –2, 0, 24, 48 and then every 48 weeks (if feasible). See section 0 for instructions about storage. Take plasma (DNA pellet if feasible) at time of switch. Take plasma as near as possible to time of delivery for any women giving birth. If children become ill enough to have blood tests done outside of the routine blood sampling schedule then store any blood that would normally be discarded, as plasma for retrospective analysis (but do not take extra blood from sick children).

7 Pill count and nurse administered questionnaire

8 At time of switch to 2nd line reset flow sheet to visit week 0

1.3.2 *Children enrolled in Uganda and participating in the Immunology Sub-study*

Flow sheet 1: Up to 72 weeks (children enrolled in Uganda and participating in the Immunology Sub-study):

(Please note: No extra volumes of blood are required for the immunology sub-study, all samples taken remain within the 8-10mls as per protocol 1.0)

EVENTS	WEEK IN TRIAL																										
Doctor/Nurse visit* ☐<br>Nurse visit ☐<br>Doctor only visit* ☐	Screening Week-2	Start therapy Week 0	2	4	8	12	16	20	24	28	32	36	40	44	48	54	60	66	72	Switch to 2nd line[8]	Weeks post switch +4	+8	+12	+24	+48	6 wkly	12 wkly
2 week drug supply		X	X																								
4/6 weeks drug supply*				X	X	X	X	X	X	X	X	X	X	X	X	X	X	X	X	X	X	X	X	X	X	X	X
Consent for screening and child information	X																										
Informed consent		X																									
History & Physical[1]	X	X	X	X	X	X			X			X			X		X		X	X	X	X	X	X	X		X
Symptom check list		X	X	X	X	X	X	X	X	X	X	X	X	X	X	X	X	X	X	X	X	X	X	X	X	X	X
Pregnancy Test[2]	X								X						X				X					X			
Haematology 1.5-2mls[3]	X			X	X	X			X			X			X		X		X	X	X	X	X	X	X		X
Biochemistry 1.5mls[4]	X			X	X	X			X			X			X		X		X	X	X	X	X	X	X		X
Lymphocyte Subsets (same draw as haem)[5]	X	X				X			X			X			X		X		X	X			X	X	X		X
Plasma storage 3.5-7ml[6]	**X**	**X**		**X**		**X**			**X**			**X**			**X**		X		**X**	**X**	X		**X**	**X**	**X**		X
Adherence assessment[7]				X		X			X			X			X		X		X				X	X	X		X
Quantiferon 3 x 1mls[9]**		X		X								X							X								
PBMCs (3mls from same blood draw as for plasma storage)[10]	X					X			X						X					X			X	X	X		

Immunophenotyping (using existing 1.5 – 2mls haem/lymp)[11]	X	(X)		X		X			X			X			X				X	X			X	X	X		

* Participants will return 6-weekly (4-weekly before week 48) to see the nurse or doctor, return used drug containers and receive 4/6 weeks of antiretroviral therapy (ART) until their next clinic visit. The doctor will prescribe ART and make decisions on any modifications of therapy as necessary. Children on NVP will dose escalate at week 2.

See page below for footnotes

Flow sheet 2: Week 76 onwards (children enrolled in Uganda and participating in the Immunology Sub-study)

(Please note: No extra volumes of blood are required for the immunology sub-study, all samples taken remain within the 8-10mls as per protocol 1.0)

EVENTS	WEEK IN TRIAL																						
Doctor/Nurse visit* ■<br>Nurse visit □																Switch to 2nd line[8]	Weeks post switch						
	78	84	90	96	102	108	114	120	126	132	138	144	6 wkly	12 wkly	24 wkly		+4	+8	+12	+24	+48	6 wkly	12 wkly
4/6 weeks drug supply*	X	X	X	X	X	X	X	X	X	X	X	X	X	X	X	X	X	X	X	X	X	X	X
History & Physical[1]		X		X		X		X		X		X		X	X	X	X	X	X	X	X		X
Symptom check list (nurse)	X	X	X	X	X	X	X	X	X	X	X	X	X	X	X	X	X	X	X	X	X	X	X
Pregnancy Test[2]				X				X				X			X					X			
Haematology 1.5-2mls [3]		X		X		X		X		X		X		X	X	X	X	X	X	X	X		X
Biochemistry 1.5mls [4]		X		X		X		X		X		X		X	X	X	X	X	X	X	X		X
Lymphocyte Subsets (same draw as haem)[5]		X		X		X		X		X		X		X	X	X			X	X	X		X
Plasma storage 3.5-7mls [6]		X		**X**		X		X		X		**X**		X	X	**X**	X		**X**	**X**	**X**		X
Adherence assessment[7]		X		X		X		X		X		X		X	X				X	X	X		X
Quantiferon 3 x 1mls [9 **]								X															
PBMCs (3mls from same blood draw as for plasma storage) [10]				X								X				X			X	X	X		
Immunophenotyping (using existing 1.5 – 2mls haem/lymp)[11]				X				X				X			X	X			X	X	X		

- Participants will return 6-weekly to see the nurse or doctor, return used drug containers and receive 4/6 weeks of antiretroviral therapy (ART) until their next clinic visit. The doctor will prescribe ART and make decisions on any modifications of therapy as necessary.

See page below for footnotes

[1] Clinical: including weight, height, head circumference (if under 5 years), Mid-upper arm circumference, waist measurement, hip measurement, Tanner staging every 24 weeks (if 10 years or older), adverse events and paediatric WHO staging for HIV.

[2] At screening, and subsequently every 24 weeks for girls $\geq$ 12 years who have reached menses (Tanner Stage 4)

[3] Haematology: Hb, MCV, WBC, Lymphocytes, Neutrophils, Platelets. All results are **blinded in CDM group** except at screening (week –2) when all results are returned to clinician, at week 8 when Hb results are returned or when results are requested for clinical reasons (see section 7.4 of ARROW protocol) or Grade 4 toxicity.

[4] Biochemistry: Urea, Creatinine, AST, ALT, Bilirubin. All results are **blinded in CDM group** except at screening (week –2) when all results are returned to clinician or when results are requested for clinical reasons (see section 7.4 of ARROW protocol) or Grade 4 toxicity.

[5] CD3, CD4, CD8 percentage and absolute, total lymphocyte count. All results are **blinded in CDM group** except at screening (week –2) when all results are returned to clinician

[6] Plasma (EDTA blood) from a total of 3.5 - 7ml, (depending on whether Quantiferon or PBMC bloods are also drawn, samples in bold indicate only 3.5-4ml EDTA blood to be taken for plasma storage). Store DNA pellet at week –2, 0, 24, 48 and then every 48 weeks (if feasible). See Appendix 4.0 for instructions about storage. Take plasma (DNA pellet if feasible) at time of switch. Take plasma as near as possible to time of delivery for any women giving birth. If children become ill enough to have blood tests done outside of the routine blood sampling schedule then store any blood that would normally be discarded, as plasma for retrospective analysis (but do not take extra blood from sick children).

[7] Pill count and visual analogue scale every 4/6 weeks at nurse visits; nurse administered questionnaire every 12 weeks

[8] At time of switch to 2nd line reset flow sheet to visit week 0, except for samples taken for the immunology sub-study. For Immunophenotyping and PBMCs take a sample at time of switch and then only at weeks 12, 24 and 48 after switch. Do not take any further Quantiferon samples at switch or post switch

[9] Quantiferon: a total of 3mls blood drawn in three 1 ml tubes, negative control used for LPS assay. Done 48 weekly after week 72 (ie also at weeks 120, 168 and 216 for children reaching these time points).

[9 **] Quantiferon: at any time point where a child is diagnosed with TB, quantiferon is to be stored at the time of TB diagnosis and 3 months after diagnosis

[10] PBMCs: 3mls of blood from the plasma storage (EDTA blood). Done 48 weekly after week 144 (ie also at week 192 and 240 for children reaching these time points).

[11] No extra bloods are taken for immunophenotyping as this assay uses the existing haematology/lymphocyte blood draws. If immunophenotyping is not achieved at screening it will be requested at week 0, as indicated in brackets

Therefore the 8 – 10 mls of blood taken is divided as follows: Haematology/lymphocyte subsets/immunophenotyping (1.5 – 2mls); Biochemistry (1.5 mls) and the remaining 6.5 – 7mls divided between Quantiferon, PBMC and Plasma storage

1.3.3 *Children enrolled in Zimbabwe and participating in the Immunology Sub-study*

Flow sheet 1: Up to 72 weeks (children enrolled in Zimbabwe and participating in the Immunology Sub-study):

(Please note: No extra volumes of blood are required for the immunology sub-study, all samples taken remain within the 8-10mls as per protocol 1.0)

EVENTS	WEEK IN TRIAL																			
Doctor/Nurse visit* ▢<br>Nurse visit ▢<br>Doctor only visit* ■	Screening Week-2	Start therapy Week 0	2	4	8	12	16	20	24	28	32	36	40	44	48	54	60	66	72	Switch to 2nd line[8]
2 week drug supply		X	X																	
4/6 weeks drug supply*				X	X	X	X	X	X	X	X	X	X	X	X	X	X	X	X	X
Consent for screening and child information	X																			
Informed consent		X																		
History & Physical[1]	X	X	X	X	X	X			X			X			X		X		X	X
Symptom check list (nurse)		X	X	X	X	X	X	X	X	X	X	X	X	X	X	X	X	X	X	X
Pregnancy Test[2]	X								X						X				X	
Haematology 1.5-2mls [3]	X			X	X	X			X			X			X		X		X	X
Biochemistry 1.5mls [4]	X			X	X	X			X			X			X		X		X	X
Lymphocyte Subsets (same draw as haem)[5]	X	X				X			X			X			X		X		X	X
Plasma storage 3.5-7mls [6]	X	**X**		**X**		X			X			**X**			X		X		**X**	X
Adherence assessment[7]				X		X			X			X			X		X		X	
Quantiferon 3 x 1mls [9] **		X		X								X							X	

* Participants will return 6-weekly (4-weekly before week 48) to see the nurse or doctor, return used drug containers and receive 4/6 weeks of antiretroviral therapy (ART) until their next clinic visit. The doctor will prescribe ART and make decisions on any modifications of therapy as necessary. Children on NVP will dose escalate at week 2.

See page below for footnotes

Flow sheet 2: Week 76 onwards (children enrolled in Zimbabwe and participating in the Immunology Sub-study)

(Please note: No extra volumes of blood are required for the immunology sub-study, all samples taken remain within the 8-10mls as per protocol 1.0)

EVENTS	WEEK IN TRIAL															
Doctor/Nurse visit* ■<br>Nurse visit □	78	84	90	96	102	108	114	120	126	132	138	144	6 weekly	12 weekly	24 weekly	Switch to 2nd line[8]
4/6 weeks drug supply*	X	X	X	X	X	X	X	X	X	X	X	X	X	X	X	X
History & Physical[1]		X		X		X		X		X		X		X	X	X
Symptom check list (nurse)	X	X	X	X	X	X	X	X	X	X	X	X	X	X	X	X
Pregnancy Test[2]				X				X				X			X	
Haematology 1.5-2mls [3]		X		X		X		X		X		X		X	X	X
Biochemistry 1.5mls [4]		X		X		X		X		X		X		X	X	X
Lymphocyte Subsets (same draw as haem)[5]		X		X		X		X		X		X		X	X	X
Plasma storage 3.5-7mls [6]		X		X		X		X		X		X		X	X	X
Adherence assessment[7]		X		X		X		X		X		X		X	X	

* Participants will return 6-weekly (4-weekly before week 48) to see the nurse or doctor, return used drug containers and receive 4/6 weeks of antiretroviral therapy (ART) until their next clinic visit. The doctor will prescribe ART and make decisions on any modifications of therapy as necessary.

1 Clinical: including weight, height, head circumference (if under 5 years), Mid-upper arm circumference, waist measurement, hip measurement, Tanner staging every 24 weeks (if 10 years or older), adverse events and paediatric WHO staging for HIV.

2 At screening, and subsequently every 24 weeks for girls $\geq$ 12 years who have reached menses (Tanner Stage 4)

3 Haematology: Hb, MCV, WBC, Lymphocytes, Neutrophils, Platelets. All results are **blinded in CDM group** except at screening (week –2) when all results are returned to clinician, at week 8 when Hb results are returned or when results are requested for clinical reasons (see section 7.4) or Grade 4 toxicity.

4 Biochemistry: Urea, Creatinine, AST, ALT, Bilirubin. All results are **blinded in CDM group** except at screening (week –2) when all results are returned to clinician or when results are requested for clinical reasons (see section 7.4) or Grade 4 toxicity.

5 CD3, CD4, CD8 percentage and absolute, total lymphocyte count. All results rare **blinded in CDM group** except at screening (week –2) when all results are returned to clinician.

6 Plasma (EDTA blood) from a total of 3.5 - 7ml, (depending on whether Quantiferon bloods are also drawn, samples in bold indicate only 3.5-4ml EDTA blood to be taken for plasma storage) Store DNA pellet at week –2, 0, 24, 48 and then every 48 weeks (if feasible). See Appendix 4.0 for instructions about storage. Take plasma (DNA pellet if feasible) at time of switch. Take plasma as near as possible to time of delivery for any women giving birth. If children become ill enough to have blood tests done outside of the routine blood sampling schedule then store any blood that would normally be discarded, as plasma for retrospective analysis (but do not take extra blood from sick children).

7 Pill count and visual analogue scale every 4/6 weeks at nurse visits; nurse administered questionnaire every 12 weeks

8 At time of switch to 2nd line reset flow sheet to visit week 0 (except for Quantiferon samples which end at week 72).

9 Quantiferon: a total of 3mls blood drawn in three 1 ml tubes, negative control used for LPS assay. At intervals up to week 72. Done 48 weekly after week 72 (ie also at weeks 120, 168 and 216 for children reaching these time points)

9 ** Quantiferon: at any time point where a child is diagnosed with TB, quantiferon is to be stored at the time of TB diagnosis and 3 months after diagnosis

Therefore the 8 – 10 mls of blood taken is divided as follows: Haematology/lymphocyte subsets (1.5 – 2mls); Biochemistry (1.5ml) and the remaining 6.5 – 7mls divided between Quantiferon and Plasma storage

2.0 Background and Rationale

2.1 Introduction

Over 95% of paediatric infections are acquired through mother-to-child transmission (MTCT), and sub-Saharan African countries have the highest HIV prevalence in pregnant women worldwide (>20% in many countries). By the end of 2003, there were an estimated 2.5 million children under 15 years living with HIV infection in the world, 87% of whom live in sub-Saharan Africa, where the epidemic has reversed the gains in child survival which occurred in the 1980s. Despite increasing availability of interventions to reduce MTCT, in Africa during 2003 alone over 600,000 children were estimated to be born with HIV infection and 500,000 died [1].

Effective triple-drug antiretroviral therapy (ART) has resulted in major reductions in mortality and morbidity in HIV-infected adults and children in industrialised countries [2,3]. Until recently, high costs were an absolute barrier to the introduction of ART in resource-limited countries. Drug access is now widening due to price reductions and new donor commitments including PEPFAR, Gates and Clinton Foundations and the Global Fund. WHO aimed to increase the number of adults and children receiving ART in resource-limited countries by 3 million by the end of 2005 (3 by 5 plan) [4]. However, the WHO report to June 2005 acknowledged that far fewer children were receiving ART than the targeted 15%, calculated to be the proportion of children among infected individuals requiring ART [5].

However, drug costs are only part of the financial burden of providing ART, and high levels of infrastructure are required to provide laboratory tests routinely used in industrialised countries [6]. The DART (Development of AntiRetroviral Therapy) trial is the first to address questions about monitoring practice in symptomatic HIV-infected adults in Uganda and Zimbabwe. The main question in DART is whether ART can be given safely with clinical monitoring alone, in the absence of regular CD4 measurements and laboratory monitoring for toxicity. The first-line drugs being used in DART include NNRTI (Non-Nucleoside Reverse Transcriptase Inhibitor) + 2 NRTI (Nucleoside Reverse Transcriptase Inhibitor) and triple NRTI regimens. The use of more than one first-line regimen increases the generalisability of results pertaining to strategies for administering ART in resource-limited settings. A written commitment has been obtained from governments to continue treating DART participants after the trial has ended and investigators are working with the health authorities in Uganda and Zimbabwe to prepare for this transition. The MRC Clinical Trials Unit, with Imperial College London, is undertaking overall co-ordination of the trial. Enrolment to DART started in 2003, and 3314 eligible patients were enrolled by October 2004 [7].

There is an urgent need to develop viable treatment strategies for children in resource-limited settings. There are scientific, ethical and practical reasons why a trial addressing the monitoring question in the DART trial should be undertaken in children. There is a unique opportunity provided by DART for a parallel trial in children, capitalising on existing infrastructure and trial expertise.

2.2 Ethical and practical issues

In DART, enrolment was offered to all infected adults in a household meeting the inclusion criteria for ethical, and also practical, reasons (to avoid pill sharing). It has become increasingly apparent that the same ethical and practical reasons for inviting all eligible adults

in a household to be enrolled in DART, also apply to the children. In an anonymous questionnaire survey of a systematic sample of 223 DART participants in October 2003, 41% of participants reported that they were caring for at least one child known or suspected to be HIV infected. At that time only 3% of suspected infected children had a positive HIV antibody test while most of the remainder had never been tested. Only three of the 648 children in these households had received ART at birth.

Until recently, testing and providing care for children with HIV-infection was not a priority in many African countries. However, following evidence in 2004 that cotrimoxazole prophylaxis reduced mortality in HIV-infected children in Zambia by 43% [8], paediatricians working with the DART trial teams in Uganda and Zimbabwe started to set up clinics offering testing, cotrimoxazole prophylaxis and nutritional support for children of DART trial participants. Children requiring immediate ART because of advanced HIV infection are being referred to appropriate facilities where drugs are available. In Uganda, PEPFAR funding has been provided in several facilities to provide ART for children. Some ART is becoming available in Zimbabwe, although donor funding is less.

2.3 Scientific reasons

In adults and children starting combination ART in well-resourced countries, monitoring of prognostic markers of HIV disease (HIV RNA viral load and CD4 cell counts) is routinely undertaken in order to make decisions about switching therapy. In addition, routine monitoring of laboratory markers is undertaken to detect bone marrow, liver and other toxicities to antiretroviral drugs. However, the costs and infrastructure required for these tests are high, and there is clearly a lack of infrastructure and financial resource for laboratory monitoring in most developing country settings [6]. There have been no studies evaluating the need for, or frequency of, laboratory tests required for monitoring ART in children, although several studies (including the DART trial) are ongoing in adults. HIV RNA tests are particularly expensive (although the cost may come down), and unavailable outside major centres in Africa. There are also important issues of quality control. Requirements for monitoring schedules similar to those in industrialised countries are likely to be a barrier to the administration of ART to large numbers of adults and children in resource-limited settings [9].

Although HIV RNA viral load is an important prognostic marker and is a widely used monitoring tool, recently greater emphasis has been placed on monitoring CD4 cell counts or percentages which are better predictors of the immediate risk of clinical disease progression and mortality, particularly in late disease [10]. Furthermore, with the current therapeutic options, it is more difficult to maintain HIV RNA below the limit of detection of current assays over a long period of time, whereas increases in CD4 count can frequently be maintained, particularly in children [11].

It is unlikely therefore that HIV-RNA viral load monitoring would be cost-effective in the African setting [6,9]. However, it is unclear whether decisions about management of ART toxicity or switching ART drugs can be made on the basis of clinical signs and symptoms alone or should be based on clinical monitoring plus regular CD4 counts and laboratory monitoring for toxicity.

2.3.1 *Scientific rationale for a paediatric trial*

Although the question of monitoring is being addressed in adult DART, it is also pertinent to children. There are good reasons for believing that the results of such a trial may be different in children, since their monitoring requirements may differ from those of adults. In addition there is uncertainly about the best first-line ART to offer children in resource limited settings.

2.3.2 *Inclusion of Infants under 6months of age*

HIV infection in infancy is characterised by rapid disease progression and high mortality [12-14]. The risk remains substantial despite high CD4% values, which are of limited prognostic value in infancy [10]. Increased availability of HIV-DNA polymerase chain technology has greatly facilitated early diagnosis of HIV in Uganda and Zimbabwe, making it appropriate to reduce the lower age limit for eligibility to ARROW. Since the safety and effectiveness of abacavir has been established in paediatric patients aged 3 months and above [15] it is proposed to extend the age of enrolment to ARROW to infants of ≥ 3 months. Exposure to nevirapine in the perinatal period is associated with increased NNRTI drug resistance mutations and poorer virological response to NNRTI-containing regimens [16] and infants aged 3-6 months who have been exposed to nevirapine in the perinatal period (either through PMTCT or breast-feeding) will be excluded from the trial. Recent randomised evidence from South Africa suggests that early initiation of ART substantially improves outcome [17]. In the Children with HIV Early Antiretroviral Therapy (CHER) trial, ART started at a median of 7 weeks of age reduced early mortality by 76% and disease progression by 75% compared to standard of care (initiation of ART according to CD4% (<25%) or clinical progression). 67% of infants in the standard of care arm started ART during a median follow-up period of 40 weeks because of failing CD4%. A similar rapid decline in CD4% was observed in a further South African trial, in which 85% of untreated infants met WHO criteria for initiation of ART (CD4% <25%) by the age of 6 months [18].

2.3.3 *Monitoring Strategies: differences between adults and children*

CD4 cell counts are different in healthy children compared to adults. They are very high and variable in uninfected infants, falling slowly towards adult values by around mid-childhood [19]. CD4 percent is less variable than CD4 cell count, and is therefore more frequently used to monitor changes in immune function in children, particularly in the first five years of life [20]. Growth is an additional clinical tool for monitoring response to ART in children and in small trials has been associated with virological response to ART [21]. Finally, the frequency of adverse drug effects may differ in children because of the presence of different co-existing diseases (e.g. an increased frequency and severity of malaria-associated anaemia) compared with adults. For all these reasons, there ia a need for a separate paediatric trial assessing monitoring strategies, since results from the ongoing DART trial in adults may not necessarily be generalisable to children.

2.3.4 *First line ART regimens*

(i) Differences between children and adults

Worldwide, NNRTI-based regimens are the most widely prescribed combinations for initial triple drug therapy and are recommended as first-line ART in WHO guidelines on scaling up antiretroviral therapy in resource-limited countries [9]. The preferred first line treatment option for children includes stavudine (d4T) or ZDV + 3TC plus an NNRTI, usually NVP. However, WHO guidelines recommend that abacavir (ABC) can be a useful alternative NRTI in first-line ART for infants and children [9] and in addition, has been reported to have durable increased potency when combined with 3TC or ZDV compared with ZDV+3TC [22]. Most African countries are implementing first-line NNRTI-based regimens for adults and children. However, an important additional issue for children is the possibility they may be exposed to single-dose NVP in the perinatal period as prophylaxis to reduce mother-to-child HIV transmission.

The number of ART formulations available for children is limited. Although there are over 18 antiretroviral drugs commonly used in adults, only 10 antiretrovirals (mostly nucleoside

analogues) have suitable formulations and licensed doses for children of all ages (zidovudine, didanosine, lamivudine, stavudine, abacavir, emcitribine, nevirapine, ritonavir, nelfinavir and lopinavir). Efavirenz (EFV) cannot be used in children under 3 years of age, as a dose has not yet been identified due to problems in achieving appropriate drug levels. Amprenavir is a protease inhibitor (PI) with a paediatric formulation, but it is not available for all ages. There are no paediatric formulations of saquinavir or indinavir, and there is currently no approved paediatric dose of tenofovir or tipranavir [23]. Therefore questions relating to rational use of the available drugs are even more important in children than adults.

Further, untreated children generally have higher viral loads than adults, and typically have been reported to achieve virological suppression rates <50 copies/ml of only 24%-52% after 48 weeks of triple 2-class ART [24-28] compared to 80-90% in adults [29]. This may be due to a combination of higher pre-treatment viral loads, pharmacokinetics, and social factors affecting adherence to medication given by a caregiver.

(ii) 4-Drug first-line ART in adults and children

In adults, attempts to more completely suppress HIV infection have led to the investigation of combinations of 4 (or more) antiretroviral drugs as initial continuous therapy. Results from several trials comparing 4 to 3-drug regimens over a period of years are not conclusive, but overall there is no evidence to suggest superiority of continuous 4+ over 3-drug regimens in reducing viral load by levels detectable by currently available assays [Moyle G, personal communication]. In particular, the rate of initial viral load reduction, which has been associated with longer-term virological suppression [30], appears to be similar with 4 and 3-drug regimens in adult trials [31]. Of note, most of these trials have been relatively small, and further most 4-drug regimens included 3 drug classes, and cited increased toxicity as a major reason for discontinuation, and thus failure, of the strategy. The results of a recently published pilot study in 114 adults comparing a 4-drug NRTI regimen with a two class 3-drug regimen over 48 weeks suggested that these provided similar antiviral potency and tolerability but larger trials over longer periods are needed [32]

There have been no randomised trials comparing 4 versus 3-drug regimens in children. Small observational non-randomised single-centre studies in infants have noted better virological response with 4 compared to 3 drugs [26,33]. In the UK study [33], 30 (mostly symptomatic) infants received 2-class, 4-drug therapy with ZDV+3TC+ABC+NVP, so-called ‘baby cocktail’. This regimen has several advantages, in that all drugs are palatable liquids and 3TC and ABC are both the same small volume. After a median of 132 weeks, only 2 (6.7%) children had stopped any part of this regimen for toxicity (both for hepatic toxicity related to NVP) and at 36 and 48 weeks, by intention-to-treat analysis, HIV RNA was <50 copies/ml in 68% and 80%, and <400 copies/ml in 89% and 93% respectively (compared with 48% <50 copies/ml and 68% <400 copies/ml at 24 weeks). CD4 counts increased to normal values in these infants and there was substantial catch-up growth. Paediatricians caring for these children are now simplifying to 3 drugs and even to once daily regimens of 3TC+ABC+NVP (Lyall H, personal communication), as pharmacokinetic data show that 3TC and ABC can be given once daily to children after the age of 3 years [34]. Of interest, this finding of increased benefit with 4 drugs was also observed in a larger national observational study including more than 100 infants starting ART in the first year of life across the UK and Ireland [35].

(iii) Induction-maintenance strategies in adults

The rationale for an induction-maintenance approach to ART is to use a 4+ drug regimen to drive the viral load down to well below limits of detection, after which undetectable viral load might be maintained with a less potent 3-drug regimen for a longer period of time compared to using standard 3-drug therapy throughout. Evidence for this approach derives from three adult trials, although all are relatively small, and a small intensification study. In

the latter study ABC was added after 5 years to a regimen of EFV + the protease inhibitor (PI) Indinavir. In 8 of 14 patients an undetectable viral load was further suppressed by 0.6 log (4-fold) and numbers of activated memory cells were decreased [36].

Two trials of induction with 4 drugs (triple NRTI in the form of Trizivir [ZDV+3TC+ABC] plus EFV) followed by maintenance with Trizivir (TZV) have been carried out in antiretroviral naïve adults: neither included a comparative 3-drug arm and both randomised patients who had successfully suppressed on the induction regimen, therefore addressing a question as to whether virological suppression can be maintained on a maintenance regimen after 4 drug induction.

In the first study, AZL30004, [37] after successful induction with TZV+EFV for 24 weeks 245 patients were randomised to continue TZV+EFV for 48 weeks, to reduce to TZV for 24 weeks after a further 24 weeks of TZV+EFV or to reduce to TZV for 48 weeks. At 72 weeks, similar proportions had VL <50 copies/ml in an intention-to-treat analysis (75%, 76%, 74%). Drug related adverse events were reported in 11%, 10% and 4% of patients respectively and there were significant decreases in fasting lipids in the TZV maintenance arms compared with the 4-drug arm.

In the second trial, ESS40013, [38] after a successful induction with TZV and EFV for 48 weeks, 282 patients were randomised to TZV or TZV+EFV. Both arms had similar levels of viral load (VL) suppression <50 copies/ml at week 96 (77% TZV; 79% TZV+EFV), but adherence was poorer and there were more side effects in the continuous 4-drug arm. Overall 19% and 12% of patients in the AZL30004 and ESS40013 studies respectively stopped therapy due to adverse events during the 4-drug induction phase (i.e. prior to randomisation). Most events were CNS (related to EFV toxicity, around 7% in both trials), or possible hypersensitivity to ABC (7%, 9%).

The third trial, FORTE, (n=126) is the only trial to compare induction-maintenance with standard 3-drug therapy. Induction ART with a 4-drug 3-class regimen for 24-32 weeks was followed by continuation with a 3-drug 2-class regimen (2NRTI + NNRTI), and this was compared with 2NRTI + NNRTI therapy throughout [39]. Although there was no difference between the two arms in initial suppression rates, by 48 weeks 81% of those in the induction-maintenance arm had HIV-1 RNA <50 copies/ml compared with 65% in the 3-drug arm. This difference was maintained beyond 80 weeks, although numbers were small. Of interest in this trial, the frequency of adverse events was no higher in the 4-drug arm. This trial lends support to the idea that a 4-drug 3-class regimen may be able to drive viral load so far below detectable levels that rebound takes longer to occur compared to standard 3-drug 2-class regimens.

(iv) Induction-maintenance strategies in children

Given their high viral loads on starting therapy, induction-maintenance could be a valuable strategy in children 4-drug induction therapy could be more effective compared with 3 drugs in children, because in adults the proportion achieving suppression <50 copies/ml is already around 80% with standard 3-drug therapy, and there maybe a ‘ceiling’ effect in terms of improving this further. However, given the more disappointing viral suppression rates in children on 3 drugs, more substantial improvements may be possible by adding a fourth drug.

A randomised trial to evaluate the long-term effectiveness of an induction-maintenance approach in infants and older children could therefore be of great value for children worldwide if it could increase the durability of first-line therapy at the relatively small cost of adding one additional drug for a short period of time. Induction in the adult FORTE trial also included a PI. However, this would not be practical for inclusion in first-line therapy for children in Africa because of tolerability and interactions, notably with anti-tuberculosis therapies. Rather induction could be undertaken with the ’baby cocktail’ regimen used in the

UK study and increasingly being used by European paediatricians, and which appears to be potent and well tolerated in small observational studies in children.

(v) Maintenance regimens

In terms of maintenance regimens, inclusion of a 2 NRTI + NNRTI maintenance arm allows direct comparison with the standard 3-drug first line regimen. However, there are several advantages to considering triple NRTI after 4-drug induction – notably, lack of interactions with anti-tuberculosis drugs and potential for construction of second line regimens based on PI + NNRTI. This combination may be more potent than the PI + 2NRTI second line regimens which will be used in other arms of ARROW, given the substantial NRTI cross resistance that is likely to have arisen as a consequence of switching based on clinical/immunological, as opposed to virological, failure. Although triple NRTIs as first-line therapy have been shown in adult clinical trials to have statistically significantly lower virological suppression rates than EFV-containing regimens, rates of suppression are still high on triple NRTI regimens (61% with <50 copies/ml at 48 weeks in the ACTG 5095 trial compared with 83% in the EFV + 2NRTI arm) [40]. It is hypothesised that initial induction with 4 drugs will contribute to continued viral load suppression when reducing to a triple NRTI maintenance regimen.

(vi) Toxicity of ART in Children

Toxicity rates to ART appear to be lower in children than adults. A lower rate of nevirapine toxicity has been reported in the US [41]. Among 124 Zambian children in the CHAP2 cohort starting Triomune, a fixed dose combination of d4T, 3TC and NVP, with half or whole tablets, none had reported NVP toxicity requiring change in dose over the first 3 months, and NVP levels were almost all in the therapeutic range [42]

In an analysis of drug substitution among 410 children starting first-line ART after the year 2000 in the UK and Ireland Collaborative HIV Study (CHIPS) cohort, less than 10% substituted any drug during the first three months of therapy. Furthermore, 220 (54%) were taking ABC as part of their first-line regimen, and in 58 of these ABC was part of a 4-drug regimen (A Judd, DM Gibb, personal communication). This suggests lower rates of substitution for toxicity compared to adults. In African children, toxicity rates to abacavir are likely to be even lower because of polymorphisms leading to less ABC hypersensitivity. This was recently reported in adults in a double-blind substudy of DART, the Nevirapine Or Abacavir trial (NORA) [43].

2.3.5 Simplification of long-term ART

During the first year after initiating ART, children (and adults) may experience drug toxicity and continued higher rates of illnesses and hospitalisation, particularly if ART has been started relatively late in the course of disease. However, once this first year is past and children are stable on ART, it is increasingly clear that the major challenge to successful long-term outcomes is continuing high adherence and high acceptability of the ART regimen to children and carers. This is particularly true with return to health, when receding memories of ill-health reduce motivation to continue high adherence to ART. Identifying ways of making long-term ART more acceptable to children and carers is therefore central to maintaining benefit. (Note: increasing acceptability to carers is the rationale behind decreasing the frequency of nurse visits from 4-weekly to 6 weekly in protocol version 2.0 since taking time from work or other activities to attend clinic is one of the key issues for carers, particularly in view of the fact that many national programmes now have only 3-monthly follow-up appointments.)

Discussion with ARROW team members, carers and older children and a review of the published literature have identified two areas of management which could improve adherence: (i) Reducing the frequency of drug administration to once daily. Carers feel that once daily administration of medication would greatly simplify both their lives and those of their children. Morning dosing may be considered particularly difficult for working carers, who have to either wake the child very early in order to give medication before leaving home, or leave others to administer it. Alternatively evening dosing may be problematic if children are moving to alternative carers during the day. Preliminary data from the ARROW pharmacokinetic substudy demonstrates clearly that carers feel that once daily administration of medication (with the flexibility to give drugs either in the morning or in the evening depending on social circumstances) would greatly simplify theirs and their children's lives. (ii) Decreasing pill burden, one option being to stop daily administration of cotrimoxazole prophylaxis with ART.

(i) Once versus twice daily lamivudine+abacavir

Whereas lamivudine and abacavir are both licensed for once daily dosing in adults, based on results from several randomised trials, in children licensing remains twice daily. The CNA30021 trial compared once versus twice daily abacavir in 770 ART-naïve adults also initiating lamivudine+efavirenz once daily and found 66% and 68% patients had HIV RNA <50 copies/ml at 48 weeks respectively. The CAL30001 study compared once versus twice daily abacavir in 186 failing patients with HIV RNA >1000 copies/ml and NRTI resistance mutations initiating a new regimen also containing tenofovir and a PI or NNRTI, and found 50% and 47% patients had HIV RNA <50 copies/ml at 48 weeks. Finally the SEAL study compared switching to lamivudine+abacavir once daily versus continuing with twice daily dosing (with PI or NNRTI as the third drug) in patients with HIV RNA <400 copies/ml and found 82% and 81% patients had HIV RNA <50 copies/ml 48 weeks later respectively.

A major potential benefit of simplified once daily dosing, which maybe even more important for children, is improved adherence. This was demonstrated by a recent trial comparing electronically measured (MEMSCAP) adherence in 94 adults randomised to twice versus once daily lamivuding+abacavir; once daily dosing was associated with significant improvements on taking, dosing and timing compliance. Patient satisfaction was also significantly greater with once daily therapy.

The only published data on once daily dosing of lamivudine and abacavir in children are from relatively small pharmacokinetic studies demonstrating equivalence of area under the pharmacokinetic time-concentration curve. Questionnaire data from these studies showed that carers and children preferred once daily drug administration. Thus, anecdotally, many children receiving lamivudine+abacavir in resource-rich settings will be prescribed a once daily regimen with good rates of viral load suppression, even though this is not licensed for children, (H. Lyall, personal communication; A Judd, CHIPS, personal communication). Of note, these children were receiving doses based on mg/kg rather than the weight-band based dosing advocated by WHO and used in resource-limited countries. Weight-band doses were chosen to achieve the minimum recommended dose in each weight-band, and therefore should be appropriate for once daily dosing as in adults, but there are no data to confirm this. Given the large numbers of children who will receive ART in resource-limited settings, the lack of tenofovir (which is once daily) for children, and the major potential benefits from once daily dosing – particularly for adherence - it is essential to provide data in children demonstrated non-inferiority of once to twice daily lamivudine+abacavir dosing. The major concern about once daily dosing, which has not been demonstrated in adult trials from well-resourced countries described above, is that it could be more "fragile" - if a child misses one dose when they are taking lamivudine+abacavir twice a day they still get some drug –

whereas if they miss one dose when they are taking lamivudine+abacavir once a day they miss these drugs for the whole day.

(ii) Daily cotrimoxazole prophylaxis on ART

Randomised controlled trials have demonstrated that cotrimoxazole prophylaxis reduces mortality and hospitalisations in ART-naïve patients in resource-limited settings, but there are few data evaluating whether the same benefits occur in those on ART. In the absence of evidence, WHO currently recommend that cotrimoxazole prophylaxis should be continued with ART, but also recommended that more research be undertaken to examine this.

A major observational but confounding-controlled analysis of adults in the DART trial found a significantly reduced mortality in patients continuing to receive cotrimoxazole prophylaxis with ART versus those who stopped (OR=0.65 [95% CI 0.49-0.86]; p=0.003). However, whereas the mortality risk reduction was considerable during the first 72 weeks on ART (OR=0.43 [0.27-0.67] 0-12 weeks, OR=0.56 [0.36-0.86] 12-72 weeks), after 72 weeks cotrimoxazole no longer appeared to have any significant benefit (OR=1.09 [0.66-1.82]; heterogeneity p=0.02). Importantly, **this was not explained by current CD4;** mortality was reduced by approximately 50% in participants with current CD4<200 and CD4 ≥200 cells/mm^3 on cotrimoxazole prophylaxis and ART during the first 72 weeks were both about 50% and benefit stopped in both groups after 72 weeks. Results were similar for deaths that were potentially preventable with cotrimoxazole or deaths from other causes, but there was no evidence for an effect on new WHO 4 events. Four patients in the trial died of malaria, two of whom were on cotrimoxazole prophylaxis.

These findings suggest a definite benefit of cotrimoxaozle for at least 72 weeks on ART, regardless of current CD4 count, but no subsequent benefit longer term, again regardless of current CD4 count. Given the higher rate of bacterial infections in very young children, and uncertainty as to whether 72 weeks might really be the optimal time to stop cotrimoxazole prophylaxis, we consider that these findings support investigation of the impact of stopping cotrimoxazole prophylaxis in children who have been on ART for at least 96 weeks. For the youngest children, we would increase this time further by only enrolling children > 3 years of age.

Sulfadoxine-pyrimethamine (SP) - which, like cotrimoxazole, is an antifolate drug - is recommended by WHO for the prevention of malaria in pregnancy. Recent research has also demonstrated that intermittent preventive treatment with SP of asymptomatic infants attending routine Expanded Programme on Immunisation (EPI) clinics reduces episodes of clinical malaria and anaemia by approximately 30. Although daily cotrimoxazole prophylaxis is not recommended for the prevention of malaria, evidence from a number of sources suggests that it is effective at doing so. In DART, cotrimoxazole significantly reduced episodes of malaria (OR=0.71 [0.60-0.85]; p<0.001, an effect maintained over time), as did ART (patients randomised to continuous therapy had reduced malaria risk compared to those with structured treatment interruptions (OR=0.62 (95% CI 0.45-0.84) p=0.002). Only 4 DART deaths were from malaria, of which 2 were on cotrimoxazole. Further evidence that cotrimoxazole prophylaxis (in the absence of other anti-malarial interventions and in those taking on ART) prevents malaria and other infections comes from the randomised HBAC study which found lower rates of malaria (fever with parasites on thick smear, 0.4% vs 12.3% off cotrimoxazole) and self-reported diarrhoea (14% vs 25% off cotrimoxazole) in 836 adults with CD4 counts >200 cells/mm^3 and > 3 years on ART. This study was stopped at the first DSMB review after only 3.8 months and so the benefits of cotrimoxazole are likely to be overestimated. Furthermore whilst 4 patients died, 3 were in the cotrimoxazole group. At the presentation of these findings, the authors commented that early stopping of the trial left unanswered questions. It remains uncertain whether the observed increase in malaria in those

stopping cotrimoxazole was a rebound phenomenon which might not persist long term. It was unclear whether the frequency or severity of malaria and diarrhoea was any different from what might be observed in HIV uninfected individuals. Finally, the benefit of protection against malaria could be outweighed by the cost, toxicity and risk of drug resistance in the longer term.

Evidence for a protective effect of malaria in HIV-infected children on ART also comes from the non-randomised CHAMP study which compared malaria incidence over a period of 11 months among 300 HIV-infected children receiving cotrimoxazole prophylaxis and insecticide treated bednets with 561 HIV-negative children who initially received no anti-malarial intervention, but were given insecticide treated bednets during the last 4 months of the study. The use of insecticide-treated bednets was associated with a 43% reduction in malaria incidence (P<0.001), and a combination of cotrimoxazole prophylaxis and use of insecticide-treated bednets with a 97% reduction in malaria incidence (P<0.001). Only 12% of the HIV-infected cohort were on ART although the mean age was 5.6 years and median CD4 cell percentage 21%. Six HIV-infected children died during the study but none from malaria and no child developed severe malaria during the study. Given the substantial impact of ART itself on reducing malaria, it is unclear whether similar effects would be observed over the long-term in children receiving ART.
This view is supported by the Consultative Meeting on Cotrimoxazole Cessation Studies in Kampala who explicitly concluded, "Anecdotal evidence, the gap in policy and deliberations at this particular meeting confirm that there is genuine scientific equipoise regarding continuation or stopping use of cotrim as prophylaxis" and "There is good scientific rationale for continuing cotrim cessation studies in Uganda given that results from the Torro study left important research questions unanswered."

3.0 Objectives

The key objectives of the ARROW trial are to determine:

1) Whether Clinically Driven Monitoring (CDM) will have a similar outcome in terms of disease progression or death as routine Laboratory and Clinical Monitoring (LCM) for toxicity (haematology/biochemistry) and efficacy (CD4).
2) Whether induction with 4 drugs (2 ART classes) followed by maintenance with 3 drugs (as either one or 2 classes) after 36 weeks will be more effective over the longer term than a continuous NNRTI-based triple drug regimen in terms of CD4 and clinical outcome.

Two secondary randomisations aim to determine

3) Whether changing from twice daily lamivudine+abacavir to once daily lamivudine+abacavir after 48 weeks on ART will have a similar outcome in terms of virological suppression and will result in improvements in adherence to ART
4) Whether stopping daily cotrimoxazole prophylaxis in children over 3 years of age who have been on ART for at least 96 weeks has a similar outcome in terms of hospitalisation or death as continuing daily cotrimoxazole.

In addition, a number of sub-studies are planned. These include an assessment of adherence and acceptability of both tablet and liquid formulations, and pharmacokinetic, immunology/virology and metabolic sub-studies.

4.0 Endpoints

4.1 Primary Endpoints:

4.1.1 Monitoring practice (n=1200)

Efficacy:

- Progression to a new WHO stage 4 event or death (see Appendix 3.0, page 80.)

Safety:

- Any grade 3 or 4 adverse events (AEs), which are not solely related to HIV (see section 7.8, page 44 and Appendix 2.0, page 64).

4.1.2 ART strategies for first-line therapy (n=1200)

Efficacy:

- Change in CD4% at 72 and 144 weeks

Safety:

- Any grade 3 or 4 adverse events (AEs), which are not solely related to HIV (see section 7.8, page 44 and Appendix 2.0, page 64).

4.1.3 Once daily lamivudine+abacavir (n=630)

Efficacy:

- HIV RNA viral load at 48 weeks after randomisation (measured retrospectively)

Safety:

- Any grade 3 or 4 adverse events (AEs), which are not solely related to HIV, judged definitely/probably or uncertain whether related to lamivudine or abacavir (see section 7.8, page 44 and Appendix 2.0, page 64).

4.1.4 Cotrimoxazole prophylaxis (n=1000)

Efficacy:

- Hospitalisation or death

Safety:

- Any grade 3 or 4 adverse events (AEs), which are not solely related to HIV (see section 7.8, page 44 and Appendix 2.0, page 64).

4.2 Secondary Endpoints:

If not already stated above as primary endpoint, the following will be considered secondary endpoints for all randomisations

- New WHO stage 3 or 4 event or death *
- New or recurrent WHO stage 3 or 4 event or death *
- New WHO stage 4 event or death *
- Mortality
- Hospitalisations
- Height, weight and weight for height (body mass index), all standardised for age
- CD4 percentage and absolute count (at 72 and 144 weeks)
- Any grade 3 or 4 adverse events (AEs), which are not solely related to HIV (see section 7.8, page 44 and Appendix 2.0, page 64)
- Any grade 3 or 4 adverse events definitely/probably or uncertainly related to ART (see Appendix 2.0, page 64).
- Serious adverse events that are not HIV related only
- ART-modifying adverse events (any grade)
- Number and class of anti-HIV drugs received

- Time to cessation of first-line regimen for clinical/immunological failure
- HIV RNA viral load and resistance (retrospectively) at 72 and 144 weeks (3 years)
- Adherence to ART as measured by visual analogue scale, self-reported questionnaire and clinic-based pill counts

* including and excluding oral/oesophageal candida which is widely recognised as a less severe (and always non-fatal) WHO 3/4 event. For LCM vs CDM efficacy endpoints, non-inferiority will be judged on the basis of new WHO 4/death, and new WHO 3 or 4 or death (excluding oral/oesophageal candida).

In addition for the once versus twice daily lamvudine+abacavir randomisation, the following will be considered as specific secondary endpoints:

- HIV RNA viral load at 96 weeks after randomisation (measured retrospectively)
- Change in CD4 cell counts at 48 and 96 weeks after randomisation

In addition for the cotrimoxazole prophylaxis randomisation, the following will be considered as specific secondary endpoints

- Severe pneumonia
- Severe recurrent pneumonia or diarrhoea (using defintions for WHO stage 3 events)
- Clinical and smear positive malaria [Clinical malaria (no diagnostic) is going to be of highly dubious value, given the low proportion of fevers in ART-treated children that are due to malaria]. Diagnostic: either microscopy (thick film) or rapid diagnostic test (RDT)

5.0 Design

5.1 Type of design

ARROW is an open, parallel group, four-centre, two-country, randomised controlled, clinical endpoint trial in HIV infected children in Africa. There will be two primary randomisations at enrolment (ART initiation), 2 further randomisations post-enrolment (for which additional consent will be sought) and the total duration of the trial will be 5 years.

5.1.1 Randomisation at enrolment to CDM or LCM arm

At ARROW enrolment 1200 children will be randomised to CDM or LCM over a period of 18 months.

5.1.2 Randomisation to at enrolment ART strategies for first-line therapy

At ARROW enrolment 1200 children will be randomised to either a control arm or one of two induction-maintenance arms for first line ART, to be taken once or twice daily (depending on age and regimen):

Arm A (standard): NNRTI + ABC +3TC continuously

Arm B (induction maintenance): NNRTI + ZDV + ABC + 3TC for 36 weeks, then NNRTI + ABC +3TC

Arm C (induction maintenance): NNRTI + ZDV + ABC + 3TC for 36 weeks, then ZDV + ABC + 3TC

5.1.3 Randomisation after at least 36 weeks on ART to once or twice daily lamivudine+abacavir

All carers of eligible children and older children where appropriate, taking lamivudine+abacavir twice daily as either syrups or tablets as part of ART (estimate at least n=630), who give informed consent/assent will be randomised to continue twice daily or move to once daily lamivudine+abacavir. By 1 July 2009 (estimated randomisation start date) all but 6 children will have reached 36 weeks on ART, so could be randomised to twice versus once daily if taking lamvidine+abacavir (1189 children). We estimate that all eligible children could be recruited over a period of 24 weeks (ie by end of 2009), and so the last recruited child would reach week 48 in November 2010, and week 96 in October 2011.

5.1.4 Randomisation after at least 96 weeks of ART to continue or stop daily cotrimoxazole prophylaxis

All carers of eligible children aged 3 years or more and older children where appropriate, who have been on ART for at least 96 weeks (estimate at least n=1000) and who give informed consent will be randomised to stop or continue daily cotrimoxazole prophylaxis. As of January 2009 1171/1207 (97%) enrolled children were in follow-up. By 1 July 2009 (estimated randomisation start date) 13% will have already reached 96 weeks and could be randomised immediately. The remaining children will reach 96 weeks on ART by September 2010, leading to an accrual period of 15 months.

All children living in malaria endemic areas will be provided with insecticide treated bednets (if these are not already available). This randomisation will not be blinded (ie placebo will not be used in the stop arm) because one of the primary aims is to determine whether stopping daily cotrimoxazole prophylaxis improves adherence to ART.

For both 5.1.3 and 5.1.4, the target is 630 and 1000 randomisations respectively (see sections 10.4 and 10.3 on p55): however, all eligible children in ARROW whose carers consent to randomisation will be enrolled to maximise our power to address these critical questions.

5.2 Antiretroviral Therapy

See section 8.0, page 47 and Appendix 1.0, page 61 for full details. First and second-line regimens will be available for all participants during the trial period, as well as the option of changing individual drugs (within the same class) for toxicity (see sections 7.5, page 43). For patients in whom second-line therapy fails, the most appropriate combination of available drugs will be offered depending on first and second-line therapy received (see section 8.0, page 47). Every effort will be made to make further ART available after the trial but it is agreed that local health authorities will commit to providing adequate care for the participating patients after the end of the trial period).

6.0 Patient Population

6.1 Inclusion Criteria at ARROW enrolment

1. Children should have an adult carer in the household who is either:
    - participating in the DART trial ***OR***
    - being treated with ART ***OR***
    - HIV positive but not yet needing treatment but with access to a treatment programme when ART is required ***OR***
    - HIV negative.

Children of DART participants should have first priority on any available remaining slots to enter ARROW.

2. Parents or guardians, and children where appropriate according to age and knowledge of HIV status, must be willing and able to give informed consent for randomisation to CDM or LCM **and** to first-line ART strategy.
3. Participants must have a confirmed documented diagnosis of HIV-1 infection:

a) For children aged under 18 months: two separate peripheral blood specimens from different days, both results being positive with HIV-DNA PCR.

b) For children aged 18 months or over: antibody positive serology by ELISA test (confirmed by licensed second ELISA or Western Blot) or WHO approved rapid test (performed in series) both on the same sample. Any child previously tested at another clinic should have a repeat test at an ARROW screening laboratory to confirm their status.

4. Age 3 months to 17 years (13-17 years to be capped at 10%)
5. ART naïve (except for exposure to perinatal ART for the prevention of mother-to-child HIV transmission).
6. Meeting criteria for requiring ART according to WHO stage and CD4 percent or count:

- WHO paediatric clinical stage IV disease: treat regardless of CD4 percent or count
- WHO paediatric clinical stage III disease:
  - <12 months: treat all
  - $\geq$12 months: treat all children irrespective of the CD4 percent or count; however, in children aged $\geq$ 12 months with tuberculosis**,** lymphocytic interstitial pneumonia (LIP), oral hairy leukoplakia (OHP) or thrombocytopenia (low platelet count treat) be guided by CD4 cell assays (see below).
- WHO paediatric clinical stage II or I disease: treat guided by CD4 percent or count
  - CD4%<25% for infants <12 months;
  - CD4%<20% for children 1-<3 years;
  - CD4% <15% for children 3-<5years;
  - CD4% <15% for children $\geq$ 5years (consideration should also be taken of the CD4 count. A CD4 count <200 cells/mm^3 can be used to guide starting ART and CD4 should generally be <350 cells/mm^3.)

6.2 Exclusion criteria at ARROW enrolment

1. Cannot, or unlikely to attend regularly (e.g. usual residence too far from study centre)
2. Likelihood of poor adherence
3. Presence of acute infection (e.g. malaria, helminthiasis, acute hepatitis, acute pneumonia, septicaemia, meningitis). Children may be admitted after recovery of an acute infection. Children with chronic lung disease, including recurrent respiratory infections, are eligible. Children with tuberculosis (TB) will not be enrolled while on the intensive phase of anti-tuberculosis therapy, but should be re-evaluated after the intensive phase and a decision made then about starting ART (see 4 below)

4. In receipt of medication contraindicated by ART
   - children under three years of age receiving anti-tuberculosis therapy should not be enrolled (as they will have to receive nevirapine).
   - on chemotherapy for malignancy
5. Laboratory abnormalities which are a contra-indication for the child to start ART (haemoglobin <8.5g/dL; neutrophils $<0.50x10^9/L$; AST or ALT >5 x the upper limit of normal (ULN); grade 3 renal dysfunction - creatinine >1.9 x ULN).

   N.B. causes of anaemia, such as concurrent bacterial infection, malaria, helminthiasis and/or malnutrition should be investigated, and treatment for anaemia and its causes commenced prior to re-screening for eligibility.
6. Being pregnant or breast-feeding an infant
7. Perinatal exposure to NVP (either through PMTCT or breastfeeding) for children aged 3 - 6 months only

6.3 Number and source of subjects for ARROW enrolment

Children will be recruited from four sites MRC/UVRI, Entebbe, Uganda; JCRC, Kampala, Uganda; PIDC/Mulago, Kampala, Uganda and UZ-CRC, Harare, Zimbabwe. This trial requires that participants be committed to long-term follow-up. Therefore priority will be given to those who can regularly attend for follow-up. Children and adolescents will be recruited from four sources:

1. Children/adolescents of families where at least one parent is enrolled in DART
2. Children/adolescents who have been tested for HIV infection in the past and are in regular follow-up, and whose parents/caregivers either have HIV infection with access to ART if required, or whose caregivers are not HIV infected.
3. Following ***recovery*** from a hospital admission during which HIV infection has been diagnosed, and followed by attendance at a follow-up outpatient clinic (N.B. no child should be recruited during a hospital admission);
4. From outpatient clinics (e.g. general medical, malnutrition, TB clinics). For children newly diagnosed with HIV, the HIV counselling and testing process should be fully carried out (for children with acute malnutrition, this should be managed before starting ART).

6.4 Once or twice daily lamivudine+abacavir randomisation

6.4.1 *Inclusion criteria*

1. Participating in ARROW
2. On ART for at least 36 weeks
3. Currently taking lamivudine+abacavir twice daily as part of their ART regimen and expected to stay on these two drugs for at least the next 12 weeks
4. Parents or guardians, and children where appropriate according to age and knowledge of HIV status, must be willing and able to give informed consent for randomisation to once or twice daily lamivudine+abacavir

6.4.2 *Exclusion criteria*

5. Likely to switch to second-line therapy in the next 12 weeks (see section 7.5.2, p43)

6.4.3 *Number and source of subjects*

Children will be recruited from the 4 ARROW clinical centres, MRC/UVRI, Entebbe, Uganda; JCRC, Kampala, Uganda; PIDC/Mulago, Kampala, Uganda and UZ-CRC, Harare, Zimbabwe. We estimate that at least 630 children will be enrolled.

6.5 Stop or continue cotrimoxazole prophylaxis randomisation

6.5.1 *Inclusion criteria*

1. Participating in ARROW
2. Aged at least 3 years
3. Initiated ART at least 96 weeks previously, and received at least 96 weeks of ART allowing for any interruptions in ART
4. Currently prescribed daily cotrimoxazole as primary prophylaxis
5. Parents or guardians, and children where appropriate according to age and knowledge of HIV status, must be willing and able to give informed consent for randomisation to stop or continue daily cotrimoxazole prophylaxis
6. If living in a malaria endemic area, has an insecticide treated bednet and prepared to use this for the child.

6.5.2 *Exclusion criteria*

7. Previous diagnosis of *Pneumocystis jiroveci* pneumonia (cotrimoxazole is secondary prophylaxis and should not be discontinued)

6.5.3 *Number and source of subjects*

Children will be recruited from the 4 ARROW clinical centres, MRC/UVRI, Entebbe, Uganda; JCRC, Kampala, Uganda; PIDC/Mulago, Kampala, Uganda and UZ-CRC, Harare, Zimbabwe. We estimate that at least 1100 children will be enrolled.

7.0 Procedures and Management of Subjects

7.1 Screening Procedure pre ARROW enrolment

At screening carers will be given an information sheet about the ARROW trial and asked to give written consent for screening (see Appendix 6, page 92). Children will also be given information and counselling appropriate to their age and should give assent to screening (if capable). After consenting to screening the child will have clinical information including medical history, examination, confirmation of paediatric WHO stage 2, 3, or 4, and weight recorded, and T cell subsets (CD4, CD8, CD3 and total lymphocyte count), haematology and biochemistry. A pregnancy test will be performed if considered appropriate by the trial clinician. Plasma and DNA pellets will be saved. For children being screened at Ugandan sites, peripheral blood mononuclear cells (PBMCs) will also be stored and immunophenotyping carried out on the blood sample taken for haematology and lymphocyte subsets, in order to ascertain T cell immune activation status. The volume of blood drawn for these additional analyses will not exceed the previous total draw of 8-10mls, as per protocol version 1.0. Girls of reproductive age will be given information about the risks of pregnancy in the trial and encouraged to avoid pregnancy.

The primary carer will be asked to nominate another one or two carers (depending upon local practice) who will be responsible for the child's welfare in the event that they are unable to

continue caring for them. Whilst the carer who consented is alive, counselling of any other person who brings the child to the ARROW clinic is necessary to explain the trial, but re-consent is not needed. If the carer who originally gave consent dies, it will be necessary to obtain re-consent from the next nominated primary carer.

7.2 Baseline procedure (Week 0)

The time between screening and randomisation should preferably be 1-2 weeks and not exceed 4 weeks.

The child's eligibility for enrolment will be confirmed. Children with CD4 percent or count above that for inclusion in the trial (WHO criteria for ART initiation) can be enrolled later if the CD4 percent or count falls to below the level for inclusion and they are still in follow up. Those children who the clinician considers need to start ART for clinical reasons (WHO staging) and/or whose CD4 percent or count is within the level for inclusion, and who have no haematological or biochemistry contraindications to starting ART, will be eligible for the trial.

There will be further counselling of the carer (and of the child as appropriate to their age) before enrolment. Fully informed signed consent from the carer will be obtained before randomisation (see Appendix 6, page 92). Consent forms should be kept securely at the clinical site and made available for monitoring. If they are old enough, and know their HIV status, the child should also give assent. After consenting, blood samples will be taken for T cell subsets (CD4, CD8, CD3 and total lymphocyte count) and QuantiFERON®-TB Gold In-Tube system, and plasma and DNA pellets will be saved. For children enrolled at Ugandan sites, immunophenotyping will be carried out as described above if this could not be achieved at screening. Blood volume drawn for these additional analyses will still not exceed the previous total draw of 8-10mls, as per protocol version 1.0. When possible, and particularly if there is doubt about the place of residence of a child and his/her family, a study nurse/field worker, preferably the same nurse who gave information about the trial to the carer/child, will accompany the family to their home and draw a map indicating the place of residence.

A trial register will be kept at the clinical site and will record all children who are eligible and invited to join the trial. Those accepting will have name, date of birth (DOB), randomisation date and trial number recorded. Those who refuse will have name, DOB, and reason for refusal recorded. The register will be kept in a secure place in each clinical site and will be the responsibility of the trial investigator at that site.

7.3 Randomisation and Enrolment

7.3.1 *Randomisation List*

A randomisation list for CDM/LCM will be prepared by staff at the MRC CTU under the direction of the trial statistician. Patient randomisation numbers will be prepared separately for each centre and further stratified within centre by age (3 months to 6 years; 7 years to 12 years; 13 years and older). A single member of staff at each site who is not directly involved in patient care will be responsible for carrying out the randomisation process using a secure electronic system within the trial database. A reliable manual back-up system will also be available. Randomisation will not take place until after informed consent has been given and the child is ready to receive therapy.

A randomisation list will be similarly prepared for randomisation to ART strategies for first-line therapy. This will be stratified by centre, age (3 months to 6 years; 7 years to 12 years; 13 years and older) and by first randomisation to CDM/LCM.

Randomisations in ARROW will be made without reference to the randomised allocations of parents/guardians in the adult DART trial. Thus a mother might be on LCM but her child on CDM. This will be discussed during the counselling procedure prior to consent.

A randomisation list will be similarly prepared for randomisation to once or twice daily lamivudine+abacavir. This will be stratified by centre, whether the other ART drugs are taken once or twice daily (ie if randomised to once daily abacavir+lamivudine will the child move to a completely once daily regimen or not) and by first randomisation to CDM/LCM.

A randomisation list will be similarly prepared for randomisation to stop or continue cotrimoxazole prophylaxis. This will be stratified by centre, first-line ART strategy (Arm A, B or C) and by first randomisation to CDM/LCM.

7.3.2 *First Randomisation to CDM or LCM*

The first randomisation is to CDM or LCM. The patient's eligibility for enrolment will be reviewed by the doctor at baseline (week 0, see flowsheets section 1.2.1, page 15), taking into consideration the information from the screening case record form (CRF), including the results of laboratory tests. Patients should start ART and be told their allocation on the day of randomisation.

7.3.3 *Second Randomisation to ART strategies for first-line therapy*

The second randomisation is to either continuous or induction-maintenance ART strategies for first-line therapy. Children will be randomised immediately after their first randomisation to CDM or LCM. All children initiating NVP will use a standard dose escalation strategy for the first two weeks.

7.3.4 *Pregnancy*

As adolescents up to age 17 will be recruited to the trial it is possible that some may be pregnant at screening or become pregnant during the trial. Any girls who are found to be pregnant at screening will not be eligible for the trial but may be re-screened to ascertain eligibility for enrolment into the trial after the baby is born and breast-feeding has finished, provided that enrolment is still open at that site.

However, girls who become pregnant during the trial will continue in follow-up and will not be excluded.

7.3.5 *Enrolment of siblings into the trial*

If more than one child from the same family is eligible and carers give consent to enrolment in the trial, they will be allocated to CDM or LCM without consideration as to the randomisation of the family member already enrolled. Carers may also have been randomised to a different monitoring strategy in the main DART trial. The same will be true for the randomisation to the first line ART strategy. This must be fully explained to family members where more than one child is recruited into the trial.

7.3.6 *Randomisation to once or twice daily lamivudine+abacavir*

The patient's eligibility for enrolment will be reviewed at the next ARROW visit immediately after this randomisation opens (following ethical approval). The carers (and child where appropriate) of any potentially eligible child will be provided with the Lamivudine+Abacavir Patient Information sheet at this visit, and will have opportunities to

ask questions from the counsellors and nurses. At the next **scheduled doctor visit** carers (and children as appropriate to their age) of eligible children will be approached for fully informed signed consent from the carer. If they are old enough, and know their HIV status, the child should also give consent. Consent forms will be kept securely at the clinical site and made available for monitoring. Following consent (and assent as appropriate) and confirmation of eligibility, the child will be randomised to once or twice daily lamivudine+abacavir, which should be prescribed from this visit as randomised. If the child is randomised to once daily lamivudine+abacavir and is also taking their other ART drugs once daily, they may move to taking all their drugs either in the morning or at night as preferred. Once it is decided for a given patient that they will opt for morning or evening dosing, they should not switch between the two.

A register will be kept at the clinical site and will record all children who are eligible and invited to join the twice vs once daily lamivudine+abacavir randomisation. Those accepting will have name, date of birth (DOB), randomisation date and ARROW trial number recorded. Those who refuse will have name, DOB, ARROW trial number and reason for refusal recorded. The register will be kept in a secure place in each clinical site and will be the responsibility of the trial investigator at that site.

Randomisation will be performed at a scheduled doctor visit (12 weekly from ART initiation) so that all laboratory tests will be done 12 weekly from randomisation to once or twice daily lamivudine+abacavir.

7.3.7 *Randomisation to stop or continue daily cotrimoxazole prophylaxis*

The patient's eligibility for enrolment will be reviewed at the next ARROW visit immediately after this randomisation opens (following ethical approval). The carers (and child where appropriate) of any potentially eligible child will be provided with the Cotrimoxazole Patient Information sheet at this visit, and will have opportunities to ask questions from the counsellors and nurses. At the next **scheduled doctor visit** carers (and children as appropriate to their age) of eligible children will be approached for fully informed signed consent from the carer. If they are old enough, and know their HIV status, the child should also give assent. Consent forms will be kept securely at the clinical site and made available for monitoring. Following consent (and assent as appropriate) and confirmation of eligibility, the child will be randomised to stop or continue daily cotrimoxazole prophylaxis, which should be stopped at this visit if randomised to the stop arm otherwise continued at the previous dose. Doctors must ensure that the carer has an insecticide treated bednet for any child living in a malaria endemic area randomised to the stop cotrimoxazole prophylaxis arm.

A register will be kept at the clinical site and will record all children who are eligible and invited to join the cotrimoxazole randomisation. Those accepting will have name, date of birth (DOB), randomisation date and ARROW trial number recorded. Those who refuse will have name, DOB, ARROW trial number and reason for refusal recorded. The register will be kept in a secure place in each clinical site and will be the responsibility of the trial investigator at that site.

Randomisation will be performed at a scheduled doctor visit (12 weekly from ART initiation) so that all laboratory tests will be done 12 weekly from randomisation to stop or continue cotrimoxazole prophylaxis.

7.4 Follow-up Evaluations

After randomisation (week 0) all children will have follow-up visits to see both the study doctor and study nurse at weeks 2, 4, 8, 12 and then every 12 weeks from randomisation. The visits will be timed to coincide with the parent/guardian's DART visits where appropriate. The carer will collect a supply of drugs every 6 weeks (4 weeks before week 48 and in protocol version 1.1) from the nurse at the follow-up and intermediate visits (see flowsheet, section 1.2.1, page 15).

At each full assessment (12 weekly), the following will be undertaken:

- ♦ Administration of a symptom checklist by a nurse to detect intercurrent illness, HIV disease progression or adverse events to ART. The severity and likely relationship of any adverse events will be documented by a doctor.
- ♦ Medical history since last visit including adverse events to ART, signs and symptoms of HIV disease and paediatric WHO stage.
- ♦ Weight and height, waist, hip and mid-upper arm circumference, plus head circumference in children under 5 years and Tanner scales (every 24 weeks) in children aged 10 years or older.
- ♦ Haematology and biochemistry (as at baseline). Regardless of whether the child is in the CDM or LCM arm of the trial, all week 8 haemoglobin results will be returned to the clinic to monitor for ART-related anaemia. For children in the CDM arm other haematology and biochemistry results will only be returned to the clinic if they are grade 4 (see Appendix 2.0, page 64).. In the case of biochemistry results, if any of AST/ALT/bilirubin are grade 4, all three results will be returned to the clinic; similarly, if either of urea/creatinine are grade 4, both results will be returned to the clinic. Additional biochemistry and/or haematology investigations may be requested for children in the CDM arm with symptoms suggestive of antiretroviral drug toxicity if the test result will help to guide clinical management.. For example, a decision to switch drugs on account of toxicity may be made on the basis of biochemistry results indicating hepatitis, renal disease or pancreatitis. The reasons for requesting laboratory tests must be clearly stated on the appropriate form and authorised by the project leader.
- ♦ CD4 cell count and percent: for children in the CDM arm, results will not be returned to the clinic.
- ♦ Plasma store and DNA pellets (see Appendix 4.0, page 69); stored plasma samples and DNA pellets may be used subsequently for HIV RNA viral load measurements, measurements of cell associated HIV DNA, HLA typing and analyses of drug levels.
- ♦ Assessment of adherence to ART by pill counts and nurse administered questionnaire (see Appendix 7.0, page 116). Adherence to cotrimoxazole prophylaxis will also be assessed in children taking it.
- ♦ Changes in ART, OI prophylaxis and other concomitant medication.
- ♦ All girls of childbearing age (aged $\geq$ 12 years who have reached Tanner stage 4) will be given continuing advice about avoiding pregnancy.
- ♦ If a girl becomes pregnant during the course of the trial, she will continue in the CDM or LCM arm like any other trial participant. Advice on breast-feeding will be given according to national guidelines.

- ♦ Compliance with the allocated management strategies will be recorded on the follow-up form.
- ♦ Children with fever and/or other symptoms suggestive of malaria should all have thick and thin films performed. In patient diagnoses should be recorded on the hospital admission form and outpatient on the doctor follow-up form, both with appropriate laboratory forms.

Note: children will be randomised to stop/continue cotrimoxazole prophylaxis and/or to once/twice daily lamivudine+abacavir at one of these 12 weekly visits. Therefore all the above assessments will be completely 12 weekly following these randomisations as well.

At specific designated 4 or 12 weekly visits the following will be undertaken:

- ♦ For children screened and enrolled at Ugandan sites, immunophenotyping to identify additional T cell subsets (CD45, CD31, HLA-DR and Ki67 as described at screening) will be recorded at weeks 4, 12, 24, 36, 48 and then every 24 weeks. If a child switches to second line ARVs then immunophenotyping will be done at the time of switch and then 12, 24 and 48 weeks post switch at scheduled visits. At all time points indicated bloods are to be taken from the same total blood draw as for routine haematology and lymphocyte subsets.
- ♦ For children enrolled at all sites, QuantiFERON®-TB Gold In-Tube system supernatants (as described at week 0) are stored at weeks 4, 36, 72, 120, 168 and 216 for later research into cytokine and lipopolysaccharide (LPS) levels, and its value for predicting development of subsequent TB in children. Therefore, in addition to this, all children diagnosed with TB will have Quantiferon stored at the time of TB diagnosis and 3 months after diagnosis. (This is because unpublished data from uninfected children (ie with good immune systems more similar to those HIV-infected children on ART in ARROW) who develop TB in the UK show that often the Quantiferon IGRA (Inteferon-gamma release assay) from a sample taken at TB diagnosis is **not** positive, but then becomes positive after a few weeks on TB treatment, that is, that the paediatric immune system does not initially recognise the TB antigens (B Kampmann, personal communication). PBMCs (as described at screening) are stored at weeks 12, 24, 48, 96, 144, 192 and 240 only for children enrolled at Ugandan sites, and may be subsequently used for measurements of HIV DNA and HIV RNA transcripts and T-cell receptor excision circles (TRECS). . If a child switches to second line ARVs then PBMCs will be stored at the time of switch and then 12, 24 and 48 weeks post switch at scheduled visits. Quantiferon and PBMCs are taken from the same total blood draw as for routine plasma storage. Consent for the future use of samples is included in the trial consent form (see Appendix 4.Appendix 6.0, page 69).

At each 'drug collection' visit (6 weekly (4-weekly before week 48)), the study nurse will:

- ♦ Administer the symptom check-list
- ♦ Assess adherence to ART and cotrimoxazole prophylaxis (if receiving it) by administering a questionnaire with a Visual Analogue Scale and also by recording numbers of returned pills/ bottles.
- ♦ Administer the next 6 weeks supply of drugs to last until the next clinic visit

If the study nurse has any clinical concerns at these visits, the patient should be referred to see a study doctor.

Children in both arms may undergo all necessary diagnostic tests for clinical management of illness. Where blood is drawn for diagnosis outside of the routine blood sampling schedule, any blood that would normally be discarded should be stored as plasma for retrospective analysis of new diagnostic tests. No extra blood should be taken from sick children, only discard stored.

7.5 Changing ART

7.5.1 *Substituting for Toxicity*

Substituting drug(s) for severe clinical or laboratory toxicity will follow guidelines based on clinical and laboratory grading of toxicities (Appendix 2.0, page 64). A symptom checklist will include questions on nausea/vomiting, rash, headache, fever, jaundice, abdominal pain etc. In the CDM arm, laboratory tests will be done at 4, 8 and 12 weeks and then 12-weekly but results will ***not be given back routinely to clinicians*** unless a laboratory grade 4 adverse event occurs. The only exception is haemoglobin at week 8 which will be returned for all children in order to manage anaemia appropriately. A system will be set up at each site (with standard operating procedures (SOPs)) for feedback of grade 4 results only to clinicians for children in the CDM group (following the successful model used in the DART trial). Clinicians caring for children allocated to CDM may request laboratory tests if indicated by clinical symptoms, as in routine practice: all such requests will be authorised by the Project Leader in each site. For guidelines on substituting individual drugs for toxicity, see section 8.1.4 page 48, and the Manual of Operations (MOP).

7.5.2 *Switching for failure of first-line therapy*

Physicians will be ***encouraged not to switch*** from first-line ART before 48 weeks on ART. Every attempt should be made to ensure that children are adherent to ART before a switch of ART is considered. After 48 weeks, switching therapy can be considered at any time.

Clinical criteria for consideration of switching therapy include:

WHO staging criteria:

- ♦ The development of a new or recurrent paediatric WHO stage 4 diagnosis (see Appendix 3.0, page 80). In the event of a child developing recurrent or persistent WHO stage 3 events, particularly if multiple (e.g. persistent oral candida with ongoing unexplained persistent diarrhoea and weight loss), switching therapy may also be considered at the physician's discretion.

CD4 cell count criteria:

- ♦ CD4 <15% if aged 1-<3 years, <10% if 3-<5 years, <100 cells/mm^3 if 5 years or older while on ART (confirmed) i.e. if a CD4 cell count is <15% (<10%, <100 cells/mm^3) respectively then it should be repeated as soon as practical and if still below those levels the patient should switch ART (these criteria are based on CD4 recommendations for immediate switching for failure in the WHO guidelines) [9].

When a decision to change therapy has been taken, blood will be taken for haematology and biochemistry screens from all children (CDM and LCM). A CD4 cell count will also be taken, but the result will not be returned to the clinician in the CDM arm, and a plasma specimen for future evaluation of HIV-RNA viral load and drug resistance will be collected. For children at the Ugandan sites who are participating in the immunology study, blood will also be taken for immunophenotyping and PBMCs. Children will restart the visit sequence at week 0 (see flowsheet 1.2.1, page 15) to ensure that the switch to new drugs is intensely monitored during the period immediately after the switch.

7.6 Assessment of adherence

Adherence to ART will be assessed in all participants in the ARROW trial at each 6-weekly visit (4-weekly before week 48) by pill counts for tablets, weighing of bottles for syrups, and nurse-administered adherence questions including a visual analogue scale. Every 12 weeks, a detailed adherence questionnaire will be administered to the child's carer, and where appropriate to the child (at the discretion of the nurse or doctor) (see Appendix 7.0, page 116). Adherence to cotrimoxazole prophylaxis will also be measured by questionnaire and pill counts.

7.7 Withdrawal from allocated strategy

Participants may voluntarily withdraw from allocated trial treatment (LCM, CDM, induction-maintenance regimen, cotrimoxazole prophylaxis strategy, once/twice lamivudine+abacavir dosing strategy) for any reason. Children may withdraw or be withdrawn from the induction-maintenance regimen, cotrimoxazole prophylaxis strategy, once/twice lamivudine+abacavir dosing strategy and remain in ARROW under their LCM/CDM strategy receiving ARROW drugs without affecting their care in ARROW. If they wish to withdraw from the LCM/CDM randomisation they will be referred to another ART provider in the national health system. Although trial researchers are not under obligation to provide ART, the child's withdrawal from the trial will not affect their access to the best standard of care within the national health system. Consent for withdrawal from the trial will be obtained (see Appendix 8.0) Follow-up for documentation of any clinical endpoints should continue, if possible, either through clinic attendance or medical notes. Consent for such follow up will be obtained.

Clinical data, including weight, presence of signs or symptoms, and other concomitant medications should be recorded at the time of withdrawal. In addition, a blood sample for plasma storage should be taken (for subsequent viral load and possible resistance testing). Blood should also be taken for FBC, biochemistry and T-cell subsets (not returned to clinicians of children in the CDM arm).

If a patient has an unscheduled period off treatment or is not in follow-up, this should be fully recorded on follow-up forms.

7.8 Recording and reporting of Adverse Events, Death, HIV progression and protocol violations

If the child has died, experienced any new or recurrent WHO stage 3 or 4 illness or experienced an adverse event (serious, Grade 3 or 4, or one leading to a modification of ART) since the last visit, the investigator/study co-ordinator will complete specific case report forms.

If there is any violation of the protocol for any reason, this should be fully recorded.

7.8.1 *Serious Adverse Events (SAE), Serious Adverse Reactions (SAR) and Suspected Unexpected Serious Adverse Reactions (SUSAR):*

A Serious Adverse Event (SAE) is defined as any untoward medical occurrence resulting in any of the outcomes listed below. A Serious Adverse Reaction (SAR) defines a response to study drug resulting in any of the outcomes listed below. "Suspected Unexpected" refers to

an adverse experience associated with the study drug that has not been previously observed, i.e. for which the specificity or severity is not consistent with the risk information provided by the manufacturer.

1. **results in death**
2. **is life-threatening** (patient was actually at risk of death at the time of the event)
3. **requires unplanned inpatient hospitalisation** or **prolongation of existing hospitalisation**
4. **results in persistent or significant disability/incapacity** or is a congenital anomaly/ birth defect
5. any **other important medical condition, which, though not included in the above, may jeopardise the subject and may require medical or surgical intervention to prevent one of the outcomes listed** (e.g. intensive treatment in hospital or at home for allergic bronchospasm; blood dyscrasia or convulsions that do not result in hospitalisation, or the development of drug dependency)

In the context of the ARROW trial, SAEs need only be reported if considered **not** HIV-related**.** All such SAEs must be recorded on the Serious Adverse Event form and reported to the MRC CTU as soon as possible after the event.

The MRC CTU will report SAEs that are considered definitely or probably causally related to antiretroviral study medication to the relevant pharmaceutical companies in a timely fashion, as agreed in the contract,. All suspected hypersensitivity reactions must be reported as SAEs, and these should be categorised as "other important medical conditions" unless they fall into one of the more specific categories.

National requirements for reporting of SAEs to regulatory authorities will be followed locally by each site.

Data on SAEs will be tabulated and presented to the Data Monitoring Committee (DMC; see section 9.4, page 51). Other grade 3 and 4 adverse events (see Appendix 2.0, page 64) should be reported at the next scheduled follow-up.

7.8.2 *Procedures for reporting of SAE, SAR and SUSAR:*

1. The SAE form should be completed by the investigator at the clinical site. The ARROW Trials Centre should email or fax the form to CTU immediately where the SAE will be assessed by the medical expert for the likelihood that it is related to antiretroviral therapy or other medication.

   MRC CTU **Fax Number: + 44 (0) 20 7670 4814**

2. Relation to study drug: If it is decided the SAE is a SAR or a SUSAR, and therefore a response to antiretroviral therapy or other medication, the MRC CTU will report the SAR or SUSAR to the relevant drug manufacturer with whom they have a contract.

3. Follow-up: The subject must be followed-up until clinical recovery is complete and laboratory results have returned to normal, or until the event has stabilised. Follow-up may continue after completion of protocol treatment if necessary. Follow-up information is noted on another SAE form by ticking the box marked 'follow-up' and faxing to the ARROW Trials Centre as information becomes available. Extra, annotated information and/or copies of test results may be provided separately.

4. Resolution: If clinical recovery is complete and laboratory results have returned to normal, a "resolution" SAE form should be completed.

5. The patient's name **should not** be used on any correspondence. The patient **must** be identified by trial number, date of birth and initials only.

8.0 Drug Regimens

8.1 Antiretroviral Therapy

Table 1 First and second-line drug regimens for ARROW (For details see Appendix 1.0,page 61)

First-line treatment (up to 36 weeks)	First-line treatment (after 36 weeks)	Second-line treatment
NNRTI + ABC+3TC	NNRTI + ABC+3TC	2NRTIs + boosted PI*
NNRTI + ZDV+ ABC+3TC	NNRTI + ABC+3TC	2NRTIs + boosted PI*
NNRTI + ZDV+ ABC+3TC	ZDV+ ABC+3TC	2NRTIs + boosted PI* or NNRTI + boosted PI*

* The boosted PI could be Saquinavir/Ritonavir or Lopinavir/ Ritonavir or a new PI e.g. TMC114/ Ritonavir

8.1.1 *First-line antiretroviral drugs*

All children will receive a triple combination of ART, including ABC + 3TC, after 36 weeks. During the first 36 weeks of treatment, children in the induction-maintenance arms will receive an additional fourth drug. Children with any contra-indications to receipt of NVP (e.g. those on concomitant anti-tuberculosis therapy) should receive EFV as the NNRTI. Children under 3 years old on anti-tuberculosis medication should not be enrolled, and ART should be initiated according to national guidelines. Children under 6 months of age who received NVP in the perinatal period as part of PMTCT or breast feeding should not be enrolled.

8.1.2 *Second-line antiretroviral drugs*

Second-line therapy will be 2NRTIs + a boosted PI or NNRTI + a boosted PI. The choice of second-line will be determined by the ART drugs already received (see Table 1 above), but taking into account any first-line substitutions, e.g. for toxicity. For adolescents, TDF may also be available as an option for second-line therapy should there be particular contra-indications to use of a PI.

Upon failure of a second-line regimen, the best possible combination will be selected from first and second-line drugs for the remaining duration of the trial. After trial completion follow-up healthcare will be provided by the national health systems.

8.1.3 *Provision of trial drugs*

Children or their carers will be provided with a 6-weekly (4-weekly before week 48) supply of drugs throughout the trial (sufficient to last until their next clinic visit) and will be requested to return all empty bottles and to bring any bottles in use to the follow-up clinic. Drugs will be provided for the trial as liquids, tablets and scored tablets (combivir, 3TC, ABC) and will follow guidelines for dosing according to simplified WHO weight band tables.

On no account should any drug assigned to a patient be used by anyone else. Unused drug must be returned to the site if a patient withdraws from treatment.

All drug dispensed and returned to the site should be documented on a treatment log for each patient. At each site, a named person (trial pharmacist or research nurse) will be required to maintain complete records of all study medication dispensed. The procedures to be followed will adhere to the Good Clinical Practices (GCP) guidelines on drug accountability. MRC CTU will monitor drug accountability at site visits.

8.1.4 *Modification of therapy for toxicity*

Alternate explanations for clinical or laboratory abnormalities that may at first appear to be related to a specific drug should be sought. The use of reduced doses of the drug is discouraged.

Management of any other adverse events/toxicity should be done as per the following criteria:

Grade 1:

Continue study drugs

Routine monitoring

Grade 2:

Continue study drugs

Monitor closely with more frequent visits (i.e. every two weeks) and for LCM arm consider more frequent laboratory assessments

Work-up to exclude other causes

Grade 3 or 4:

LCM arm: if relevant, obtain repeat confirmatory laboratory results within 72 hours

CDM arm: if clinically indicated, laboratory results can be requested; if relevant, obtain repeat confirmatory laboratory results within 72 hours

Continue study drugs pending receipt of the confirmatory laboratory tests/repeat observations, unless immediate need to substitute

Work-up to exclude other causes

Following confirmation of toxicity, and lack of other cause data:

- If not too sick substitute immediately
- Otherwise, stop all drugs (stagger stop NNRTIs) and restart with substituted drugs when better

Note that staggered stop is necessary for NNRTIs because these drugs have long half lives and low genetic barriers to resistance. Therefore stopping NNRTIs + 2NRTIs together will lead to a period of time where only NNRTI remains in the child's blood, with the potential for development of resistance. Therefore, if a child is taking an NNRTI containing regimen, stop the NNRTI immediately and continue the remaining drugs for a further 10 days for NVP and 14 days for EFV. Stop all remaining drugs together after 10 or 14 days respectively.

For severe reactions where the cause may be attributed to one or more trial drugs, all drugs should be stopped temporarily and may be restarted if the symptoms resolve and this is appropriate (stagger stop for NNRTIs wherever possible).

If on 4 drugs, the principle will be to stop the causal drug and continue on 3 drugs. If a child does not tolerate an individual drug/drug formulation, an alternative drug may be substituted if this is considered appropriate by the investigator (see Table 2, below) and other drugs restarted. Wherever possible, substitutes should be made within class. ZDV and 3TC will be available as separate drugs for children who need to stop one drug for toxicity or intolerance. See also Appendix 2.0, page 64.

Table 2 Guidelines for Substituting for Toxicity

	**Substitution***	
**Event**	**if on 3 drugs**	**if on 4 drugs**
ZDV toxicity	Substitute ZDV with d4T or ddI or NVP or EFV (>3 years)	Stop ZDV
3TC toxicity	Substitute 3TC with ZDV or ddI or NVP or EFV (>3 years)	Stop 3TC
ABC toxicity	Substitute ABC with ZDV or NVP or EFV (>3 years) or TDF (adolescents)	Stop ABC
NVP toxicity	Substitute NVP with EFV (>3 years) or ZDV or TDF (adolescents) or Kaletra	Stop NVP

* Choice of substitution depends upon:

(i) other drugs being taken (ii) available formulations (e.g. syrups/size of tablet).

If possible, preferred substitution would be within same class.

NOTE: In the event of discontinuation of ABC for any reason, re-initiation of this drug should be undertaken with caution. Health care providers should obtain a complete history of the events surrounding the discontinuation of ABC. If there are any symptoms consistent with a hypersensitivity reaction, ABC should not be reinitiated. If there is no evidence of a prior reaction, the subject may restart treatment with ABC. The subject and health care provider should be aware of the possibility of a rapid-onset hypersensitivity reaction upon re-initiation of ABC, which may be life-threatening, and the subject should be able to, if necessary, receive prompt medical evaluation (Refer to Appendix 5.1, page 87)

8.1.5 *Management of potential ABC or NVP reactions when children are taking both*

If children are taking both ABC and NVP it may be more difficult to isolate the causal agent for a suspected severe reaction as both drugs have overlapping signs/symptoms. Clinicians should first check Liver Function Tests (LFTs) as raised LFTs would suggest that a reaction

is more likely to be due to NVP. However, regardless of LFT levels the first action in the case of a severe reaction should be to stop NVP and monitor the child.

If a severe reaction occurs on ***3TC+ZDV+ABC+NVP/EFV***: if the reaction subsides after stopping NVP, continue 3TC+ZDV+ABC. If the reaction continues stop ABC immediately (but continue 3TC+ZDV to provide 14 days stagger stop from time of stopping NVP); after it has subsided restart NVP (with dose escalation) +3TC+ZDV.

If a severe reaction occurs on ***3TC+ABC+NVP***: if the reaction subsides after stopping NVP, substitute for NVP according to Table 2, above. If the reaction continues, stop 3TC+ABC immediately: after it has subsided, restart NVP (with dose escalation) +3TC+ZDV.

8.2 Prophylaxis against Opportunistic Infections

Decisions on prophylaxis strategies will be made taking into account national guidelines. All prophylaxis therapy should be recorded. Unless otherwise contraindicated or randomised to stop cotrimoxazole within the ARROW study, all children should continue to receive cotrimoxazole prophylaxis.

8.3 Medications not permitted/ Precautions

Children should not be co-enrolled in other ART trials or receive other ART outside the trial. Children and their carers will be encouraged to seek advice prior to taking any other medication. See Appendix 1.0, page 61 for details of drug interactions.

8.4 Data on concomitant medications

At each visit, information on other medications, including start dates, and reason(s) for taking should be documented on follow-up forms.

9.0 Management of the Trial

9.1 Trial Steering Committee

The trial will be managed by a Trial Steering Committee (TSC) with an independent chairperson, a majority of independent members and one Principal Investigator or key investigator from each site and from MRC CTU.

9.2 Trial Management Group

A Trial Management Group (TMG) of principal and key investigators from each site, as well as members from MRC CTU will communicate regularly to ensure that the trial is proceeding well across all four sites. Local Trial Management Committees composed of investigators at each site will meet regularly. There will be regular teleconferences to ensure good communication across sites and with MRC CTU.

9.3 Data Management and Monitoring

A Data Management Group (DMG) will be set up with data management, computing and statistician members from each site and the MRC CTU, chaired by the Trial Statistician. This Committee will be responsible for setting up the databases at each site and for co-ordination of timely merging of data from each site at MRC CTU, where the central database will be held. The committee will be responsible for ensuring that the system for data collection is working consistently across the sites, for developing the trial analysis plan and 'shell' tables to be provided to the independent Data Monitoring Committee, and for making decisions about analyses.

Each site will be responsible for maintaining its own database and for timely (twice monthly) transfer of checked data to the MRC CTU for merging of data with those from the other sites. Staff from MRC CTU will visit clinical sites to validate and monitor data and this may also be done across sites (e.g. a data manager from Zimbabwe may visit Uganda), under the oversight of the Data Management Committee. The clinical investigators and participants, by giving consent, agree that within the host country's Data Protection Law, the MRC CTU may consult and /or copy source records (clinical notes, laboratory values) in order to do this. Such information will be treated as strictly confidential and will in no circumstances be made publicly available. The monitoring will adhere to MRC Good Clinical Practice guidelines (based on ICH guidelines). The following data should be verifiable from source documents: all signed consent forms; dates of visits including laboratory results; eligibility and baseline values for all children; all clinical endpoints; all serious/severe adverse events; an ongoing random 5% sample of routine patient clinical and laboratory data; drug compliance; dates drug dispensed and (if necessary) drugs returned; pharmacy/clinic drug logs; concomitant medication.

9.4 Data Monitoring Committee

An independent Data Monitoring Committee (DMC) will be established and will monitor all aspects of the trial, including all four randomisations (LCM/CDM, induction-maintenance, stop/continue cotrimoxazole prophylaxis, once/twice daily lamivudine+abacavir). The DMC will consider findings from any other relevant studies and review trial data on recruitment, safety, adherence to randomised strategies and efficacy, in strict confidence approximately every 6-12 months. The DMC will report to the ARROW Trial Steering Committee and to the Ethics Committee in each country, if in their view the data provide proof beyond reasonable doubt that one of the allocated strategies is better than its comparator in terms of a difference of clinically significant magnitude in a primary outcome. The guiding statistical criteria for "proof beyond reasonable doubt" is a Haybittle-Peto type rule based on the 99.9% confidence interval of the relative hazard of disease progression in each interim analysis, but the DMC will also consider clinical criteria. The ARROW Trial Steering Committee will then decide whether to amend or stop the trial before the end of the planned follow-up.

9.4.1 Formal primary endpoint analysis for once versus twice daily lamivudine+abacavir

The key primary endpoint for the comparison of once versus twice daily lamivudine+abacavir is HIV-1 RNA viral load suppression at 48 weeks, similarly to the adult trials which led to licensing of this once-daily combination in adults. Because any fragility of once daily lamivudine+abacavir weight-band based dosing would be expected to become more apparent over time, and because HIV-1 RNA testing is not being performed in real-time in ARROW,

children will be followed on their randomised strategy for at least 96 weeks. When all randomised children have completed 48 weeks of follow-up (estimate November 2010), retrospective viral load testing will be performed on the batched 48 week samples. When results of this analysis are available (estimate June 2011 at the earliest), over half the children randomised to once versus twice daily lamivudine+abacavir will have reached 96 weeks, and therefore following the children on randomised strategy until 96 weeks will be a small amount of additional time. A similar procedure for retrospective analysis will be followed for week 96 samples. All viral load results will be returned for individual children when they exit the ARROW trial in April 2012. If analysis of week 48 data suggest inferiority of once daily dosing then all children will be moved back to twice daily dosing as soon as the information is available.

9.5 Endpoint Review Committee

An Endpoint Review Committee will be appointed whose remit will be to determine the validity of potential clinical endpoints in terms of meeting the standard criteria, as defined by the protocol (Appendix 3.0, page 80). It will have an independent Chair and will include Project Leaders from each site as well as other independent clinicians. No member will review endpoints from their own site. Terms of reference for the Endpoint Review Committee will be drawn up.

10.0 Statistics

10.1 Monitoring Practice: LCM versus CDM

The aim is to recruit 1200 children (800 from the 3 sites in Uganda and 400 from Zimbabwe) during the first 18 months. Children will be allocated in a 1:1 ratio to LCM or CDM.

The estimation of the sample size in protocol 1.0-2.0 is based on the following assumptions:

1. **15%** of children receiving standard triple drug ART progress to new WHO stage 4 disease or death per year in the LCM arm (representing >80% reduction in progression under ART - a similar progression rate as assumed for progression to WHO stage 4 disease or death per year in the LCM arm in adult DART)
2. Progression rates to a new WHO stage 4 disease or death are reduced to **10%** per year in the best of the induction maintenance arms (see below), leading to an overall rate of progression to new WHO stage 4 or death of **12.5%**
3. Recruitment is over 1.5 years and follow-up for a minimum further 3.5 years.
4. Cumulative loss to follow-up is 10% at 5 years.

With these assumptions, at least 90% power and one-sided alpha=0.05, 1145 children would be required in the CDM/LCM comparison to exclude an increase in progression rate of 3.75% from 12.5% to 16.25% per year in the CDM arm (upper 95% confidence limit of LCM: CDM hazard ratio 1.30). Note: if there is no difference between standard and induction maintenance arms, the trial still retains at least 80% power to exclude an increase in progression rate of 3.75% per annum, but from a higher overall rate of 15% to 18.75% (upper 95% confidence limit of LCM: CDM hazard ratio 1.25).

During 2009, it became clear that the rate of progression to a new stage WHO 4 disease or death was far smaller than original anticipated (and also lower than at a similar stage in the adult DART trial) at ~**2.5%** per annum (compared to originally estimated overall rates of

12.5%, or 15% if no impact of induction maintenance). Recruitment ended in 2008, so there was no opportunity to increase the sample size. However, progression to a new stage WHO 4 disease or death is still judged to be the most relevant clinical endpoint for children. Therefore a revised non-inferiority margin has been set for new WHO stage 4 disease or death (see below) which is retained as the primary endpoint, and the original less wide non-inferiority margin has been retained for a new secondary endpoint of new WHO stage 3 or 4 event or death excluding oral/oesophageal candida (which is non-fatal).

As all event rates are small, the non-inferiority margin is set for **the absolute increase in rate of events per annum**, which is converted into a relative risk for presentation (ie the relative risk increase is not the primary non-inferiority measure as this depends strongly on the underlying event rates which are low).

Non-inferiority margin for progression to a new WHO stage 4 event or death

1. **3%** of children receiving standard triple drug ART progress to new WHO stage 4 disease or death per year in the LCM arm
2. Progression rates to a new WHO stage 4 disease or death are reduced to **2%** per year in the best of the induction maintenance arms (assuming a 67% reduction in event rate as per original sample size calculation), leading to an overall rate of progression to new WHO stage 4 or death of **2.5%**
3. Recruitment is over 1.5 years and follow-up for a minimum further 3.5 years.
4. Cumulative loss to follow-up is 10% at 5 years.

With these assumptions, at least 90% power and one-sided alpha=0.05, 1160 children would be required in the CDM/LCM comparison to exclude an increase in progression rate of **1.6%** from 2.5% to 4.1% per year in the CDM arm (upper 95% confidence limit of LCM: CDM hazard ratio 1.64). Note: if there is no difference between standard and induction maintenance arms, the trial still retains at least 80% power to exclude an increase in progression rate of **1.7%** per annum, but from a higher overall rate of 3% to 4.7% (upper 95% confidence limit of LCM:CDM hazard ratio 1.58). If the rate is actually lower, the trial still retains at least 80% power to exclude increases in progression rate of **1.6%** per annum, although this corresponds to a greater relative increase (eg if the overall rate is 1.5%, 1155 children provides at least 80% power to exclude an increase in progression rate of 1.3% (from 1.5% to 2.8%, upper 95% confidence limit of LCM:CDM hazard ratio 1.85)

Non-inferiority margin for progression to a new WHO stage 3 or 4 event or death

1. **9%** of children receiving standard triple drug ART progress to new WHO stage **3 or** 4 disease or death (excluding oral/oesophageal candida) per year in the LCM arm
2. Progression rates to a new WHO stage **3 or** 4 disease or death (excluding oral/oesophageal candida) are reduced to **6%** per year in the best of the induction maintenance arms (assuming a 67% reduction in event rate as per original sample size calculation), leading to an overall rate of progression to new WHO stage **3 or** 4 or death **of 7.5%**
3. Recruitment is over 1.5 years and follow-up for a minimum further 3.5 years.
4. Cumulative loss to follow-up is 10% at 5 years.

With these assumptions, at least 90% power and one-sided alpha=0.05, 1163 children would be required in the CDM/LCM comparison to exclude an increase in progression rate of **2.8%** from 7.5% to 10.3% per year in the CDM arm (upper 95% confidence limit of LCM: CDM hazard ratio 1.37). Note: if there is no difference between standard and induction

maintenance arms, the trial still retains at least 80% power to exclude an increase in progression rate of **3%** per annum, but from a higher overall rate of 9% to 12% (upper 95% confidence limit of LCM: CDM hazard ratio 1.34).

10.2 ART strategies for first line therapy

10.2.1 CD4 (primary endpoint)

Assuming a standard deviation for the change in CD4 percentage from baseline to 72 weeks of 10% (slightly higher than that observed in the PENTA 5 trial, [19]) 1200 children would provide at least 80% power to detect a difference in change in CD4% from baseline of more than 2.5% across the 3 groups (F-test with 2-sided alpha=0.05) assuming 20% missing data (loss to follow-up during the first year plus failure to attend the week 72 visit/missing sample).

10.2.2 Clinical

With the above assumptions, at least 80% power and two-sided alpha=0.05, 1127 children would be required in the comparison of first-line therapies to detect a decrease in progression rate of 5% from 15% to 10% per year between any 2 of 3 arms [Arm A, Arm B and Arm C] (HR=0.67, based on 3 pair-wise tests at two-sided alpha=0.01 to adjust for multiple comparisons). From a lower disease progression rate, 1200 children would be required to detect a decrease in progression rate of 50% from 3% to 1.5% per year between any 2 of 3 arms (HR=0.50, 3 pairwise tests at two-sided alpha=0.01).

10.2 Analysis Plan

The primary analysis will compare LCM with CDM, and induction-maintenance strategies in terms of:

a) Progression to a new HIV stage 4 event or death and
b) Any grade 3 or 4 adverse events which are not HIV-related only

Time-to-event methods (Kaplan-Meier plots, stratified log rank test and Cox proportional hazard regression) will be used for these comparisons. The frequency of all grade 3 or 4 adverse events will also be tabulated by body systems and randomised group and the groups will be compared using the X^2 test. Wilcoxon's rank-sum test will be used to compare median CD4 cell counts at 72 and 144 weeks in the different allocated strategies.

10.3 Once versus twice daily lamivudine+abacavir

The aim is to recruit all eligible ARROW children from the ARROW centres in Uganda (3) and Zimbabwe (1) (see section 6.4.3, p37). Children will be allocated in a 1:1 ratio to once or twice daily lamivudine+abacavir.

The estimation of the sample size is based on the following assumptions:

1. 70% children have HIV RNA <50 copies/ml at week 48 on twice-daily dosing
2. 15% of randomised children have missing sample for HIV-1 RNA testing at week 48, due to missing sample, missed visit, death or loss to follow-up.

With these assumptions, at least 90% power and two-sided alpha=0.05, at least 934 children would be required to exclude a 10% lower suppression rate in the once daily group (lower 95% confidence limit of difference between once and twice daily -10%). Under similar

assumptions and assuming that the standard deviation for the change in CD4 cell count from randomisation to 96 weeks is 100 cells/mm^3 (similar to that in children in the PENTA randomised trials), at least 1000 children would be required to exclude a 22 cell/mm^3 smaller change in CD4 increase from 0 to 48/96 weeks in the once daily group (lower 95% confidence limit of difference between once and twice daily -22 cells/mm^3).

During accrual to this randomisation during 2009-2010, more children than initially projected were found to be ineligible due to already receiving full adult dose Kivexa (lamivudine+abacavir) once daily in the highest weight-bands. Therefore recruitment to the original target of 1000 could not be achieved. The revised target of 630 children is based on a 12% non-inferiority margin (also recommended by FDA): at least 631 children would be required to exclude a **12%** lower suppression rate in the once daily group with at least 90% power and two-sided alpha=0.05 (lower 95% confidence limit of difference between once and twice daily -12%). Of note, the revised sample size of 630 children retains at least 80% (rather than 90%) power to exclude a 10% (rather than 12%) lower suppression rate in the once daily group with **one**-sided alpha=0.05 (lower 90% confidence limit of difference between once and twice daily -10%). Under similar assumptions as above, at least 619 children would be required to exclude a **28** cell/mm^3 smaller change in CD4 increase from 0 to 48/96 weeks in the once daily group (lower 95% confidence limit of difference between once and twice daily -28 cells/mm^3).

10.4 Stop versus continue cotrimoxazole prophlyaxis

The aim is to recruit all eligible ARROW children from the ARROW centres in Uganda (3) and Zimbabwe (1) (see section 6.5.3, p37). Children will be allocated in a 1:1 ratio to stop or continue daily cotrimoxazole prophylaxis.

The estimation of the sample size is based on the following assumptions:

1. 5% of children receiving daily cotrimoxazole prophylaxis have a new hospitalisation or death per year (similar event rate to that seen overall in the adult DART trial)
2. Recruitment starts 1 July 2009 with 10% children (those already on ART for >96 weeks) entering immediately, and then the remaining children recruited over the following 15 months as they reach 96 weeks on ART. Follow-up is until March 2012.
3. Cumulative loss to follow-up at March 2012 is 10%.

With these assumptions, at least 80% power and one-sided alpha=0.05, 947 children would be required in the stop/continue cotrimoxazole prophylaxis comparison to exclude an increase in hospitalisation/death rate of 3% from 5% to 8% per year in the stop cotrimoxazole arm (upper 95% confidence limit of stop:continue hazard ratio 1.6). If more children are randomised, 1104 children would provide at least 80% power to exclude an increase in hospitalisation/death rate of 2.6% from 5% to 7.6% per year in the stop cotrimoxazole arm (upper 95% confidence limit of stop:continue hazard ratio 1.55).

11.0 Regulatory/ Ethics Approval

This document, along with any subsequent modifications and with the sample informed consent documents will be reviewed by the Ethics Committee from each participating site as well as by the UCL Research Ethics Committees in the UK.

Regulatory approval for conduct of the trial and use of antiretrovirals in the trial will be obtained.

12.0 Confidentiality

A unique trial number will identify all laboratory specimens, case record forms, and other records and no names will be used, in order to maintain confidentiality. All records will be kept in locked locations. Clinical information will not be released without written permission, except as necessary for monitoring by the trial monitors.

13.0 Publication

The ARROW Trial Management Group will develop guidelines for the preparation of papers (including abstracts) for presentation at national and international meetings, as well as the preparation of manuscripts for peer-reviewed publication. Any publication or presentation during the active phase of the study must have prior approval of the TSC. The TSC will define the strategy for, and resolve any problems of authorship and maintain the quality of publications. All publications will acknowledge appropriate funding sources. The ARROW TSC is the custodian of the data and specimens generated from the ARROW trial; ARROW trial data are not the property of individual participating investigators or health care facilities where the data were generated.

14.0 References

1. UNAIDS/WHO AIDS epidemic update: December 2005; http://www.unaids.org/epi/2005/doc/report_pdf.asp
2. Detels R, Munoz A, McFarlane G, *et al*. Effectiveness of potent antiretroviral therapy on time to AIDS and death in men with known HIV infection duration. *JAMA* 1998;280:1497-503.
3. Gibb DM, Duong T, Tookey PA *et al.* on behalf of the National Study of HIV in Pregnancy and Childhood (NSHPC) and of the Collaborative HIV Paediatric Study (CHIPS). Decline in mortality, AIDS and hospital admissions in perinatally HIV-1 infected children in the UK and Ireland. *BMJ* 2003; 327:1019-1025.4.
4. Treating 3 million by 2005 *Making it Happen* The WHO Strategy, The WHO and UNAIDS global initiative to provide antiretroviral therapy to 3 million people with HIV/AIDS in developing countries by the end of 2005. World Health Organization, 2003 ISBN 92 4 159 1129;http://www.who.int/3by5/en/
5. The “3 by 5” Initiative progress report, June 2005, WHO and UNAIDS.
6. Koenig SP, Kuritzkes DR, Hirsch MS, Leandre F, Mukherjee JS, Farmer PE, del Rio C. Monitoring HIV treatment in developing countries. *BMJ* 2006; 332:602-4.
7. DART Virology Group and Trial Team. Virological response to a triple nucleoside/nucleotide analogue regimen over 48 weeks in HIV-1-infected adults in Africa. *AIDS.* 2006;20:1391-9.
8. Chintu C, Bhat GJ, Walker AS, Mulenga V, Sinyinza F, Lishimpi K, Farrelly L, Kaganson N, Zumla A, Gillespie S, Nunn AJ, Gibb DM, on behalf of the CHAP Trial team. A Randomized Placebo-Controlled Trial Of Cotrimoxazole As Prophylaxis Against Opportunistic Infections In HIV-Infected Zambian children: the CHAP (Children With HIV Antibiotic Prophylaxis) Trial. *Lancet* 2004; 364:1865-71
9. Antiretroviral therapy for HIV infection in infants and children towards universal access: Recommendations for a public health approach (2007 Revision). *World Health Organization* 2007.
10. Dunn D and the HIV Paediatric Prognostic Markers Collaborative Study Group. Short-term risk of disease progression in HIV-1-infected children receiving no antiretroviral therapy or zidovudine monotherapy: a meta-analysis. *Lancet* 2002; 362:1605-11.

11. De Rossi A, Walker AS, Klein N, et al. Increased thymic output after initiation of antiretroviral therapy in human immunodeficiency virus type 1-infected children in the Paediatric European Network for Treatment of AIDS (PENTA) 5 Trial. *J Infect Dis* 2002; 186:312-20.

12. Diaz C, Hanson C, Cooper ER, Read JS, Watson J, Mendez HA, Pitt J, Rich K, Smeriglio V, Lew JF. Disease progression in a cohort of infants with vertically acquired HIV infection observed from birth: the Women and Infants Transmission Study (WITS). *J Acquir Immune Defic Syndr Hum Retrovirol*. 1998;18:221-8.

13. Newell ML, Coovadia H, Cortina-Borja M, Rollins N, Gaillard P, Dabis F; Ghent International AIDS Society (IAS) Working Group on HIV Infection in Women and Children. Mortality of infected and uninfected infants born to HIV-infected mothers in Africa: a pooled analysis. *Lancet*. 2004; 364:1236-43.

14. Tovo PA, de Martino M, Gabiano C, Cappello N, D'Elia R, Loy A, Plebani A, Zuccotti GV, Dallacasa P, Ferraris G, et al. Prognostic factors and survival in children with perinatal HIV-1 infection. The Italian Register for HIV Infections in Children. *Lancet*. 1992 ;339:1249-53.

15. Ziagen (abacavir sulphate): Prescribing information. GlaxoSmithKline, 2002.

16. Lockman S, Shapiro RL, Smeaton LM, Wester C, Thior I, Stevens L, Chand F, Makhema J, Moffat C, Asmelash A, Ndase P, Arimi P, van Widenfelt E, Mazhani L, Novitsky V, Lagakos S, Essex M. Response to antiretroviral therapy after a single, peripartum dose of nevirapine. *N Engl J Med.*. 2007; 356:135-47

17. Violari A, Cotton M, Gibb D. Antiretroviral therapy initiated before 12 weeks of age reduces early mortality in young HIV-infected infants: evidence from the Children with HIV Early Antiretroviral Therapy (CHER) Study [abstract WESS103]. 4th IAS Conference on HIV Pathogenesis, Treatment and Prevention 2007, Sydney, Australia.

18. Mphatswe W, Blanckenberg N, Tudor-Williams G, Prendergast A, Thobakgale C, Mkhwanazi N, McCarthy N, Walker BD, Kiepiela P, Goulder P. High frequency of rapid immunological progression in African infants infected in the era of perinatal HIV prophylaxis.*AIDS*. 2007 ;21:1253-61.

19. Wade AM. Ades AE. Age-related reference ranges: significance tests for models and confidence intervals for centiles. *Statistics in Medicine* 13:2359-67, 1994.

20. HIV Paediatric Prognostic Markers Collaborative Study Group. Predictive value of absolute CD4 count for disease progression in untreated HIV-1-infected children. *AIDS* 2006; 20:1289-94.

21. Paediatric European Network for Treatment of AIDS Comparison of dual nucleoside-analogue reverse transcriptase inhibitor regimens (ZDV+3TC or ZDV+ABC or 3TC+ABC) with and without a protease inhibitor (Nelfinavir) in previously untreated HIV-infected children: The PENTA 5 Trial. *Lancet* 2002; 359: 733-740.

22. Gibb DM, Green H, Saidi Y, Pillay D, Compagnucci A, Harper L, Walker AS, Castelli-Gattinara G, Della Negra M, Levy J, Candeias F, Butler K, Feiterna-Sperling C, Wintergerst U, Giaquinto C on behalf of PENTA 5. 3TC+ABC maintains virological superiority over ZDV+3TC and ZDV+ABC beyond 5 years in children: the PENTA 5 trial. *AIDS* 2007; 21:947-955

23. Kiser J, Rutstein R, Aldrovandi G, Samson P, Graham B, Schnittman S, Smith M, Mofenson L, Fletcher C; PACTG 1020 Study Team. Pharmacokinetics of Atazanavir/Ritonavir in HIV-infected Infants, Children, and Adolescents: PACTG 1020A**.** 12th Conference on Retroviruses and Opportunistic Infections, February 2005, Boston, USA.

24. Faye A, Bertone C, Teglas JP, Chaix ML, Douard D, Firtion G, Thuret I, Dollfus C, Monpoux F, Floch C, Nicolas J, Vilmer E, Rouzioux C, Mayaux MJ, Blanche S; French Perinatal Study. Early multitherapy including a protease inhibitor for human immunodeficiency virus type 1-infected infants. *Pediatr Infect Dis J* 2002; 21:518-25.

25. Aboulker J-P, Babiker A, Chaix ML, Compagnucci A, Darbyshire JH, Debré M, Faye A, Giaquinto C, Gibb DM, Harper L, Saidi Y, Walker AS; Paediatric European Network for Treatment of AIDS. 72-week follow-up of HAART started in infants aged less than 3 months: CD4, viral load and drug resistance outcomes in the PENTA 7 study. *AIDS* 2004; 18:237-45.

26. Luzuriaga K, McManus M, Mofenson L, Britto P, Graham B, Sullivan JL; PACTG 356 Investigators. A trial of three antiretroviral regimens in HIV-1-infected children. *N Engl J Med* 2004; 350:2471-80.

27. Walker AS, Doerholt K, Sharland M, Gibb DM; Collaborative HIV Paediatric Study (CHIPS) Steering Committee. Response to highly active antiretroviral therapy varies with age: the UK and Ireland Collaborative HIV Paediatric Study. *AIDS* 2004; 18:1915-24.

28. Van Rossum AM, Fraaij PL, de Groot R. Efficacy of highly active antiretroviral therapy in HIV-1 infected children. *Lancet Infect Dis*. 2002; 2: 93-102.

29. Bartlett JA, Fath MJ, Demasi R, Hermes A, Quinn J, Mondou E, Rousseau F. An updated systematic overview of triple combination therapy in antiretroviral-naive HIV-infected adults. *AIDS* 2006; 20:2051-64

30. Mittler J, Essunger P, Yuen GJ, Clendeninn N, Markowitz M, Perelson AS. Short-term measures of relative efficacy predict longer-term reductions in human immunodeficiency virus type 1 RNA levels following nelfinavir monotherapy. *Antimicrob Agents Chemother.* 2001;45:1438-43.

31. Orkin C, Stebbing J, Nelson M, Bower M, Johnson M, Mandalia S, Jones R, Moyle G, Fisher M, Gazzard B. A randomized study comparing a three- and four-drug HAART regimen in first-line therapy (QUAD study). *J Antimicrob Chemother.* 2005; 55:246-51.

32. Moyle G, Higgs C, Teague A, Mandalia S, Nelson M, Johnson M, Fisher M, Gazzard B. An open-label, randomized comparative pilot study of a single-class quadruple therapy regimen versus a 2-class triple therapy regimen for individuals initiating antiretroviral therapy. *Antivir Ther.* 2006; 11:73-8.

33. Cowper A, Lyall H, Walker S, Walters S, Rosenfeldt V, Feiterna-Sperling C, Segal S, Head S, Tudor-Williams G. A 4-drug, protease-inhibitor sparing, combination antiretroviral therapy for infants with advanced HIV infection. 7th International Congress on Drug Therapy in HIV Infection, November 2004, Glasgow, UK.

34. Doerholt K, Duong T, Tookey P, Butler K, Lyall H, Sharland M, Novelli V, Riordan A, Dunn D, Walker AS, Gibb DM and the Collaborative HIV Paediatric Study (CHIPS) . Outcomes for Human Immunodeficiency Virus-1-Infected Infants in the United Kingdom and Republic of Ireland in the Era of Effective Antiretroviral Therapy *Pediatr Infect Dis J* 2006; 25:1-7.

35. Bergshoeff A, Burger D, Verweij C, Farrelly L, Flynn J, LeProvost M, Walker AS, Novelli V, Lyall H, Khoo S, Gibb DM on behalf of the PENTA 13 study group. Pharmacokinetics of once versus twice daily lamivudine and abacavir. Simplification of combination treatment in HIV-1 infected children (Penta 13). *Antivir Ther* 2005;10: 239-246.

36. Havlir D, Strain M, Clerici M, Trabaltoni D, Ferrante P, Wong J. Productive infection maintains a dynamic steady state of residual viraemia in HIV-1-infected persons treated with suppressive antiretroviral therapy for five years. *Antiretroviral Therapy* 2002; 7 (suppl 1): S54.

37. De Wit S, Johnson M, Gazzard B, Bergman JF, Reynes J, Estrada V, Castagna A, Rockstroh J, AZL30004 study team. Randomised Comparison of Maintenance Therapy with Trizivir (TZV) or TZV + Efavirenz (EFV) vs TZV in naïve HIV-1 infected Subjects: TIME Study (AZL30004). 7th International Congress on Drug Therapy in HIV Infection, November 2004, Glasgow, UK.

38. Markowitz M, Hill-Zabala C, Lang J, DeJesus E, Slater L, Liao Q, Lanier E R, Davis E A, Shaefer M. for the ESS40013 Study Team. Induction with abacavir/lamivudine/zidovudine plus efavirenz for 48 weeks followed by 48-week maintenance with abacavir/lamivudine/zidovudine alone in antiretroviral-naive HIV-1-infected patients. *J Acquir Immune Defic Syndr.* 2005; 39:257-64.

39. Asboe D, Williams IG, Goodall RL, Darbyshire JH, Hooker MH, Babiker AG; Forte Steering Committee. A virological benefit from an induction/maintenance strategy: the Forte trial *Antivir Ther.* 2007;12:47-54.

40. Gulick RM, Ribaudo HJ, Shikuma CM, Lustgarten S, Squires KE, Meyer WA 3rd, Acosta EP, Schackman BR, Pilcher CD, Murphy RL, Maher WE, Witt MD, Reichman RC, Snyder S, Klingman KL, Kuritzkes DR; AIDS Clinical Trials Group Study A5095 Team. Triple-nucleoside regimens versus efavirenz-containing regimens for the initial treatment of HIV-1 infection. *N Engl J Med.* 2004; 350:1850-61.

41. Baylor M, Ayime O, Truffa M, Denson A, Johann-Liang R. Hepatotoxicity associated with nevirapine use in children. 12th Conference on Retroviruses and Opportunistic Infections, February 2005, Boston, USA.

42. Ellis JC, L'homme RFA, Ewings FM, Mulenga V, Bell F, Chileshe R, Molyneux E, Abernethy J, van Oosterhout JJG, Chintu C, Walker AS, Gibb DM, Burger DM. Nevirapine concentrations in HIV-infected children treated with divided fixed-dose combination antiretroviral tablets in Malawi and Zambia *Antiviral Therapy* 2007; 12: 253-260

43. DART trial team. Twenty-four-week safety and tolerability of nevirapine vs. abacavir in combination with zidovudine/lamivudine as first-line antiretroviral therapy: a randomized double-blind trial (NORA). *Trop Med Int Health.* 2008; 13:6-16.

44. NIH Division of Microbiology and Infectious Diseases (DMID) Pediatric toxicity tables, November 2007: http://www3.niaid.nih.gov/research/resources/DMIDClinRsrch/toxtables

45. Centers for Disease Control and Prevention (1994). 1994 Revised Classification system for human immunodeficiency virus infection in children less than 13 years of age. MMWR report 43: 1-17

46. Centers for Disease Control and Prevention (2000). CDC Growth Charts: United States. Advance Data number 314; December 4, 2000 (revised)

47. Campbell J, Moore D,Degerman R, Kaharuza F, Were W, Muramuzi E, mermin J, Tappero J. HIV-infected Ugandans on HAART with CD4 counts >200Cells/mm3 who discontinue Cotrimoxazole have increased risk of malaria and Diarrhoea. 16th Conference on Retroviruses and Opportunistic Infections, February 2009, Montréal, Canada.

48. Gasasira A, Havlir D, Achan J, E Charlebois, T Ruel, P Rosenthal, T Sandison, G Dorsey, and M Kamya. Prophylactic effect of Trimethoprim-Sulfamethoxazole on malaria in HIV-infected and –exposed Ugandan children living in settings of high antifolate resistance. 16th Conference on Retroviruses and Opportunistic Infections, February 2009, Montréal, Canada.
AIDS 2007; 21:2059-2066

49. Anglaret X, Chene G, Attia A, Toure S, Lafont S, Combe P, et al. Early chemoprophylaxis with trimethoprim-sulphamethoxazole for HIV-1-infected adults in Abidjan, Cote d'Ivoire: a randomised trial. Cotrimo-CI Study Group. Lancet. 1999 May 1;353(9163):1463-8.

50. Badri M, Maartens G, Wood R, Ehrlich R. Co-trimoxazole in HIV-1 infection. Lancet. 1999 Jul 24;354 (9175):334-5.

51. Chintu C, Bhat GJ, Walker AS, Mulenga V, Sinyinza F, Lishimpi K, et al. Co-trimoxazole as prophylaxis against opportunistic infections in HIV-infected Zambian children (CHAP): a double-blind randomised placebo-controlled trial. Lancet. 2004 Nov 20-26;364 (9448):1865-71.

52. Grimwade K, Sturm AW, Nunn AJ, Mbatha D, Zungu D, Gilks CF. Effectiveness of cotrimoxazole prophylaxis on mortality in adults with tuberculosis in rural South Africa. AIDS (London, England). 2005 Jan 28;19(2):163-8.

53. Mermin J, Lule J, Ekwaru JP, Malamba S, Downing R, Ransom R, et al. Effect of co-trimoxazole prophylaxis on morbidity, mortality, CD4-cell count, and viral load in HIV infection in rural Uganda. Lancet. 2004 Oct 16-22;364(9443):1428-34.

54. Wiktor SZ, Sassan-Morokro M, Grant AD, Abouya L, Karon JM, Maurice C, et al. Efficacy of trimethoprim-sulphamethoxazole prophylaxis to decrease morbidity and mortality in HIV-1-infected patients with tuberculosis in Abidjan, Cote d'Ivoire: a randomised controlled trial. Lancet. 1999 May 1;353(9163):1469-75.

55. Nunn AJ, Mwaba P, Chintu C, Mwinga A, Darbyshire JH, Zumla A. Role of co-trimoxazole prophylaxis in reducing mortality in HIV infected adults being treated for tuberculosis: randomised clinical trial. BMJ Clinical research ed. 2008;337:a257.

56. Guidelines on co-trimoxazole prophylaxis for HIV-related infections among children, adolescents and adults. WHO; 7 August 2006

57. A Strategic Framework for Malaria Prevention and Control during Pregnancy in the African region. WHO 2004; AFR/MAL/04/01

58. Institute of Medicine (2008). Assessment of the role of Intermittent Preventive Treatment for Malaria in Infants: Letter report. http://www.nap.edu/catalog/12180.html

59. DART Trial Team. Fixed Duration Interruptions are Inferior to Continuous Treatment in African Adults Starting Therapy with CD4<200 Cells/μL. AIDS 2008;22(2):237-47.

60. Kamya, Moses R; Gasasira, Anne F; Achan, Jane; Mebrahtu, Tsedal; Ruel, Theodore; Kekitiinwa, Adeodata; Charlebois, Edwin D; Rosenthal, Philip J; Havlir, Diane; Dorsey, Grant. Effects of trimethoprim-sulfamethoxazole and insecticide-treated bednets on malaria among HIV-infected Ugandan children. AIDS 2007, 21(15), 2007; 2059-2066

61. Moyle, Graeme J MD, MBBS; DeJesus, Edwin MD†; Cahn, Pedro MD; Castillo, Steve A MSc; Zhao, Henry PhD; Gordon, David N MB, ChB; Craig, Charles PhD; Scott, Trevor R PhD; for the Ziagen

Once-Daily in Antiretroviral Combination Therapy (CNA30021) Study Team. Abacavir Once or Twice Daily Combined With Once-Daily Lamivudine and Efavirenz for the Treatment of Antiretroviral-Naive HIV-Infected Adults: Results of the Ziagen Once Daily in Antiretroviral Combination Study. AIDS 2005; 38:4

62. LaMarca, Anthony MD; Clumeck, Nathan MD, PhD; Plettenberg, Andreas MD, PhD; Domingo, Pere MD, PhD; Fu, Kaisong MD, PhD; Craig, Charles PhD; Zhao, Henry PhD∥; Watson, Maria PhD; Gordon, David MB, ChB; Scott, Trevor PhD; on behalf of the CAL30001 Study Team. Efficacy and Safety of a Once-Daily Fixed-Dose Combination of Abacavir/Lamivudine Compared With Abacavir Twice Daily and Lamivudine Once Daily as Separate Entities in Antiretroviral-Experienced HIV-1-Infected Patients (CAL30001 Study) JAIDS 2006;41:598-606

63. Sosa, Nestor MD; Hill-Zabala, Christina PharmD; DeJesus, Edwin MD; Herrera, Gisella MD; Florance, Allison MS; Watson, Maria PhD; Vavro, Cindy; Shaefer, Mark PharmD. Abacavir and Lamivudine Fixed-Dose Combination Tablet Once Daily Compared With Abacavir and Lamivudine Twice Daily in HIV-Infected Patients Over 48 Weeks (ESS30008, SEAL). JAIDS 2005;40:422-7

64. D Maitland, A Jackson, J Osorio, S Mandalia, BG Gazzard, GJ Moyle and for the Epivir-Ziagen (EZ) Switch Study Team. Switching from twice-daily abacavir and lamivudine to the once-daily fixed-dose combination tablet of abacavir and lamivudine improves patient adherence and satisfaction with therapy. HIV Medicine 2008;9:667-72

65. Evelyne Jacqz-Aigrain, L Farrelly, A Compagnucci, L Harrison, W Zhao, D Hamadache, S Welch, U Wintergerst, G Firtion, D Burger, and Penta 15 Trial Steering Committee. Pharmacokinetic Study of Once- vs Twice-daily Abacavir and Lamivudine as Part of Combination ART in Children with HIV-1 Infection aged 3 to 36 months: Pediatric European Network for Treatment of AIDS 15 and ANRS 133. 16th Conference on Retroviruses and Opportunistic Infections, February 2009, Montréal, Canada.

Appendix 1.0 Details of Antiretroviral Therapy

Detailed information on all drugs will be provided on regularly updated disk from EMEA website and in individual drug brochures

Drugs will be prescribed according to weight bands used in WHO tables. Scored tablets as well as liquids will be provided for abacavir and 3TC.

**Drug (class) (recommended dosing)**	**Common toxicities**	**More severe toxicities**
	**Nucleoside Reverse Transcriptase Inhibitors (NRTIs)**	
**Abacavir (NRTI)**<br><br>Total daily dose = 16mg/kg	Nausea, vomiting, fever, headache, diarrhoea, rash, anorexia	Approximately 5% of children develop a potentially fatal hypersensitivity reaction. This percentage is likely to be lower in African children. Symptoms include fever, fatigue, malaise, nausea, vomiting, diarrhoea and abdominal pain or respiratory symptoms e.g. shortness of breath. Physical findings include lymphadenopathy, ulceration of mucous membranes and maculopapular or urticarial skin rash. Hypersensitivity can occur without a rash. Lab abnormalities include elevated liver function tests, increased creatine phosphokinase & lymphopenia. Most common in first 6 weeks of therapy.<br>Unusual (severe): Pancreatitis, increased liver enzymes, elevated blood glucose, elevated triglycerides and fatigue.
**3TC (NRTI)**<br><br>Total daily dose = 8mg/kg	Headache, fatigue, nausea, diarrhoea, skin rash and abdominal pain.	Pancreatitis (mainly seen in children with advanced HIV infection receiving many other medications), peripheral neuropathy, decreased neutrophil count, increased liver enzymes.<br>Cases of lactic acidosis and severe hepatomegaly with steatosis have occurred. Some of these have been fatal.
**Zidovudine (NRTI)**<br><br>Total daily dose = 360 – 480 mg/m^2	Haematologic toxicity including anaemia and granulocytopenia. Headache, nausea.	Myopathy, myositis and liver toxicity<br>Unusual (severe): Cases of lactic acidosis and severe hepatomegaly with steatosis have occurred. Some of these have been fatal.

Drug (class) (recommended dosing)	Common toxicities	More severe toxicities
**Didanosine (NRTI)**<br>Total daily dose = 240 mg/m²	Diarrhoea, abdominal pain, nausea, vomiting.	Peripheral neuropathy (dose related), electrolyte disturbances and hyperuricaemia.<br>Cases of lactic acidosis and severe hepatomegaly with steatosis have occurred. Some of these have been fatal. Rarely - pancreatitis (dose related, less common in children than adults). Increased liver enzymes and retinal depigmentation.
**d4T (NRTI)**<br>Total daily dose = 2 mg/kg	Headache, gastrointestinal disturbances and skin rashes.	Peripheral neuropathy and pancreatitis. Cases of lactic acidosis and severe hepatomegaly with steatosis have occurred. Some of these have been fatal. Fat Redistribution. Increased liver enzymes.
**Tenofovir (NRTI)**<br>Total daily dose = 300mg (adult dose – will be used in adolescents only)	Nausea, diarrhoea, vomiting in >10% of adults treated. Flatulence in 1 – 10%. Approximately 1% discontinued due to gastrointestinal side effects. Hypophosphataemia in >10%.	Cases of lactic acidosis and severe hepatomegaly with steatosis have been reported with use of the nucleoside analogues. Some of these have been fatal.
**Non-Nucleoside Reverse Transcriptase Inhibitors (NNRTIs)**		
**Nevirapine (NNRTI)**<br><br>**Weight Total daily dose (mg)**<br>3 -<6 kg 100<br>6-<10kg 150<br>10-<15kg 200<br>15-<20kg 250<br>20-<25kg 300<br>≥ 25kg 400	Skin rash in 10-20%. Can treat through mild/moderate without constitutional symptoms but some are severe requiring hospitalisation and life-threatening including Stevens-Johnson syndrome, toxic epidermal necrolysis), fever, nausea, headache and abnormal liver function tests.	Hepatitis which may rarely lead to severe and life-threatening and in some cases fatal liver damage. Very rarely - liver failure and granulocytopenia.<br>Hypersensitivity reactions (including, but not limited to severe rash or rash with fever, blisters, oral lesions, conjunctivitis, facial oedema, muscle or joint aches, general malaise +/ or significant hepatic abnormalities.
**Efavirenz (NNRTI)**<br>≥3 years & ≥ 13 kg<br><br>**Weight Total daily dose (mg)**<br>13 - ≤ 15 kg 200<br>>15 - ≤ 20kg 250<br>>20 - ≤ 25kg 300<br>>25 - ≤ 32.5kg 350<br>>32.5 -≤ 40 kg 400<br>≥ 40kg 600	Skin rash, CNS system (somnolence, insomnia, abnormal dreams, 'Spacey kids', confusion, abnormal thinking, impaired concentration, amnesia, agitation, depersonalisation, hallucinations, euphoria) mainly reported in adults: increased transaminase levels, teratogenic in primates (use in pregnancy should be avoided).	
**Protease Inhibitors (PIs)**		
**Ritonavir (PI)**<br>Total daily dose = 700mg/m²	Nausea, vomiting, diarrhoea, headache, abdominal pain and anorexia.	Circumoral parenthesis and increases in liver enzymes.<br>Fat redistribution and lipid abnormalities.

Drug (class) (recommended dosing)	Common toxicities	More severe toxicities
**Saquinavir (PI)**<br>Total daily dose = 100mg/kg	Diarrhoea, abdominal discomfort, headache, nausea, parenthesis and skin rash.	Exacerbation of chronic liver disease. Fat redistribution and lipid abnormalities.
**Kaletra (PI)**<br>Total daily dose = 460- 600mg/m^2	Diarrhoea, headache, asthenia, nausea and vomiting and rash (2%) in patients taking lopinavir with other antiretroviral drugs.	Fat redistribution and lipid abnormalities.

Appendix 2.0 Toxicity Gradings and Management

2.1 Table of Toxicity Gradings

Division of AIDS table for grading the severity of adverse events (published December 2004, clarification August 2009) apart from neutrophil gradings, which are based on WHO guidelines [9] and NIH Paediatric toxicity tables [44].

ULN = Upper Limit of Normal ; LLN = Lower Limit of Normal

CLINICAL				
**PARAMETER**	**GRADE 1<br>MILD**	**GRADE 2<br>MODERATE**	**GRADE 3<br>SEVERE**	**GRADE 4<br>POTENTIALLY LIFE-THREATENING**
**ESTIMATING SEVERITY GRADE**				
Clinical adverse event NOT identified elsewhere in this DAIDS AE grading table	Symptoms causing no or minimal interference with usual social & functional activities	Symptoms causing greater than minimal interference with usual social & functional activities	Symptoms causing inability to perform usual social & functional activities	Symptoms causing inability to perform basic self-care functions OR Medical or operative intervention indicated to prevent permanent impairment, persistent disability, or death
**SYSTEMIC**				
Acute systemic allergic reaction	Localized urticaria (wheals) with no medical intervention indicated	Localized urticaria with medical intervention indicated OR Mild angioedema with no medical intervention indicated	Generalized urticaria OR Angioedema with medical intervention indicated OR Symptomatic mild bronchospasm	Acute anaphylaxis OR Life-threatening bronchospasm OR laryngeal edema
Chills	Symptoms causing no or minimal interference with usual social & functional activities	Symptoms causing greater than minimal interference with usual social & functional activities	Symptoms causing inability to perform usual social & functional activities	NA
Fatigue<br>Malaise	Symptoms causing no or minimal interference with usual social & functional activities	Symptoms causing greater than minimal interference with usual social & functional activities	Symptoms causing inability to perform usual social & functional activities	Incapacitating fatigue/ malaise symptoms causing inability to perform basic self-care functions
Fever (nonaxillary)	37.7 – 38.6°C	38.7 – 39.3°C	39.4 – 40.5°C	> 40.5°C
Pain (indicate body site)<br>DO NOT use for pain due to injection (See Injection Site Reactions: Injection site pain)<br>See also Headache, Arthralgia, and Myalgia	Pain causing no or minimal interference with usual social & functional activities	Pain causing greater than minimal interference with usual social & functional activities	Pain causing inability to perform usual social & functional activities	Disabling pain causing inability to perform basic self-care functions OR Hospitalization (other than emergency room visit) indicated
Unintentional weight loss	NA	5 – 9% loss in body weight from baseline	10 – 19% loss in body weight from baseline	≥ 20% loss in body weight from baseline OR Aggressive intervention indicated [e.g., tube feeding or total parenteral nutrition (TPN)]

CLINICAL				
**PARAMETER**	**GRADE 1 MILD**	**GRADE 2 MODERATE**	**GRADE 3 SEVERE**	**GRADE 4 POTENTIALLY LIFE-THREATENING**
**INFECTION**				
Infection (any other than HIV infection)	Localized, no systemic antimicrobial treatment indicated AND Symptoms causing no or minimal interference with usual social & functional activities	Systemic antimicrobial treatment indicated OR Symptoms causing greater than minimal interference with usual social & functional activities	Systemic antimicrobial treatment indicated AND Symptoms causing inability to perform usual social & functional activities OR Operative intervention (other than simple incision and drainage) indicated	Life-threatening consequences (e.g., septic shock)
**INJECTION SITE REACTIONS**				
Injection site pain (pain without touching)<br>Or<br>Tenderness (pain when area is touched)	Pain/tenderness causing no or minimal limitation of use of limb	Pain/tenderness limiting use of limb OR Pain/tenderness causing greater than minimal interference with usual social & functional activities	Pain/tenderness causing inability to perform usual social & functional activities	Pain/tenderness causing inability to perform basic self-care function OR Hospitalization (other than emergency room visit) indicated for management of pain/tenderness
Injection site reaction (localized)				
**Adult > 15 years**	Erythema OR Induration of 5x5 cm – 9x9 cm (or 25 cm^2 – 81cm^2)	Erythema OR Induration OR Edema > 9 cm any diameter (or > 81 cm^2)	Ulceration OR Secondary infection OR Phlebitis OR Sterile abscess OR Drainage	Necrosis (involving dermis and deeper tissue)
**Paediatric ≤ 15 years**	Erythema OR Induration OR Edema present but ≤ 2.5 cm diameter	Erythema OR Induration OR Edema > 2.5 cm diameter but < 50% surface area of the extremity segment (e.g., upper arm/thigh)	Erythema OR Induration OR Edema involving ≥ 50% surface area of the extremity segment (e.g., upper arm/thigh) OR Ulceration OR Secondary infection OR Phlebitis OR Sterile abscess OR Drainage	Necrosis (involving dermis and deeper tissue)
Pruritis associated with injection<br>See also Skin: Pruritis (itching - no skin lesions)	Itching localized to injection site AND Relieved spontaneously or with < 48 hours treatment	Itching beyond the injection site but not generalized OR Itching localized to injection site requiring ≥ 48 hours treatment	Generalized itching causing inability to perform usual social & functional activities	NA
**SKIN – DERMATOLOGICAL**				
Alopecia	Thinning detectable by study participant (or by caregiver for young children and disabled adults)	Thinning or patchy hair loss detectable by health care provider	Complete hair loss	NA

CLINICAL				
**PARAMETER**	**GRADE 1 MILD**	**GRADE 2 MODERATE**	**GRADE 3 SEVERE**	**GRADE 4 POTENTIALLY LIFE-THREATENING**
Cutaneous reaction – rash	Localized macular rash	Diffuse macular, maculopapular, or morbilliform rash OR Target lesions	Diffuse macular, maculopapular, or morbilliform rash with vesicles or limited number of bullae OR Superficial ulcerations of mucous membrane limited to one site	Extensive or generalized bullous lesions OR Stevens-Johnson syndrome OR Ulceration of mucous membrane involving two or more distinct mucosal sites OR Toxic epidermal necrolysis (TEN)
Hyperpigmentation	Slight or localized	Marked or generalized	NA	NA
Hypopigmentation	Slight or localized	Marked or generalized	NA	NA
Pruritis (itching – no skin lesions)<br>(See also Injection Site Reactions: Pruritis associated with injection)	Itching causing no or minimal interference with usual social & functional activities	Itching causing greater than minimal interference with usual social & functional activities	Itching causing inability to perform usual social & functional activities	NA
**CARDIOVASCULAR**				
Cardiac arrhythmia (general)<br>(By ECG or physical exam)	Asymptomatic AND No intervention indicated	Asymptomatic AND Non-urgent medical intervention indicated	Symptomatic, non-life-threatening AND Non-urgent medical intervention indicated	Life-threatening arrhythmia OR Urgent intervention indicated
Cardiac-ischemia/infarction	NA	NA	Symptomatic ischemia (stable angina) OR Testing consistent with ischemia	Unstable angina OR Acute myocardial infarction
Hemorrhage (significant acute blood loss)	NA	Symptomatic AND No transfusion indicated	Symptomatic AND Transfusion of ≤ 2 units packed RBCs (for children ≤ 10 cc/kg) indicated	Life-threatening hypotension OR Transfusion of > 2 units packed RBCs (for children > 10 cc/kg) indicated
Hypertension				
**Adult > 17 years**<br>(with repeat testing at same visit)	> 140 – 159 mmHg systolic<br>OR<br>> 90 – 99 mmHg diastolic	160 – 179 mmHg systolic<br>OR<br>100 – 109 mmHg diastolic	≥ 180 mmHg systolic<br>OR<br>≥ 110 mmHg diastolic	Life-threatening consequences (e.g., malignant hypertension) OR Hospitalization indicated (other than emergency room visit)
**Paediatric ≤ 17 years**<br>(with repeat testing at same visit)	NA	91st – 94th percentile adjusted for age, height, and gender (systolic and/or diastolic)	≥ 95th percentile adjusted for age, height, and gender (systolic and/or diastolic)	Life-threatening consequences (e.g., malignant hypertension) OR Hospitalization indicated (other than emergency room visit)
Hypotension	NA	Symptomatic, corrected with oral fluid replacement	Symptomatic, IV fluids indicated	Shock requiring use of vasopressors or mechanical assistance to maintain blood pressure

CLINICAL				
**PARAMETER**	**GRADE 1 MILD**	**GRADE 2 MODERATE**	**GRADE 3 SEVERE**	**GRADE 4 POTENTIALLY LIFE-THREATENING**
Pericardial effusion	Asymptomatic, small effusion requiring no intervention	Asymptomatic, moderate or larger effusion requiring no intervention	Effusion with non-life threatening physiologic consequences OR Effusion with non-urgent intervention indicated	Life-threatening consequences (e.g., tamponade) OR Urgent intervention indicated
Prolonged PR interval				
**Adult > 16 years**	PR interval 0.21 – 0.25 sec	PR interval > 0.25 sec	Type II 2nd degree AV block OR Ventricular pause > 3.0 sec	Complete AV block
**Paediatric ≤ 16 years**	1st degree AV block (PR > normal for age and rate)	Type I 2nd degree AV block	Type II 2nd degree AV block	Complete AV block
Prolonged QTc				
**Adult > 16 years**	Asymptomatic, QTc interval 0.45 – 0.47 sec OR Increase interval < 0.03 sec above baseline	Asymptomatic, QTc interval 0.48 – 0.49 sec OR Increase in interval 0.03 – 0.05 sec above baseline	Asymptomatic, QTc interval ≥ 0.50 sec OR Increase in interval ≥ 0.06 sec above baseline	Life-threatening consequences, e.g. Torsade de pointes or other associated serious ventricular dysrhythmia
**Paediatric ≤ 16 years**	Asymptomatic, QTc interval 0.450 – 0.464 sec	Asymptomatic, QTc interval 0.465 – 0.479 sec	Asymptomatic, QTc interval ≥ 0.480 sec	Life-threatening consequences, e.g. Torsade de pointes or other associated serious ventricular dysrhythmia
Thrombosis/embolism	NA	Deep vein thrombosis AND No intervention indicated (e.g., anticoagulation, lysis filter, invasive procedure)	Deep vein thrombosis AND Intervention indicated (e.g., anticoagulation, lysis filter, invasive procedure)	Embolic event (e.g., pulmonary embolism, life-threatening thrombus)
Vasovagal episode (associated with a procedure of any kind)	Present without loss of consciousness	Present with transient loss of consciousness	NA	NA
Ventricular dysfunction (congestive heart failure)	NA	Asymptomatic diagnostic finding AND intervention indicated	New onset with symptoms OR Worsening symptomatic congestive heart failure	Life-threatening congestive heart failure
**GASTROINTESTINAL**				
Anorexia	Loss of appetite without decreased oral intake	Loss of appetite associated with decreased oral intake without significant weight loss	Loss of appetite associated with significant weight loss	Life-threatening consequences OR Aggressive intervention indicated [e.g., tube feeding or total parenteral nutrition (TPN)]
**Comment:** Please note that, while the grading scale provided for Unintentional Weight Loss may be used as a guideline when grading anorexia, this is not a requirement and should not be used as a substitute for clinical judgment.				
Ascites	Asymptomatic	Symptomatic AND Intervention indicated (e.g., diuretics or therapeutic paracentesis)	Symptomatic despite intervention	Life-threatening consequences

CLINICAL				
**PARAMETER**	**GRADE 1 MILD**	**GRADE 2 MODERATE**	**GRADE 3 SEVERE**	**GRADE 4 POTENTIALLY LIFE-THREATENING**
Cholecystitis	NA	Symptomatic AND Medical intervention indicated	Radiologic, endoscopic, or operative intervention indicated	Life-threatening consequences (e.g., sepsis or perforation)
Constipation	NA	Persistent constipation requiring regular use of dietary modifications, laxatives, or enemas	Obstipation with manual evacuation indicated	Life-threatening consequences (e.g., obstruction)
Diarrhea				
**Adult and Paediatric ≥ 1 year**	Transient or intermittent episodes of unformed stools OR Increase of ≤ 3 stools over baseline per 24-hour period	Persistent episodes of unformed to watery stools OR Increase of 4 – 6 stools over baseline per 24-hour period	Bloody diarrhea OR Increase of ≥ 7 stools per 24-hour period OR IV fluid replacement indicated	Life-threatening consequences (e.g., hypotensive shock)
**Paediatric < 1 year**	Liquid stools (more unformed than usual) but usual number of stools	Liquid stools with increased number of stools OR Mild dehydration	Liquid stools with moderate dehydration	Liquid stools resulting in severe dehydration with aggressive rehydration indicated OR Hypotensive shock
Dysphagia-Odynophagia	Symptomatic but able to eat usual diet	Symptoms causing altered dietary intake without medical intervention indicated	Symptoms causing severely altered dietary intake with medical intervention indicated	Life-threatening reduction in oral intake
Mucositis/stomatitis (clinical exam)<br>Indicate site (e.g., larynx, oral)<br>See Genitourinary for Vulvovaginitis<br>See also Dysphagia-Odynophagia and Proctitis	Erythema of the mucosa	Patchy pseudomembranes or ulcerations	Confluent pseudomembranes or ulcerations OR Mucosal bleeding with minor trauma	Tissue necrosis OR Diffuse spontaneous mucosal bleeding OR Life-threatening consequences (e.g., aspiration, choking)
Nausea	Transient (< 24 hours) or intermittent nausea with no or minimal interference with oral intake	Persistent nausea resulting in decreased oral intake for 24 – 48 hours	Persistent nausea resulting in minimal oral intake for > 48 hours OR Aggressive rehydration indicated (e.g., IV fluids)	Life-threatening consequences (e.g., hypotensive shock)
Pancreatitis	NA	Symptomatic AND Hospitalization not indicated (other than emergency room visit)	Symptomatic AND Hospitalization indicated (other than emergency room visit)	Life-threatening consequences (e.g., circulatory failure, hemorrhage, sepsis)
Proctitis (functional-symptomatic)<br>Also see Mucositis/stomatitis for clinical exam	Rectal discomfort AND No intervention indicated	Symptoms causing greater than minimal interference with usual social & functional activities OR Medical intervention indicated	Symptoms causing inability to perform usual social & functional activities OR Operative intervention indicated	Life-threatening consequences (e.g., perforation)

CLINICAL				
**PARAMETER**	**GRADE 1 MILD**	**GRADE 2 MODERATE**	**GRADE 3 SEVERE**	**GRADE 4 POTENTIALLY LIFE-THREATENING**
Vomiting	Transient or intermittent vomiting with no or minimal interference with oral intake	Frequent episodes of vomiting with no or mild dehydration	Persistent vomiting resulting in orthostatic hypotension OR Aggressive rehydration indicated (e.g., IV fluids)	Life-threatening consequences (e.g., hypotensive shock)
**NEUROLOGIC**				
Alteration in personality-behavior or in mood (e.g., agitation, anxiety, depression, mania, psychosis)	Alteration causing no or minimal interference with usual social & functional activities	Alteration causing greater than minimal interference with usual social & functional activities	Alteration causing inability to perform usual social & functional activities	Behavior potentially harmful to self or others (e.g., suicidal and homicidal ideation or attempt, acute psychosis) OR Causing inability to perform basic self-care functions
Altered Mental Status<br>For Dementia, see Cognitive and behavioral/attentional disturbance (including dementia and attention deficit disorder)	Changes causing no or minimal interference with usual social & functional activities	Mild lethargy or somnolence causing greater than minimal interference with usual social & functional activities	Confusion, memory impairment, lethargy, or somnolence causing inability to perform usual social & functional activities	Delirium OR obtundation, OR coma
Ataxia	Asymptomatic ataxia detectable on exam OR Minimal ataxia causing no or minimal interference with usual social & functional activities	Symptomatic ataxia causing greater than minimal interference with usual social & functional activities	Symptomatic ataxia causing inability to perform usual social & functional activities	Disabling ataxia causing inability to perform basic self-care functions
Cognitive and behavioral/attentional disturbance (including dementia and attention deficit disorder)	Disability causing no or minimal interference with usual social & functional activities OR Specialized resources not indicated	Disability causing greater than minimal interference with usual social & functional activities OR Specialized resources on part-time basis indicated	Disability causing inability to perform usual social & functional activities OR Specialized resources on a full-time basis indicated	Disability causing inability to perform basic self-care functions OR Institutionalization indicated
CNS ischemia (acute)	NA	NA	Transient ischemic attack	Cerebral vascular accident (CVA, stroke) with neurological deficit
Developmental delay – **Paediatric ≤ 16 years**	Mild developmental delay, either motor or cognitive, as determined by comparison with a developmental screening tool appropriate for the setting	Moderate developmental delay, either motor or cognitive, as determined by comparison with a developmental screening tool appropriate for the setting	Severe developmental delay, either motor or cognitive, as determined by comparison with a developmental screening tool appropriate for the setting	Developmental regression, either motor or cognitive, as determined by comparison with a developmental screening tool appropriate for the setting
Headache	Symptoms causing no or minimal interference with usual social & functional activities	Symptoms causing greater than minimal interference with usual social & functional activities	Symptoms causing inability to perform usual social & functional activities	Symptoms causing inability to perform basic self-care functions OR Hospitalization indicated (other than emergency room visit) OR Headache with significant impairment of alertness or other neurologic function

CLINICAL				
**PARAMETER**	**GRADE 1 MILD**	**GRADE 2 MODERATE**	**GRADE 3 SEVERE**	**GRADE 4 POTENTIALLY LIFE-THREATENING**
Insomnia	NA	Difficulty sleeping causing greater than minimal interference with usual social & functional activities	Difficulty sleeping causing inability to perform usual social & functional activities	Disabling insomnia causing inability to perform basic self-care functions
Neuromuscular weakness (including myopathy & neuropathy)	Asymptomatic with decreased strength on exam OR Minimal muscle weakness causing no or minimal interference with usual social & functional activities	Muscle weakness causing greater than minimal interference with usual social & functional activities	Muscle weakness causing inability to perform usual social & functional activities	Disabling muscle weakness causing inability to perform basic self-care functions OR Respiratory muscle weakness impairing ventilation
Neurosensory alteration (including paresthesia and painful neuropathy)	Asymptomatic with sensory alteration on exam or minimal paresthesia causing no or minimal interference with usual social & functional activities	Sensory alteration or paresthesia causing greater than minimal interference with usual social & functional activities	Sensory alteration or paresthesia causing inability to perform usual social & functional activities	Disabling sensory alteration or paresthesia causing inability to perform basic self-care functions
Seizure: (new onset) – **Adult ≥ 18 years**<br>See also Seizure: (known pre-existing seizure disorder)	NA	1 seizure	2 – 4 seizures	Seizures of any kind which are prolonged, repetitive (e.g., status epilepticus), or difficult to control (e.g., refractory epilepsy)
Seizure: (known pre-existing seizure disorder) **– Adult ≥ 18 years**<br>For worsening of existing epilepsy the grades should be based on an increase from previous level of control to any of these levels.	NA	Increased frequency of pre-existing seizures (non-repetitive) without change in seizure character OR Infrequent break-through seizures while on stable medication in a previously controlled seizure disorder	Change in seizure character from baseline either in duration or quality (e.g., severity or focality)	Seizures of any kind which are prolonged, repetitive (e.g., status epilepticus), or difficult to control (e.g., refractory epilepsy)
Seizure **– Paediatric < 18 years**	Seizure, generalized onset with or without secondary generalization, lasting < 5 minutes with < 24 hours post ictal state	Seizure, generalized onset with or without secondary generalization, lasting 5 – 20 minutes with < 24 hours post ictal state	Seizure, generalized onset with or without secondary generalization, lasting > 20 minutes	Seizure, generalized onset with or without secondary generalization, requiring intubation and sedation
Syncope (not associated with a procedure)	NA	Present	NA	NA
Vertigo	Vertigo causing no or minimal interference with usual social & functional activities	Vertigo causing greater than minimal interference with usual social & functional activities	Vertigo causing inability to perform usual social & functional activities	Disabling vertigo causing inability to perform basic self-care functions

CLINICAL				
**PARAMETER**	**GRADE 1 MILD**	**GRADE 2 MODERATE**	**GRADE 3 SEVERE**	**GRADE 4 POTENTIALLY LIFE-THREATENING**
**RESPIRATORY**				
Bronchospasm (acute)	FEV1 or peak flow reduced to 70 – 80%	FEV1 or peak flow 50 – 69%	FEV1 or peak flow 25 – 49%	Cyanosis OR FEV1 or peak flow < 25% OR Intubation
Dyspnea or respiratory distress				
**Adult ≥ 14 years**	Dyspnea on exertion with no or minimal interference with usual social & functional activities	Dyspnea on exertion causing greater than minimal interference with usual social & functional activities	Dyspnea at rest causing inability to perform usual social & functional activities	Respiratory failure with ventilatory support indicated
**Paediatric < 14 years**	Wheezing OR minimal increase in respiratory rate for age	Nasal flaring OR Intercostal retractions OR Pulse oximetry 90 – 95%	Dyspnea at rest causing inability to perform usual social & functional activities OR Pulse oximetry < 90%	Respiratory failure with ventilatory support indicated
**MUSCULOSKELETAL**				
Arthralgia<br>See also Arthritis	Joint pain causing no or minimal interference with usual social & functional activities	Joint pain causing greater than minimal interference with usual social & functional activities	Joint pain causing inability to perform usual social & functional activities	Disabling joint pain causing inability to perform basic self-care functions
Arthritis<br>See also Arthralgia	Stiffness or joint swelling causing no or minimal interference with usual social & functional activities	Stiffness or joint swelling causing greater than minimal interference with usual social & functional activities	Stiffness or joint swelling causing inability to perform usual social & functional activities	Disabling joint stiffness or swelling causing inability to perform basic self-care functions
Bone Mineral Loss				
**Adult ≥ 21 years**	BMD t-score -2.5 to -1.0	BMD t-score < -2.5	Pathological fracture (including loss of vertebral height)	Pathologic fracture causing life-threatening consequences
**Paediatric < 21 years**	BMD z-score -2.5 to -1.0	BMD z-score < -2.5	Pathological fracture (including loss of vertebral height)	Pathologic fracture causing life-threatening consequences
Myalgia (non-injection site)	Muscle pain causing no or minimal interference with usual social & functional activities	Muscle pain causing greater than minimal interference with usual social & functional activities	Muscle pain causing inability to perform usual social & functional activities	Disabling muscle pain causing inability to perform basic self-care functions
Osteonecrosis	NA	Asymptomatic with radiographic findings AND No operative intervention indicated	Symptomatic bone pain with radiographic findings OR Operative intervention indicated	Disabling bone pain with radiographic findings causing inability to perform basic self-care functions

CLINICAL				
**PARAMETER**	**GRADE 1 MILD**	**GRADE 2 MODERATE**	**GRADE 3 SEVERE**	**GRADE 4 POTENTIALLY LIFE-THREATENING**
**GENITOURINARY**				
Cervicitis (symptoms) (For use in studies evaluating topical study agents) For other cervicitis see Infection: Infection (any other than HIV infection)	Symptoms causing no or minimal interference with usual social & functional activities	Symptoms causing greater than minimal interference with usual social & functional activities	Symptoms causing inability to perform usual social & functional activities	Symptoms causing inability to perform basic self-care functions
Cervicitis (clinical exam) (For use in studies evaluating topical study agents) For other cervicitis see Infection: Infection (any other than HIV infection)	Minimal cervical abnormalities on examination (erythema, mucopurulent discharge, or friability) OR Epithelial disruption < 25% of total surface	Moderate cervical abnormalities on examination (erythema, mucopurulent discharge, or friability) OR Epithelial disruption of 25 – 49% total surface	Severe cervical abnormalities on examination (erythema, mucopurulent discharge, or friability) OR Epithelial disruption 50 – 75% total surface	Epithelial disruption > 75% total surface
Inter-menstrual bleeding (IMB)	Spotting observed by participant OR Minimal blood observed during clinical or colposcopic examination	Inter-menstrual bleeding not greater in duration or amount than usual menstrual cycle	Inter-menstrual bleeding greater in duration or amount than usual menstrual cycle	Hemorrhage with life-threatening hypotension OR Operative intervention indicated
Urinary tract obstruction (e.g., stone)	NA	Signs or symptoms of urinary tract obstruction without hydronephrosis or renal dysfunction	Signs or symptoms of urinary tract obstruction with hydronephrosis or renal dysfunction	Obstruction causing life-threatening consequences
Vulvovaginitis (symptoms) (Use in studies evaluating topical study agents) For other vulvovaginitis see Infection: Infection (any other than HIV infection)	Symptoms causing no or minimal interference with usual social & functional activities	Symptoms causing greater than minimal interference with usual social & functional activities	Symptoms causing inability to perform usual social & functional activities	Symptoms causing inability to perform basic self-care functions
Vulvovaginitis (clinical exam) (Use in studies evaluating topical study agents) For other vulvovaginitis see Infection: Infection (any other than HIV infection)	Minimal vaginal abnormalities on examination OR Epithelial disruption < 25% of total surface	Moderate vaginal abnormalities on examination OR Epithelial disruption of 25 - 49% total surface	Severe vaginal abnormalities on examination OR Epithelial disruption 50 - 75% total surface	Vaginal perforation OR Epithelial disruption > 75% total surface

CLINICAL				
**PARAMETER**	**GRADE 1 MILD**	**GRADE 2 MODERATE**	**GRADE 3 SEVERE**	**GRADE 4 POTENTIALLY LIFE-THREATENING**
**OCULAR/VISUAL**				
Uveitis	Asymptomatic but detectable on exam	Symptomatic anterior uveitis OR Medical intervention indicated	Posterior or pan-uveitis OR Operative intervention indicated	Disabling visual loss in affected eye(s)
Visual changes (from baseline)	Visual changes causing no or minimal interference with usual social & functional activities	Visual changes causing greater than minimal interference with usual social & functional activities	Visual changes causing inability to perform usual social & functional activities	Disabling visual loss in affected eye(s)
**ENDOCRINE/METABOLIC**				
Abnormal fat accumulation (e.g., back of neck, breasts, abdomen)	Detectable by study participant (or by caregiver for young children and disabled adults)	Detectable on physical exam by health care provider	Disfiguring OR Obvious changes on casual visual inspection	NA
Diabetes mellitus	NA	New onset without need to initiate medication OR Modification of current medications to regain glucose control	New onset with initiation of medication indicated OR Diabetes uncontrolled despite treatment modification	Life-threatening consequences (e.g., ketoacidosis, hyperosmolar non-ketotic coma)
Gynecomastia	Detectable by study participant or caregiver (for young children and disabled adults)	Detectable on physical exam by health care provider	Disfiguring OR Obvious on casual visual inspection	NA
Hyperthyroidism	Asymptomatic	Symptomatic causing greater than minimal interference with usual social & functional activities OR Thyroid suppression therapy indicated	Symptoms causing inability to perform usual social & functional activities OR Uncontrolled despite treatment modification	Life-threatening consequences (e.g., thyroid storm)
Hypothyroidism	Asymptomatic	Symptomatic causing greater than minimal interference with usual social & functional activities OR Thyroid replacement therapy indicated	Symptoms causing inability to perform usual social & functional activities OR Uncontrolled despite treatment modification	Life-threatening consequences (e.g., myxedema coma)
Lipoatrophy (e.g., fat loss from the face, extremities, buttocks)	Detectable by study participant (or by caregiver for young children and disabled adults)	Detectable on physical exam by health care provider	Disfiguring OR Obvious on casual visual inspection	NA

LABORATORY				
**PARAMETER**	**GRADE 1 MILD**	**GRADE 2 MODERATE**	**GRADE 3 SEVERE**	**GRADE 4 POTENTIALLY LIFE-THREATENING**
**HAEMATOLOGY**	***Standard International Units are listed in italics***			

CLINICAL				
**PARAMETER**	**GRADE 1<br>MILD**	**GRADE 2<br>MODERATE**	**GRADE 3<br>SEVERE**	**GRADE 4<br>POTENTIALLY<br>LIFE-THREATENING**
Absolute CD4+ count<br>**– Adult and Paediatric > 13 years**<br>(HIV NEGATIVE ONLY)	300 – 400/mm^3<br>*300 – 400/µL*	200 – 299/mm^3<br>*200 – 299/µL*	100 – 199/mm^3<br>*100 – 199/µL*	< 100/mm^3<br>*< 100/µL*
Absolute lymphocyte count<br>**– Adult and Paediatric > 13 years**<br>(HIV NEGATIVE ONLY)	600 – 650/mm^3<br>*0.600 x 10^9 – 0.650 x 10^9/L*	500 – 599/mm^3<br>*0.500 x 10^9 – 0.599 x 10^9/L*	350 – 499/mm^3<br>*0.350 x 10^9 – 0.499 x 10^9/L*	< 350/mm^3<br>*< 0.350 x 10^9/L*
**Comment:** Values in children ≤ 13 years are not given for the two parameters above because the absolute counts are variable				
Absolute neutrophil count (ANC) [9, 44]				
**Adult and Paediatric, > 7 days**	750 –< 1,000/mm^3<br>*0.75 x 10^9 – <1.0. x 10^9/L*	500 – 749/mm^3<br>*0.5 x 10^9 – 0.749 x 10^9/L*	250 – 499/mm^3<br>*0.25 x 10^9 – 0.499 x 10^9/L*	< 250/mm^3<br>*< 0.250 x 10^9/L*
**Infant*†, 2 – ≤ 7 days**	1,250 – 1,500/mm^3<br>*1.250 x 10^9 – 1.500 x 10^9/L*	1,000 – 1,249/mm^3<br>*1.000 x 10^9 – 1.249 x 10^9/L*	750 – 999/mm^3<br>*0.750 x 10^9 – 0.999 x 10^9/L*	< 750/mm^3<br>*< 0.750 x 10^9/L*
**Infant*†, ≤1 day**	4,000 – 5,000/mm^3<br>*4.000 x 10^9 – 5.000 x 10^9/L*	3,000 – 3,999/mm^3<br>*3.000 x 10^9 – 3.999 x10^9/L*	1,500 – 2,999/mm^3<br>*1.500 x 10^9 – 2.999 x 10^9/L*	< 1,500/mm^3<br>*< 1.500 x 10^9/L*
Fibrinogen, decreased	100 – 200 mg/dL<br>*1.00 – 2.00 g/L*<br>OR<br>0.75 – 0.99 x LLN	75 – 99 mg/dL<br>*0.75 – 0.99 g/L*<br>OR<br>0.50 – 0.74 x LLN	50 – 74 mg/dL<br>*0.50 – 0.74 g/L*<br>OR<br>0.25 – 0.49 x LLN	< 50 mg/dL<br>*< 0.50 g/L*<br>OR<br>< 0.25 x LLN<br>OR<br>Associated with gross bleeding
Haemoglobin (Hgb)				
**Comment:** The Hgb values in mmol/L have changed because the conversion factor used to convert g/dL to mmol/L has been changed from 0.155 to 0.6206 (the most commonly used conversion factor). For grading Hgb results obtained by an analytic method with a conversion factor other than 0.6206, the result must be converted to g/dL using the appropriate conversion factor for that lab.				
**Adult and Paediatric ≥ 57 days**<br>(HIV POSITIVE ONLY)	8.5 – 10.0 g/dL<br>*5.24 –6.23 mmol/L*	7.5 – 8.4 g/dL<br>*4.62-5.23 mmol/L*	6.50 – 7.4 g/dL<br>*4.03-4.61 mmol/L*	< 6.5 g/dL<br>*< 4.03 mmol/L*
**Adult and Paediatric ≥ 57 days**<br>(HIV NEGATIVE ONLY)	10.0 – 10.9 g/dL<br>*6.18 – 6.79 mmol/L*<br>OR<br>Any decrease<br>2.5 – 3.4 g/dL<br>*1.58 – 2.13 mmol/L*	9.0 – 9.9 g/dL<br>*5.55 – 6.17 mmol/L*<br>OR<br>Any decrease<br>3.5 – 4.4 g/dL<br>*2.14 – 2.78 mmol/L*	7.0 – 8.9 g/dL<br>*4.34 – 5.54 mmol/L*<br>OR<br>Any decrease<br>≥ 4.5 g/dL<br>*≥ 2.79 mmol/L*	< 7.0 g/dL<br>*< 4.34 mmol/L*
**Comment:** The decrease is a decrease from baseline				
**Infant*†, 36 – 56 days**<br>(HIV POSITIVE OR NEGATIVE)	8.5 – 9.4 g/dL<br>*5.24 – 5.86 mmol/L*	7.0 – 8.4 g/dL<br>*4.31 – 5.23 mmol/L*	6.0 – 6.9 g/dL<br>*3.72 – 4.30 mmol/L*	< 6.00 g/dL<br>*< 3.72 mmol/L*
**Infant*†, 22 – 35 days**<br>(HIV POSITIVE OR NEGATIVE)	9.5 – 10.5 g/dL<br>*5.86 – 6.54 mmol/L*	8.0 – 9.4 g/dL<br>*4.93 – 5.86 mmol/L*	7.0 – 7.9 g/dL<br>*4.34 – 4.92 mmol/L*	< 7.00 g/dL<br>*< 4.34 mmol/L*

CLINICAL				
**PARAMETER**	**GRADE 1**<br>**MILD**	**GRADE 2**<br>**MODERATE**	**GRADE 3**<br>**SEVERE**	**GRADE 4**<br>**POTENTIALLY LIFE-THREATENING**
**Infant[*,†], ≤21 days** (HIV POSITIVE OR NEGATIVE)	12.0 – 13.0 g/dL<br>*7.42 – 8.09 mmol/L*	10.0 – 11.9 g/dL<br>*6.18 – 7.41 mmol/L*	9.0 – 9.9 g/dL<br>*5.59 – 6.17 mmol/L*	< 9.0 g/dL<br>*< 5.59mmol/L*
**Comment:** Parametre changed from “Infant <21 days” to “Infants ≤ 21 days”				
International Normalized Ratio of prothrombin time (INR)	1.1 – 1.5 x ULN	1.6 – 2.0 x ULN	2.1 – 3.0 x ULN	> 3.0 x ULN
Methemoglobin	5.0 – 10.0%	10.1 – 15.0%	15.1 – 20.0%	> 20.0%
Prothrombin Time (PT)	1.1 – 1.25 x ULN	1.26 – 1.50 x ULN	1.51 – 3.00 x ULN	> 3.00 x ULN
Partial Thromboplastin Time (PTT)	1.1 – 1.66 x ULN	1.67 – 2.33 x ULN	2.34 – 3.00 x ULN	> 3.00 x ULN
Platelets, decreased	100,000 – 124,999/mm^3<br>*100.000 x 10^9 – 124.999 x 10^9/L*	50,000 – 99,999/mm^3<br>*50.000 x 10^9 – 99.999 x 10^9/L*	25,000 – 49,999/mm^3<br>*25.000 x 10^9 – 49.999 x 10^9/L*	< 25,000/mm^3<br>*< 25.000 x 10^9/L*
WBC, decreased	2,000 – 2,500/mm^3<br>*2.000 x 10^9 – 2.500 x 10^9/L*	1,500 – 1,999/mm^3<br>*1.500 x 10^9 – 1.999 x 10^9/L*	1,000 – 1,499/mm^3<br>*1.000 x 10^9 – 1.499 x 10^9/L*	< 1,000/mm^3<br>*< 1.000 x 10^9/L*
**CHEMISTRIES**	***Standard International Units are listed in italics***			
Acidosis	NA	pH < normal, but ≥ 7.3	pH < 7.3 without life-threatening consequences	pH < 7.3 with life-threatening consequences
Albumin, serum, low	3.0 g/dL – < LLN<br>*30 g/L – < LLN*	2.0 – 2.9 g/dL<br>*20 – 29 g/L*	< 2.0 g/dL<br>*< 20 g/L*	NA
Alkaline Phosphatase	1.25 – 2.5 x ULN[†]	2.6 – 5.0 x ULN[†]	5.1 – 10.0 x ULN[†]	> 10.0 x ULN[†]
Alkalosis	NA	pH > normal, but ≤ 7.5	pH > 7.5 without life-threatening consequences	pH > 7.5 with life-threatening consequences
ALT (SGPT)	1.25 – 2.5 x ULN	2.6 – 5.0 x ULN	5.1 – 10.0 x ULN	> 10.0 x ULN
AST (SGOT)	1.25 – 2.5 x ULN	2.6 – 5.0 x ULN	5.1 – 10.0 x ULN	> 10.0 x ULN
Bicarbonate, serum, low	16.0 mEq/L – < LLN<br>*16.0 mmol/L – < LLN*	11.0 – 15.9 mEq/L<br>*11.0 – 15.9 mmol/L*	8.0 – 10.9 mEq/L<br>*8.0 – 10.9 mmol/L*	< 8.0 mEq/L<br>*< 8.0 mmol/L*
**Comment :** Some laboratories will report this value as Bicarbonate (HCO_3) and others as Total Carbon Dioxide (CO_2). These are the same tests, values should be graded according to the ranges for Bicarbonate as listed above.				
Bilirubin (Total)				
**Adult and Paediatric > 14 days**	1.1 – 1.5 x ULN	1.6 – 2.5 x ULN	2.6 – 5.0 x ULN	> 5.0 x ULN
**Infant[*,†], ≤ 14 days** (non-hemolytic)	NA	20.0 – 25.0 mg/dL<br>*342 – 428 µmol/L*	25.1 – 30.0 mg/dL<br>*429 – 513 µmol/L*	> 30.0 mg/dL<br>*> 513.0 µmol/L*
**Infant[*,†], ≤ 14 days** (hemolytic)	NA	NA	20.0 – 25.0 mg/dL<br>*342 – 428 µmol/L*	> 25.0 mg/dL<br>*> 428 µmol/L*
Calcium, serum, high (corrected for albumin)				
**Adult and Paediatric ≥ 7 days**	10.6 – 11.5 mg/dL<br>*2.65 – 2.88 mmol/L*	11.6 – 12.5 mg/dL<br>*2.89 – 3.13 mmol/L*	12.6 – 13.5 mg/dL<br>*3.14 – 3.38 mmol/L*	> 13.5 mg/dL<br>*> 3.38 mmol/L*

CLINICAL				
**PARAMETER**	**GRADE 1 MILD**	**GRADE 2 MODERATE**	**GRADE 3 SEVERE**	**GRADE 4 POTENTIALLY LIFE-THREATENING**
**Infant*†, < 7 days**	11.5 – 12.4 mg/dL<br>*2.88 – 3.10 mmol/L*	12.5 – 12.9 mg/dL<br>*3.11 – 3.23 mmol/L*	13.0 – 13.5 mg/dL<br>*3.245 – 3.38 mmol/L*	> 13.5 mg/dL<br>*> 3.38 mmol/L*
Calcium, serum, low (corrected for albumin)				
**Adult and Paediatric ≥ 7 days**	7.8 – 8.4 mg/dL<br>*1.95 – 2.10 mmol/L*	7.0 – 7.7 mg/dL<br>*1.75 – 1.94 mmol/L*	6.1 – 6.9 mg/dL<br>*1.53 – 1.74 mmol/L*	< 6.1 mg/dL<br>*< 1.53 mmol/L*
**Infant*†, < 7 days**	6.5 – 7.5 mg/dL<br>*1.63 – 1.88 mmol/L*	6.0 – 6.4 mg/dL<br>*1.50 – 1.62 mmol/L*	5.50 – 5.90 mg/dL<br>*1.38 – 1.51 mmol/L*	< 5.50 mg/dL<br>*< 1.38 mmol/L*
**Comment:** Do not adjust Calcium, serum, low or Calcium, serum, high for albumin				
Cardiac troponin I (cTnI)	NA	NA	NA	Levels consistent with myocardial infarction or unstable angina as defined by the manufacturer
Cardiac troponin T (cTnT)	NA	NA	NA	≥ 0.20 ng/mL<br>OR<br>Levels consistent with myocardial infarction or unstable angina as defined by the manufacturer
Cholesterol (fasting)				
**Adult ≥ 18 years**	200 – 239 mg/dL<br>*5.18 – 6.19 mmol/L*	240 – 300 mg/dL<br>*6.20 – 7.77 mmol/L*	> 300 mg/dL<br>*> 7.77 mmol/L*	NA
**Paediatric < 18 years**	170 – 199 mg/dL<br>*4.40 – 5.15 mmol/L*	200 – 300 mg/dL<br>*5.16 – 7.77 mmol/L*	> 300 mg/dL<br>*> 7.77 mmol/L*	NA
Creatine Kinase	3.0 – 5.9 x ULN†	6.0 – 9.9 x ULN†	10.0 – 19.9 x ULN†	≥ 20.0 x ULN†
Creatinine	1.1 – 1.3 x ULN†	1.4 – 1.8 x ULN†	1.9 – 3.4 x ULN†	≥ 3.5 x ULN†
Glucose, serum, high				
Nonfasting	116 – 160 mg/dL<br>*6.44 – 8.88 mmol/L*	161 – 250 mg/dL<br>*8.89 – 13.88 mmol/L*	251 – 500 mg/dL<br>*13.89 – 27.75 mmol/L*	> 500 mg/dL<br>*> 27.75 mmol/L*
Fasting	110 – 125 mg/dL<br>*6.11 – 6.94 mmol/L*	126 – 250 mg/dL<br>*6.95 – 13.88 mmol/L*	251 – 500 mg/dL<br>*13.89 – 27.75 mmol/L*	> 500 mg/dL<br>*> 27.75 mmol/L*
Glucose, serum, low				
**Adult and Paediatric ≥ 1 month**	55 – 64 mg/dL<br>*3.05 – 3.55 mmol/L*	40 – 54 mg/dL<br>*2.22 – 3.06 mmol/L*	30 – 39 mg/dL<br>*1.67 – 2.23 mmol/L*	< 30 mg/dL<br>*< 1.67 mmol/L*
**Infant*†, < 1 month**	50 – 54 mg/dL<br>*2.78 – 3.00 mmol/L*	40 – 49 mg/dL<br>*2.22 – 2.77 mmol/L*	30 – 39 mg/dL<br>*1.67 – 2.21 mmol/L*	< 30 mg/dL<br>*< 1.67 mmol/L*
Lactate	ULN- < 2.0 x ULN without acidosis	≥ 2.0 x ULN without acidosis	Increased lactate with pH < 7.3 without life-threatening consequences	Increased lactate with pH < 7.3 with life-threatening consequences
**Comment:** Added ULN to grade 1 parameter				
LDL cholesterol (fasting)				
**Adult ≥ 18 years**	130 – 159 mg/dL<br>*3.37 – 4.12 mmol/L*	160 – 190 mg/dL<br>*4.13 – 4.90 mmol/L*	≥ 190 mg/dL<br>*≥ 4.91 mmol/L*	NA
**Paediatric > 2 - < 18 years**	110 – 129 mg/dL<br>*2.85 – 3.34 mmol/L*	130 – 189 mg/dL<br>*3.35 – 4.90 mmol/L*	≥ 190 mg/dL<br>*≥ 4.91 mmol/L*	NA

CLINICAL				
**PARAMETER**	**GRADE 1 MILD**	**GRADE 2 MODERATE**	**GRADE 3 SEVERE**	**GRADE 4 POTENTIALLY LIFE-THREATENING**
Lipase	1.1 – 1.5 x ULN	1.6 – 3.0 x ULN	3.1 – 5.0 x ULN	> 5.0 x ULN
Magnesium, serum, low	1.2 – 1.4 mEq/L<br>*0.60 – 0.70 mmol/L*	0.9 – 1.1 mEq/L<br>*0.45 – 0.59 mmol/L*	0.6 – 0.8 mEq/L<br>*0.30 – 0.44 mmol/L*	< 0.60 mEq/L<br>*< 0.30 mmol/L*
Pancreatic amylase	1.1 – 1.5 x ULN	1.6 – 2.0 x ULN	2.1 – 5.0 x ULN	> 5.0 x ULN
Phosphate, serum, low				
**Adult and Paediatric > 14 years**	2.5 mg/dL – < LLN<br>*0.81 mmol/L – < LLN*	2.0 – 2.4 mg/dL<br>*0.65 – 0.80 mmol/L*	1.0 – 1.9 mg/dL<br>*0.32 – 0.64 mmol/L*	< 1.00 mg/dL<br>*< 0.32 mmol/L*
**Paediatric 1 year – 14 years**	3.0 – 3.5 mg/dL<br>*0.97 – 1.13 mmol/L*	2.5 – 2.9 mg/dL<br>*0.81 – 0.96 mmol/L*	1.5 – 2.4 mg/dL<br>*0.48 – 0.80 mmol/L*	< 1.50 mg/dL<br>*< 0.48 mmol/L*
**Paediatric < 1 year**	3.5 – 4.5 mg/dL<br>*1.13 – 1.45 mmol/L*	2.5 – 3.4 mg/dL<br>*0.81 – 1.12 mmol/L*	1.5 – 2.4 mg/dL<br>*0.48 – 0.80 mmol/L*	< 1.50 mg/dL<br>*< 0.48 mmol/L*
Potassium, serum, high	5.6 – 6.0 mEq/L<br>*5.6 – 6.0 mmol/L*	6.1 – 6.5 mEq/L<br>*6.1 – 6.5 mmol/L*	6.6 – 7.0 mEq/L<br>*6.6 – 7.0 mmol/L*	> 7.0 mEq/L<br>*> 7.0 mmol/L*
Potassium, serum, low	3.0 – 3.4 mEq/L<br>*3.0 – 3.4 mmol/L*	2.5 – 2.9 mEq/L<br>*2.5 – 2.9 mmol/L*	2.0 – 2.4 mEq/L<br>*2.0 – 2.4 mmol/L*	< 2.0 mEq/L<br>*< 2.0 mmol/L*
Sodium, serum, high	146 – 150 mEq/L<br>*146 – 150 mmol/L*	151 – 154 mEq/L<br>*151 – 154 mmol/L*	155 – 159 mEq/L<br>*155 – 159 mmol/L*	≥ 160 mEq/L<br>*≥160 mmol/L*
Sodium, serum, low	130 – 135 mEq/L<br>*130 – 135 mmol/L*	125 – 129 mEq/L<br>*125 – 129 mmol/L*	121 – 124 mEq/L<br>*121 – 124 mmol/L*	≤ 120 mEq/L<br>*≤120 mmol/L*
Triglycerides (fasting)	NA	500 – 750 mg/dL<br>*5.65 – 8.48 mmol/L*	751 – 1,200 mg/dL<br>*8.49 – 13.56 mmol/L*	> 1,200 mg/dL<br>*> 13.56 mmol/L*
Uric acid	7.5 – 10.0 mg/dL<br>*0.45 – 0.59 mmol/L*	10.1 – 12.0 mg/dL<br>*0.60 – 0.71 mmol/L*	12.1 – 15.0 mg/dL<br>*0.72 – 0.89 mmol/L*	> 15.0 mg/dL<br>*> 0.89 mmol/L*
**URINALYSIS**	***Standard International Units are listed in italics***			
Hematuria (microscopic)	6 – 10 RBC/HPF	> 10 RBC/HPF	Gross, with or without clots OR with RBC casts	Transfusion indicated
Proteinuria, random collection	1 +	2 – 3 +	4 +	NA
Proteinuria, 24 hour collection				
**Adult and Paediatric ≥ 10 years**	200 – 999 mg/24 h<br>*0.200 – 0.999 g/d*	1,000 – 1,999 mg/24 h<br>*1.000 – 1.999 g/d*	2,000 – 3,500 mg/24 h<br>*2.000 – 3.500 g/d*	> 3,500 mg/24 h<br>*> 3.500 g/d*
**Paediatric > 3 mo - < 10 years**	201 – 499 mg/m^2/24 h<br>*0.201 – 0.499 g/d*	500 – 799 mg/m^2/24 h<br>*0.500 – 0.799 g/d*	800 – 1,000 mg/m^2/24 h<br>*0.800 – 1.000 g/d*	> 1,000 mg/ m^2/24 h<br>*> 1.000 g/d*

[*]Values are for term infants. Preterm infants should be assessed using local normal ranges. [†]Use age and sex appropriate values (e.g., bilirubin), including preterm infants.

2.2 Table of Clinical Signs, Symptoms, Monitoring and Management of Symptoms of Serious Adverse Effects of Antiretroviral Drugs that Require Drug Discontinuation (adapted from WHO guidelines Annex 11B)

Adverse Effect	Possible Offending Drug(s)	Clinical Signs / Symptoms	Management
Acute hepatitis	Nevirapine (NVP); more uncommon with zidovudine (ZDV), didanosine (ddI), stavudine (d4T) (<1%); and protease inhibitors (PI), most frequently with ritonavir (RTV)	Jaundice, liver enlargement, gastrointestinal symptoms, fatigue, anorexia; NVP-associated hepatitis may have hypersensitivity component (drug rash, systemic symptoms, eosinophilia)	Monitor serum transaminases, bilirubin. All ARV should be stopped until symptoms resolve. NVP may need to be permanently discontinued.
Acute pancreatitis	ddI, d4T	Nausea, vomiting, and abdominal pain	If possible, monitor serum pancreatic amylase, lipase. All ART should be stopped until symptoms resolve. Restart ART with change to different NRTI, preferably one without pancreatic toxicity (e.g., ZDV)
Lactic acidosis	All nucleoside analogue reverse transcriptase inhibitors (NRTIs)	Initial symptoms are variable: a clinical prodromal syndrome may include generalized fatigue and weakness, gastrointestinal symptoms (nausea, vomiting, diarrhea, abdominal pain, hepatomegaly, anorexia, and/or sudden unexplained weight loss), respiratory symptoms (tachypnea and dyspnea) or neurologic symptoms (including motor weakness).	Discontinue all ARV; symptoms may continue or worsen after discontinuation of ART. Supportive therapy. Regimens that can be considered for restarting ART include a PI combined with an NNRTI and tenofovir
Hyper-sensitivity reaction	Abacavir (ABC) Nevirapine (NVP)	ABC: Constellation of acute onset of symptoms including: fever, fatigue, myalgia, nausea/vomiting, diarrhea, abdominal pain, pharyngitis, cough, dyspnea (with or without rash). While these symptoms overlap those of common infectious illness, the combination of acute onset of both respiratory and gastrointestinal symptoms after starting ABC is more typical of a hypersensitivity reaction. NVP: Systemic symptoms of fever, myalgia, arthralgia, hepatitis, eosinophilia with or without rash.	Discontinue all ARVs until symptoms resolve. The reaction progressively worsens with drug administration and can be fatal. Administer supportive therapy. Do not rechallenge with ABC (or NVP), as anaphylactic reactions and death have been reported. Once symptoms resolve, restart ARVs with change to different NRTI if ABC-associated or to PI- or NRTI-based regimen if NVP-associated.
Severe rash / Stevens-Johnson syndrome	Non nucleoside reverse transcriptase inhibitors (NNRTIs): nevirapine (NVP)	Rash usually occurs during the first 2-4 weeks of treatment. The rash is usually erythematous, maculopapular, confluent, most prominent on the body and arms, may be pruritic and can occur with or without fever. Life-threatening Stevens-Johnson Syndrome or toxic epidermal necrolysis (SJS/TEN) has been reported in ~0.3% of infected individuals receiving NVP	Discontinue all ARVs until symptoms resolve. Permanently discontinue NVP for rash with systemic symptoms such as fever, severe rash with mucosal lesions or urticaria, or SJS/TEN; once resolves, switch ART regimen to different ARV class (e.g., 2 NRTIs and tenofovir or 2 NRTIs and PI).

Adverse Effect	Possible Offending Drug(s)	Clinical Signs / Symptoms	Management
Severe peripheral neuropathy	ddI, d4T, (3TC - unusual)	Pain, tingling, numbness of hands or feet; distal sensory loss, mild muscle weakness, and areflexia can occur.	Stop suspect NRTI and switch to different NRTI that does not have neurotoxicity (e.g., ZDV, ABC). Symptoms usually resolve in 2-3 weeks.
Severe anaemia	ZDV	Severe pallor, tachycardia at rest<br>Shortness of breath on exertion (SOBOE)	Stop ZDV and switch to another drug

Appendix 3.0 Clinical Progression and WHO Definitions

3.1 Paediatric WHO clinical staging for HIV/AIDS for infants and children with established HIV infection

All clinical events or conditions referred to are described in section 0 below.

Clinical Stage 1

Asymptomatic

Persistent generalized lymphadenopathy

Clinical Stage 2

Unexplained persistent hepatosplenomegaly

Papular pruritic eruptions

Extensive wart virus infection

Extensive molluscum contagiosum

Recurrent oral ulcerations

Unexplained persistent parotid enlargement

Lineal gingival erythema

Herpes zoster

Recurrent or chronic respiratory tract infections (otitis media, otorrhoea, sinusitis or tonsillitis)

Fungal nail infections

Clinical Stage 3

Unexplained moderate malnutrition not adequately responding to standard therapy

Unexplained persistent diarrhoea (14 days or more)

Unexplained persistent fever (above 37.5 ^{o}C intermittent or constant, for longer than one month)

Persistent oral candidiasis (after first 6- 8 weeks of life)

Oral hairy leukoplakia

Acute necrotizing ulcerative gingivitis or periodontitis

Lymph node tuberculosis

Pulmonary tuberculosis

Severe recurrent bacterial pneumonia

Symptomatic lymphoid interstitial pneumonitis

Chronic HIV-associated lung disease including brochiectasis

Unexplained anaemia (<8g/dl), neutropenia (<500/mm^3) and/or chronic thrombocytopenia (<50 000/ mm^3)

Clinical Stage 4

Unexplained severe wasting, stunting or severe malnutrition not responding to standard therapy

Pneumocystis pneumonia

Recurrent severe presumed bacterial infections (e.g. empyema, pyomyositis, bone or joint infection, meningitis, but excluding pneumonia)

Chronic herpes simplex infection (orolabial or cutaneous of more than one month's duration or visceral at any site)

Extrapulmonary tuberculosis

Kaposi sarcoma

Oesophageal candidiasis (or candidiasis of trachea, bronchi or lungs)

Central nervous system toxoplasmosis (after one month of life)

HIV encephalopathy

Cytomegalovirus (CMV) infection; retinitis or CMV infection affecting another organ, with onset at age over one month

Extrapulmonary cryptococcosis (including meningitis)

Disseminated endemic mycosis (extrapulmonary histoplasmosis, coccidiomycosis)

Chronic cryptosporidiosis

Chronic isosporiasis

Disseminated non-tuberculous mycobacterial infection

Cerebral or B-cell non-Hodgkin lymphoma

Progressive multifocal leukoencephalopathy

Symptomatic HIV-associated nephropathy or HIV-associated cardiomyopathy

3.2 Presumptive and definitive criteria for recognizing HIV/AIDS-related clinical events in infants and children with established HIV infection

Clinical event	Clinical diagnosis	Definitive diagnosis
**Clinical Stage 1**		
Asymptomatic	No HIV related symptoms reported and no clinical signs on examination.	Not applicable.
Persistent generalized lymphadenopathy	Persistent swollen or enlarged lymph nodes >1 cm at two or more non-contiguous sites (excluding inguinal) without known cause.	Clinical diagnosis
**Clinical Stage 2**		
Unexplained persistent hepatosplenomegaly	Enlarged liver and spleen without obvious cause.	Clinical diagnosis
Papular pruritic eruptions	Papular pruritic vesicular lesions. Also common in uninfected children: scabies and insect bites should be excluded.	Clinical diagnosis
Extensive wart virus infection	Characteristic warty skin lesions; small fleshy grainy bumps, often rough, flat on sole of feet (plantar warts); facial, more than 5% of body area or disfiguring.	Clinical diagnosis
Extensive molluscum contagiosum infection	Characteristic skin lesions: small flesh-coloured, pearly or pink, dome-shaped or umbilicated growths, may be inflamed or red; facial, more than 5% of body area or disfiguring. Giant molloscum may indicate more advanced immunodeficiency	Clinical diagnosis
Fungal nail infections	Fungal paronychia (painful, red and swollen nail bed) or onycholysis (painless separation of the nail from the nail bed.) Proximal white subungual onchomycosis is uncommon without immunodeficiency	Clinical diagnosis
Recurrent oral ulcerations	Current event plus at least one previous episode in past six months. Aphthous ulceration, typically with a halo of inflammation & yellow-grey pseudomembrane.	Clinical diagnosis
Unexplained persistent parotid enlargement	Asymptomatic bilateral swelling that may spontaneously resolve and recur, in absence of other known cause, usually painless	Clinical diagnosis
Lineal gingival erythema	Erythematous band that follows the contour of the free gingival line; may be associated with spontaneous bleeding	Clinical diagnosis

Clinical event	Clinical diagnosis	Definitive diagnosis
Herpes zoster	Painful rash with fluid-filled blisters, dermatomal distribution, can be haemorrhagic on erythematous background, and can become large and confluent. Does not cross the midline	Clinical diagnosis
Recurrent upper respiratory tract infection	Current event with at least one episode in past 6 months. Symptom complex; fever with unilateral face pain and nasal discharge (sinusitis) or painful swollen eardrum (otitis media), sore throat with productive cough (bronchitis), sore throat (pharyngitis) and barking croup-like cough (LTB). Persistent or recurrent ear discharge.	Clinical diagnosis
**Clinical Stage 3**		
[1] Unexplained moderate malnutrition	Weight loss: low weight-for-age, not explained by poor or inadequate feeding and or other infections, and not adequately responding in two weeks to standard management, with downward crossing of at least one complete weight percentile on the CDC 2000 weight-for-age charts	Confirmed by downward crossing of at least one complete weight percentile on the CDC 2000 weight-for-age charts, not responding to standard management and with no other cause identified during investigation.
Unexplained persistent diarrhoea	Unexplained persistent (14 days or more) diarrhoea (loose or watery stool, three or more times daily), not responding to standard treatment.	Confirmed by stools observed and documented as unformed. Culture and microscopy reveal no pathogens.
Unexplained persistent fever (>37.5°C intermittent or constant for longer than one month)	Reports of fever or night sweats for longer than one month, either intermittent or constant, with reported lack of response to antibiotics or antimalarial agents. No other obvious foci of disease reported or found on examination. Malaria must be excluded in malarious areas.	Confirmed by documented fever of >37.5°C with negative blood culture, negative malaria slide and normal or unchanged chest X-ray, and no other obvious foci of disease.
Oral candidiasis (after first 6-8 weeks of life)	Persistent or recurring creamy white to yellow soft small plaques which can be scraped off (pseudomembranous), or red patches on tongue, palate or lining of mouth, usually painful or tender (erythematous form).	Confirmed by microscopy or culture.
Oral hairy leukoplakia	Fine small linear patches on lateral borders of tongue, generally bilaterally, which do not scrape off.	Clinical diagnosis
Lymph node tuberculosis	Non acute, painless "cold" enlargement of peripheral lymph nodes, localized to one region. May have draining sinuses. Response to standard anti- tuberculosis treatment in one month.	Confirmed by histology or fine needle aspirate for Ziehl-Nielsen stain or culture.
Pulmonary tuberculosis	Nonspecific symptoms, e.g. chronic cough, fever, night sweats, anorexia and weight loss. In the older child also productive cough and haemoptysis. History of contact with adults with smear-positive pulmonary tuberculosis. No response to standard broad-spectrum antibiotic treatment.	Confirmed by one or more sputum positive smear for acid-fast bacilli and/or radiographic abnormalities consistent with active tuberculosis and/or culture-positive for *Mycobacterium*.
Severe recurrent bacterial pneumonia	Cough with fast breathing, chest indrawing, nasal flaring, wheezing, and grunting. Crackles or consolidation on auscultation. Responds to course of antibiotics. Current episode plus one or more in previous 6 months.	Confirmed by isolation of bacteria from appropriate clinical specimens (induced sputum, BAL, lung aspirate).
Acute necrotizing ulcerative gingivitis or stomatitis, or acute necrotizing ulcerative periodontitis	Severe pain, ulcerated gingival papillae, loosening of teeth, spontaneous bleeding, bad odour, and rapid loss of bone and/or soft tissue.	Clinical diagnosis
Symptomatic lymphocytic interstitial pneumonitis	No presumptive clinical diagnosis.	Diagnosed by chest X-ray: bilateral reticulonodular interstitial pulmonary infiltrates present for more than two months with no response to antibiotic treatment and no other pathogen found. Oxygen saturation persistently <90%. May present with cor pulmonale and may increased exercise-induced fatigue. Characteristic histology.

Clinical event	Clinical diagnosis	Definitive diagnosis
Chronic HIV-associated lung disease (including bronchiectasis)	History of cough productive of copious amounts of purulent sputum (bronchiectasis only), with or without clubbing, halitosis, and crepitations and/or wheezes on auscultation	Confirmed by chest X-ray: may show honeycomb appearance (small cysts) and/or persistent areas of opacification and/or widespread lung destruction, with fibrosis and loss of volume.
Unexplained anaemia (<8g/dl), or neutropaenia (<0.5 x 10^9 per litre) and/or chronic thrombocytopaenia (<50 x 10^9 per litre)	No presumptive clinical diagnosis.	Diagnosed on laboratory testing, not explained by other non-HIV conditions, not responding to standard therapy with haematinics, antimalarial agents or anthelmintic agents as outlined in WHO Integrated Management of Childhood Illness (IMCI) guidelines.
**Clinical Stage 4**		
[1] Unexplained severe wasting, stunting or severe malnutrition not adequately responding to standard therapy	Persistent weight loss not explained by poor or inadequate feeding, other infections and not adequately responding in two weeks to standard therapy. Characterised by:<br>visible severe wasting of muscles, with or without oedema of both feet, and/or downward crossing of at least two complete weight percentiles on the CDC 2000 weight-for-age charts	Confirmed by downward crossing of at least two complete weight percentiles on the CDC 2000 weight-for-age charts with or without oedema
Pneumocystis pneumonia	Dry cough, progressive difficulty in breathing, cyanosis, tachypnoea and fever; chest indrawing or stridor. (Severe or very severe pneumonia as in WHO IMCI guidelines). Usually rapid onset especially in infants under six months of age. Response to high-dose co-trimoxazole with or without prednisolone. Chest X-ray shows typical bilateral perihilar diffuse infiltrates	Confirmed by: cytology or immunofluorescent microscopy of induced sputum or BAL or histology of lung tissue.
Recurrent severe bacterial infection, e.g. empyema, pyomyositis, bone or joint infection or meningitis but excluding pneumonia	Fever accompanied by specific symptoms or signs that localize infection. Responds to antibiotics. Current episode plus one or more in previous 6 months	Confirmed by culture of appropriate clinical specimen.
Chronic herpes simplex infection; (orolabial or cutaneous of more than one month's duration or visceral at any site)	Severe and progressive painful orolabial, genital, or anorectal lesions caused by HSV infection present for more than one month.	Confirmed by culture and/or histology
Oesophageal candidiasis (or candidiasis of trachea, bronchi or lungs).	Difficulty in swallowing or pain on swallowing (food and fluids). In young children, suspect particularly if oral *Candida* observed and food refusal occurs and/or difficulties or crying when feeding.	Confirmed by macroscopic appearance at endoscopy, microscopy of specimen from tissue or macroscopic appearance at bronchoscopy or histology.
Extrapulmonary or disseminated tuberculosis	Systemic illness usually with prolonged fever, night sweats, and weight loss. Clinical features of organs involved, e.g. sterile pyuria, pericarditis, ascites, pleural effusion, meningitis, arthritis or orchiti, pericardial or abdominal	Confirmed by positive microscopy showing acid-fast bacilli or culture of *Mycobacterium* TB from blood or other relevant specimen except sputum or BAL. Biopsy and histology.
Kaposi sarcoma	Typical appearance in skin or oropharynx of persistent, initially flat, patches with a pink or blood-bruise colour, skin lesions that usually develop into nodules.	Not required but may be confirmed by:<br>- typical red-purple lesions seen on bronchoscopy or endoscopy;<br>- dense masses in lymph nodes, viscera or lungs by palpation or radiology; and<br>- histology.
CMV retinitis or CMV infection affecting another organ, with onset at age over 1 month.	Retinitis only.<br>CMV retinitis may be diagnosed by experienced clinicians: typical eye lesions on serial fundoscopic examination; discrete patches of retinal whitening with distinct borders, spreading centrifugally, often following blood vessels, associated with retinal vasculitis, haemorrhage and necrosis.	Definitive diagnosis required for other sites. Histology. Cerebrospinal fluid polymerase chain reaction

Clinical event	Clinical diagnosis	Definitive diagnosis
CNS toxoplasmosis with onset at age over 1 month.	Fever, headache, focal neurological signs, convulsions. Usually responds within 10 days to specific therapy.	Not required but confirmed by computed tomography (CT) scan (or other neuroimaging) showing single or multiple lesions with mass effect or enhancing with contrast.
Extrapulmonary cryptococcosis (including meningitis)	Meningitis: usually subacute, fever with increasing severe headache, meningism, confusion and behavioural changes that respond to cryptococcal therapy.	Confirmed by CSF microscopy (India ink or Gram stain), serum or CSF CRAG test or culture.
HIV encephalopathy	At least one of the following, progressing over at least two months in the absence of another illness:<br>- failure to attain, or loss of, developmental milestones, loss of intellectual ability; OR<br>- progressive impaired brain growth demonstrated by stagnation of head circumference; OR<br>- acquired symmetric motor deficit accompanied by two or more of the following: paresis, pathological reflexes, ataxia, gait disturbances.	Confirmed by neuroimaging (brain CT scan or MRI) demonstrating atrophy and basal ganglia calcification and excluding other causes.
Disseminated mycosis (coccidiomycosis, histoplasmosis, penicilliosis)	No presumptive clinical diagnosis.	Diagnosed by:<br>Histology: usually granuloma formation.<br>Isolation: antigen detection from affected tissue; culture or microscopy from clinical specimen or blood culture.
Disseminated mycobacteriosis, other than TB	No presumptive clinical diagnosis.	Nonspecific clinical symptoms including progressive weight loss, fever, anaemia, night sweats, fatigue or diarrhoea; plus culture of atypical mycobacterial species from stool, blood, body fluid or other body tissue, excluding lung.
Chronic cryptosporidiosis	No presumptive clinical diagnosis.	Confirmed by cysts identified on modified Ziehl-Nielsen microscopic examination of unformed stool
Chronic *Isospora*	No presumptive clinical diagnosis.	Confirmed in children with chronic diarrhoea by microscopic examination.
Cerebral or B-cell non-Hodgkin lymphoma	No presumptive clinical diagnosis.	Diagnosed by CNS neuroimaging: at least one lesion with mass effect on brain scan; histology of relevant specimen
Progressive multi focal leukoencephalopathy	No presumptive clinical diagnosis.	Diagnosed by progressive nervous system disorder (cognitive dysfunction, gait or speech disorder, visual loss, limb weakness and cranial nerve palsies) together with hypodense white matter lesions on neuroimaging or positive polyomavirus Jacob Creutzfeldt PCR on cerebrospinal fluid
Symptomatic HIV-associated nephropathy	No presumptive clinical diagnosis.	Renal biopsy
Symptomatic HIV-associated cardiomyopathy	No presumptive clinical diagnosis.	Cardiomegaly and evidence of poor left ventricular function confirmed by echocardiography

[1] Moderate (grade 3) and severe (grade 4) malnutrition in children recruited to the trial will be defined using a modified CDC definition [45, 46] by the downward crossing of one or more complete percentiles because all ARROW sites report using the 2000 Centers for Disease Control (CDC) growth charts to record weight.

Appendix 4.0 Handling and Storage of Specimens

4.1 Bloods

4.1.1 *Processing of EDTA blood for plasma and cell store*

For best results, centrifuge within 2 hours of drawing blood. If the time to processing is not within the same working day, **do not proceed**.

1. Centrifuge at 1500g for 15 minutes to separate cells from plasma.

2. Using a sterile pipette, collect the plasma more than 5mm above the buffy cell coat layer (grey layer above red cell layer).

3. Place the plasma, using up to three aliquots, in the cryovials provided for the trial. A minimum of two aliquots is required. Routinely three cryovials of plasma and one cryovial containing the DNA pellet (if feasible) should be stored at each visit.

4. Label the cryovials with the patient "PLASMA" identification labels

5. Store cell pellet for later DNA isolation (if feasible). After plasma has been removed transfer the cell pellet into cryovial

6. Label the cryovials with the patient "CELLS" identification label and store at $^{-}$80°C (if not possible then $^{-}$20°C)

4.1.2 *Storage of samples*

1. Store within 4- 6 hours of drawing blood at -70°C (temperatures to -50°C allowed if this is not available). Place upright in the cryobox provided for the trial.

2. Add the Patient ID label to the Specimen Storage Log for sample. Complete the details for type of specimen processed, visit month, total number of tubes and storage temperature. Start a new log sheet when either the sheet is full or when starting to fill a new cryobox.

4.1.3 *Immunophenotyping*

The Immunophenotyping assay uses two different panels of antibodies, FACS panel I (CD31 AND Ki67) and FACS panel II (CD31 AND HLA-DR). Both panels require 100 µl of heparin or EDTA anti-coagulated whole blood drawn within the last 24 hours. This blood will be taken from existing lymphocyte subset or haematology blood draws. Bloods are mixed with specific buffers and incubated with the correct antibodies and T-cell subsets identified using flow cytometry as described in the ARROW MOP.

4.1.4 *Collection of supernatants using QuantiFERON®-TB Gold In-Tube (IT) system*

The QuantiFERON®-TB Gold In-Tube (IT) system consist of three vacutainer type tubes and 1ml of blood is required for each tube. These 3mls of blood are to be taken from the amount originally allocated for plasma storage.

The three tubes consist of a Nil Control (Grey cap), TB Antigen (Red cap) and Mitogen Control (Purple cap). Antigens have been dried onto the inner wall of the blood collection tubes so it is essential that the contents of the tubes be thoroughly mixed with the blood. The tubes must be transferred to a 37°C incubator as soon as possible and within 16 hours of collection and then incubated and supernatants collected and stored as instructed in the ARROW MOP.

4.1.5 *Storage of Peripheral Blood Mononuclear Cells (PBMCs)*

PBMCs are to be isolated from 3-4mls of the blood that is allocated for plasma storage, ie no extra blood needs to be taken. The cells will be isolated using Ficoll-Paque methods (lab specific) as described in the ARROW MOP.

Appendix 5.0 Management of Safety

(Wording provided by GSK)

5.1 Abacavir Hypersensitivity

In clinical studies, approximately 5% of subjects receiving an abacavir-containing product developed a hypersensitivity reaction, which in rare cases has proved fatal.

5.1.1 *Description of the Hypersensitivity Reaction*

The abacavir hypersensitivity reaction is characterised by the appearance of symptoms indicating multi-organ involvement. The majority of patients have fever and/or rash as part of the syndrome; however reactions have occurred without rash or fever.

Symptoms can occur at any time during treatment with abacavir, but usually appear within the first six weeks of initiation of treatment (median time to onset 11 days). The symptoms worsen with continued therapy and can be life threatening. These symptoms usually resolve shortly after discontinuation of abacavir.

Frequently observed signs and symptoms include fever, rash, malaise or fatigue, gastrointestinal symptoms such as nausea, vomiting, diarrhoea, or abdominal pain and respiratory symptoms such as dyspnoea, sore throat, or cough. Other signs and symptoms include myalgia, arthralgia, oedema, pharyngitis, headache, paresthesia or myolysis.

Physical findings may include rash (usually maculopapular or urticarial), lymphadenopathy or mucous membrane lesions (conjunctivitis, mouth ulceration). Abnormal chest x-ray findings may also be present (predominantly infiltrates, which can be localised).

Laboratory abnormalities may include elevated liver function tests (such as hepatic transaminases), increased creatine phosphokinase or creatinine levels, and lymphopenia.

Anaphylaxis, hypotension, liver failure, renal failure, adult respiratory distress syndrome or respiratory failure may occur.

Some patients with hypersensitivity were initially thought to have respiratory disease (pneumonia, bronchitis, pharyngitis), a flu-like illness, gastroenteritis or reactions to other medications. This delay in diagnosis of hypersensitivity has resulted in abacavir being continued or re-introduced, leading to a more severe hypersensitivity reaction or death. Therefore, the diagnosis of hypersensitivity reaction should be carefully considered for patients presenting with symptoms of these diseases. If hypersensitivity reaction can not be ruled out, no medicinal product containing abacavir (Ziagen, Trizivir or Kivexa) should be restarted.

Patients who develop a hypersensitivity reaction must discontinue abacavir and must never be rechallenged with any medicinal product that contains abacavir (Ziagen, Trizivir or Kivexa). Restarting any abacavir-containing product following a hypersensitivity reaction results in a prompt return of symptoms within hours. This recurrence of the hypersensitivity reaction may be more severe than on initial presentation, and may include life-threatening hypotension and death.

There have been infrequent reports of hypersensitivity reactions following reintroduction of abacavir, where the interruption was preceded by a single key symptom of hypersensitivity (rash, fever, malaise/fatigue, gastrointestinal or a respiratory symptom).

On very rare occasions hypersensitivity reactions have been reported in patients who have re-started therapy, and who had no preceding symptoms of a hypersensitivity reaction.

5.1.2 *Management of Hypersensitivity Reactions*

Patients developing signs or symptoms of hypersensitivity MUST contact their doctor immediately for advice.

If a hypersensitivity reaction is diagnosed the abacavir-containing product MUST be discontinued immediately. The patient should be asked to return all unused supplies of the abacavir-containing product for disposal to prevent an accidental re-challenge.

An abacavir containing medicinal product (Ziagen, Trizivir or Kivexa) MUST NEVER be administered following a hypersensitivity reaction, as more severe symptoms will recur within hours and may include life-threatening hypotension and death.

To avoid a delay in diagnosis and minimise the risk of a life-threatening hypersensitivity reaction, the abacavir-containing product should be permanently discontinued if hypersensitivity cannot be ruled out, even when other diagnoses are possible (respiratory diseases, flu-like illness, gastroenteritis or reactions to other medications).

Symptomatic support for abacavir hypersensitivity may be indicated. This should include, for example, administration of intravenous fluids to patients who develop hypotension. Antihistamines or corticosteroids have been used in cases of abacavir hypersensitivity, however there are no clinical data demonstrating the benefit of these in the management of the reaction.

Laboratory and other investigations which may be useful in the evaluation and treatment of abacavir hypersensitivity include, but may not be limited to, measurement of ALT, AST, creatine phosphokinase, serum creatinine and white blood cell differential count and chest X-ray, if respiratory symptoms are present.

5.1.3 *Special considerations following an interruption of abacavir therapy*

If therapy with abacavir has been discontinued and restarting therapy is under consideration, the reason for discontinuation should be evaluated to ensure that the patient did not have symptoms of a hypersensitivity reaction. **If hypersensitivity reaction cannot be ruled out, no medicinal product containing abacavir (Ziagen, Trizivir or Kivexa) should be restarted.**

There have been infrequent reports of hypersensitivity reaction following reintroduction of an abacavir-containing product where the interruption was preceded by a single key symptom of hypersensitivity (rash, fever, malaise/fatigue, gastrointestinal symptoms or a respiratory symptom). If a decision is made to restart any abacavir-containing product in these patients, this should be done only under direct medical supervision.

On very rare occasions hypersensitivity reactions have been reported in patients who have re-started therapy, and who had no preceding symptoms of a hypersensitivity reaction. If a decision is made to re-start an abacavir-containing product, this must be done only if medical care can be accessed readily by the patient or others.

5.1.4 *Essential patient information*

Investigators must ensure that patients are fully informed regarding the following information on the hypersensitivity reaction (see information sheet for carers Appendix 6.3, page 100):

Patients must be made aware of the possibility of a hypersensitivity reaction to abacavir that may result in a life threatening reaction or death.

Patients developing signs or symptoms possibly linked with a hypersensitivity reaction MUST CONTACT their doctor IMMEDIATELY.

Patients who are hypersensitive to abacavir should be reminded that they must never take any abacavir containing medicinal product (Ziagen, Trizivir or Kivexa) again.

In order to avoid restarting the abacavir-containing product, patients who have experienced a hypersensitivity reaction should be asked to return the remaining tablets or oral solution to the pharmacy.

Patients who have stopped an abacavir-containing product for any reason, and particularly due to possible adverse reactions or illness, must be advised to contact their doctor before restarting.

Each patient should be reminded to read the Package Leaflet included in the pack.

Patients should be reminded of the importance of removing the Alert Card included in the pack, and keeping it with them at all times.

5.1.5 *Reporting of Hypersensitivity Reactions*

All cases of potential abacavir hypersensitivity should be collected and reported to the appropriate Trials Centre on an SAE form regardless of whether or not the event and/or associated signs and symptoms meet the definitions of seriousness (as outlined in section 7.8.1, page 44). In addition to reporting the case as an SAE, the investigator should also complete a'Hypersensitivity Event' CRF and fax this to the MRC CTU within one week of the onset of the hypersensitivity reaction. This will then be forwarded to GlaxoSmithKline.

5.1.6 *Stevens Johnson Syndrome, Toxic Epidermal Necrolysis or Erythema Multiforme*

Serious skin reactions such as Stevens Johnson Syndrome, toxic epidermal necrolysis or erythema multiforme have been reported very rarely in patients taking abacavir-containing products. These patients generally do not have the cluster of additional symptoms (e.g., gastrointestinal and respiratory) that characterize the abacavir hypersensitivity reaction, but they do have features typical of these serious skin reactions.

If a serious skin reaction develops, the abacavir-containing product should be discontinued, and the patient should not be rechallanged with any abacavir-containing medicinal product (Ziagen, Trizivir or Kivexa).

As many products other than abacavir also cause these serious skin reactions, all other medicinal products that the patient is receiving should also be reviewed and discontinued as appropriate.

5.1.7 *Management of Rash that is Not Accompanied by Systemic Symptoms*

Subjects receiving ABC who develop rash of any grade should be evaluated for the possibility of an HSR or a serious skin reaction such as Stevens Johnson Syndrome, toxic epidermal necrolysis or erythema multiforme and managed appropriately as outlined above. Rash may be caused by therapies in any of the major antiretroviral classes, or by other therapies commonly used as concurrent medications, such as cotrimoxazole. As it is not possible to provide an exhaustive list of products that may cause rash in this protocol, please consult the product information leaflets for other products for information relating to rash.

The rash and any associated symptoms should be reported as adverse events (see section 7.8, page 44) and appropriate toxicity ratings should be used to grade the events.

If the aetiology of the rash can be definitively diagnosed as being due to a specific medical event or a concomitant medicinal product, routine management should be performed and documentation of the diagnosis provided.

5.2 Haematological Disorders with zidovudine

Anaemia, neutropenia and leucopenia (usually secondary to neutropenia) can be expected to occur in patients receiving ZDV. Clinicians initiating patients on ZDV-containing regimens in resource-limited settings should be alert to clinical signs of anaemia, and consider closer monitoring of patients with risk factors at screening such as female gender, low body mass index, low CD4 count (and/or high HIV RNA plasma viral load) and/or concurrent bacterial infections or parasitic infestations.

Health care workers need to be vigilant and patients should receive education or counselling about the symptoms of anaemia (see Appendix 0, page 78).

The management of haematological toxicities arising during the conduct of this study should be in accordance with the guidance for management of toxicity given earlier in the protocol (section 8.1.4, page 48)

5.3 Nucleoside Reverse Transcriptase Inhibitors, including abacavir, lamivudine and zidovudine

5.3.1 *Lactic Acidosis/Severe Hepatomegaly with Steatosis*

The relevance of asymptomatic lactic acid elevations is unclear, and lactates are not part of the routine safety evaluations or monitoring for this study. Should an investigator suspect the occurrence of lactic acidosis in any subject, the MRC CTU should be consulted. Lactic acidosis and severe hepatomegaly with steatosis, including fatal cases, have been reported with the use of antiretroviral nucleoside analogues alone or in combination in the treatment of HIV infection. A majority of these cases have been in adult women. This syndrome is felt to be associated with mitochondrial toxicity induced by the inhibitory effect of NRTIs on deoxyribonucleic acid (DNA) polymerase gamma, a key enzyme needed for mitochondrial DNA synthesis. Current knowledge regarding this syndrome is incomplete. Obesity and prolonged NRTI exposure may be risk factors.

Symptoms of lactic acidosis frequently involve non-specific symptoms such as fatigue, weakness, and fever, but in the majority of cases also involve symptoms suggestive of hepatic dysfunction such as nausea, vomiting, abdominal or epigastric discomfort, abdominal distension, hepatomegaly, and new onset elevated liver enzymes. Caution should be exercised when administering abacavir, lamivudine, zidovudine, Trizivir, Kivexa or Combivir to any subject and particularly to those with known risk factors for liver disease. Treatment with abacavir, lamivudine, zidovudine, Trizivir, Kivexa or Combivir should be suspended in any subject who develops clinical or laboratory findings suggestive of lactic acidosis or hepatotoxicity.

5.3.2 *Pancreatitis*

Cases of pancreatitis have occurred rarely in subjects treated with abacavir, lamivudine and zidovudine. However it is not clear whether these cases were due to the medicinal products or to the underlying HIV disease. Treatment with abacavir, lamivudine, zidovudine, Trizivir,

Kivexa or Combivir should be stopped immediately if clinical signs, symptoms or laboratory abnormalities suggestive of pancreatitis occur.

5.3.3 *Immune Reconstitution Syndrome*

In HIV-infected patients with severe immune deficiency at the time of initiation of anti-retroviral therapy (ART), an inflammatory reaction to asymptomatic or residual opportunistic infections may arise and cause serious clinical conditions, or aggravation of symptoms. Typically, such reactions have been observed within the first few weeks or months of initiation of ART. Relevant examples are cytomegalovirus retinitis, generalised and/or focal mycobacterial infections and *Pneumocystis jiroveci* (*P. carinii*) pneumonia. Any inflammatory symptoms must be evaluated without delay and treatment initiated when necessary.

When these syndromes are suspected the following management plan should be followed:

- Continue antiretroviral treatment.
- Confirm diagnosis of opportunistic infection (OI).
- Continue or initiate specific therapy for the infection.
- Evaluate the participant clinically to exclude a new infectious process if the participant was already receiving therapy for an OI.
- Initiate anti-inflammatory agents, initially non-steroidals or, if needed corticosteroids at the discretion of the site investigator in consultation with the protocol team and only if hypersensitivity to abacavir can be ruled out.

Appendix 6.0 Patient Information Sheets and Consent Forms (Templates)

6.1 Information sheet for carers

[Each country to use its own translated Informed Consent according to local regulatory requirements, on local headed paper for each site]

ARROW (AntiRetroviral Research fOr Watoto)

Information for <u>parents and carers</u>

Introduction
In this leaflet, we refer to 'your child' to mean the child for whom you are considering participation in this study. You and your child are being invited to take part in a research study (ARROW) being carried out in Uganda and Zimbabwe. Before you decide it is important for you to understand why the research is being done and what it will involve. Please take time to read the following information carefully or have someone read it to you, and discuss it with others if you wish. You will be given a copy to keep. Ask the clinic nurses or counsellors if there is anything that is not clear or if you would like more information. Joining the ARROW study is entirely voluntary. Take time to decide whether or not you wish your child to take part.

If either you or your child is already registered with a health system group (e.g. DART, OI clinic, TASO in Uganda or Parirenyatwa National Hospital in Zimbabwe), joining this study will not affect your rights to any benefits it offers. You may decide that your child should not take part now or you may wish to withdraw him or her from the study later. This will not influence the care he or she receives now or in future. Neither would your decision on behalf of your child affect the care you receive yourself. We hope, however, that you would give a reason if you wished to withdraw your child and would continue to allow us to provide follow-up care which involves continued regular medical checkups even if your child is no longer taking the study medicines.

What is the reason for doing the ARROW study?
HIV is the virus that causes AIDS. At the moment, there is no cure for HIV. However, there are anti-HIV medicines, called 'anti-retrovirals' that can help to control it. We want to find out how best to use these anti-HIV medicines in children in Africa so that treating them is safe and effective. The medicines are given daily and currently doctors use regular blood tests to see how well the medicines are working and to check for any side-effects, even when patients appear to be benefiting from the treatment. The ARROW study is investigating two questions:

1) **Whether the medicines can be taken without doing so many blood tests.**
2) **Whether adding an extra medicine to 3 standard medicines for a short period of time when your child starts therapy is more effective over the long term.**

What will happen if my child takes part?

Your child will be started on anti-HIV medicines and will be put into one of two groups (chosen by chance by a computer) to determine how the doctor will look after them:

- **clinically driven monitoring (CDM) group**

  OR

- **laboratory and clinical monitoring (LCM) group**

Blood tests will be done whenever your child sees the doctor. In the **CDM group** the doctor will make decisions about your child's treatment based only on your child's symptoms. The doctor may use these symptoms to ask to see the blood test results looking for drug side effects but they will not routinely get these, unless there is a serious side-effect in which case results will automatically be returned.

In the LCM group your doctor will make decisions about your child's treatment based on his or her symptoms and the results of regular blood tests looking for side-effects and how your child's immune system (which fights infections) is responding to HIV. It is important to know that none of the children in ARROW will receive tests measuring the amount of HIV in their blood.

In both groups, you will be asked to bring your child to attend clinics to see both doctor and nurse at two and four weeks after entry to the study. Your child will then attend the clinic every four weeks to see the nurse, and every twelve weeks to see the doctor and to provide a blood sample. At each visit you, the carer, will be given a supply of anti-HIV medicines and asked to follow exact instructions on how your child should be given these medicines.

It is very important that you keep the ARROW study medicines in a safe place, **preferably locked away, out of the reach of children.**

As parent or carer you will be fully informed at all stages and will be invited to attend all clinic visits with your child. You will decide with the doctor what information is appropriate to give to your child. You will be asked to make sure that your child takes or is given the ARROW study medicines according to instructions and will be given advice about this.

How many medicines will my child take?

When your child enters the study he or she will be selected (again by chance, chosen by a computer) to receive one of three different combinations of anti-retroviral therapy:

1) treatment daily with 3 anti-HIV medicines (nevirapine or efavirenz (depending on age and availability – both medicines are from the same drug group), lamivudine and abacavir) for the duration of the study

   OR

2) treatment daily with 4 anti-HIV medicines (nevirapine or efavirenz, lamivudine, abacavir and zidovudine) for 36 weeks then dropping zidovudine and taking the other 3 medicines for the rest of the study

   OR

3) treatment daily with 4 anti-HIV medicines (nevirapine or efavirenz, lamivudine, abacavir and zidovudine) for 36 weeks then dropping nevirapine/efavirenz and taking the other 3 medicines for the rest of the study.

Whichever group they are in, your child will continue to take co-trimoxazole (septrin) to help to protect against infections.

What are the possible risks and benefits of clinically driven monitoring and laboratory and clinical monitoring?

The aim of giving anti-HIV medicines is to keep your child healthy for as long as possible using the limited number of medicines and healthcare resources (doctors, healthcare centres and laboratories) available. In rich countries, doctors do blood tests for side-effects and to

measure how much the medicines decrease the virus in the blood and increase the immune cells (CD4 count) which have been damaged by HIV; these tests are usually done every 12 weeks. However, these tests are often unavailable in Africa and are costly. ARROW is asking whether these tests need to be done to keep children healthy in Africa, or whether it is as safe and effective to do tests for side-effects only when a doctor thinks they are needed.

The potential risks if your child is in the group where test results are not seen by your doctor unless they show severe-effects is that he/she could have more severe side-effects if the doctor is looking only at clinical symptoms. Other risks are that your child may stay on the same first-line anti-HIV medicines longer, even after they are not working so well, because the doctor is not seeing CD4 counts. This could result in the HIV developing more resistance to the medicines. However, it is important to remember that staying on first-line anti-HIV medicines for longer could be just as good or even better for your child's health in the long term because options for treatment after first-line are likely to be more limited.

What are the possible risks and benefits of the different drug treatments?

All the medicines we are using in ARROW include abacavir, lamivudine and either nevirapine or efavirenz, a three medicine combination recommended by the World Health Organisation (WHO) guidelines. However, studies in the West have generally shown that children do not respond as well to anti-HIV medicine as adults – reasons may be that it is harder to persuade children to take their medicines, that children depend on caregivers to give medicines, and that HIV may be stronger because the immune system in a child is not as mature as it is in an adult.

Small studies in the West have shown that children taking four medicines (not three) have a very strong response to HIV. In ARROW we want to see if taking an extra fourth medicine for the first 36 weeks of treatment (induction phase) helps to increase the length of time the medicines will work well. The risks are that your child could have side-effects to this extra medicine, or that you could find giving four medicines more difficult than three. The potential benefits are that the immune system may respond better both initially and over the long term if a fourth medicine is used in the first 36 weeks.

For children starting four medicines, ARROW is looking at two possibilities after the first 36 weeks. One is to stop zidovudine and continue on a standard three medicine combination. The other is to stop nevirapine or efavirenz and continue taking three medicines which are all from the same group (NRTIs).

Studies in the West have shown that around 10% fewer adults have no HIV found in their blood if this three NRTI treatment is used. However, there are possible advantages: when a child fails first-line anti-HIV medicines they have two medicine groups which are likely to be active against HIV, and so may have a better second-line group of medicines to take. In addition, three NRTI medicines can be taken with anti-TB treatment if your child should need this, whereas nevirapine cannot be taken and would need to be changed.

It is important that you understand that your child is in a research study and that this particular study is designed to find out what is the best way to give first-line anti-HIV medicines. You may therefore find out in years to come, when you look back, that your child may or may not have received the best treatment. As a research study the medicines being used, although all recommended by WHO, are not all the same as in the national programmes.

How is it related to the DART study?

The CDM and LCM part of ARROW is similar to the DART study in adults. The reason for studying this question in children is that the answers may be different compared to adults, because children's immune systems (the way they fight infections like HIV) behave differently. The way children's growth responds to anti-HIV medicines may also provide extra information about how the medicines are working.

How long will the study continue?
It is planned that each child will be in the study for between 3½ and 5 years and will be provided with anti-HIV medicines for that time. After the end of the study, care will be provided for your child by the national health system including provision of anti-HIV medicines in line with current national programmes.

How important is it to take the syrup or tablets regularly?
It is very important that

- your child does not miss any doses of the medicines
- the medicines are not shared with anyone else

If anti-HIV medicines are not taken as the doctor tells you they may lose their effect, as the HIV virus can become resistant to them. Other medicines should only be taken on the advice of your doctor. If your child does not attend a clinic visit, we would like to send a home visitor to your home to find out how your child is.

Blood samples
After starting anti-HIV first or second-line medicines in ARROW, blood samples will be taken at weeks 4, 8, and 12. Thereafter blood tests will be done whenever your child sees the doctor (every 3 months). Taking blood may cause some discomfort and/or bruising in some children. About two teaspoons of blood will be taken at one time. Some blood may be stored for later testing to find out exactly how your child's immune system was fighting HIV, and you may not be given these results. New tests are being developed all the time, so we are asking for you to agree that blood samples from your child can be used in the future for any tests that are relevant to HIV, either during the ARROW study or after it ends. These tests would be decided by a group of HIV experts.

What medicines will be used?
The doctor will give you a card explaining which medicines your child will take. We know that it can be difficult giving anti-HIV medicines to your child every day. We want to know how well children take their medicines and if there are any particular problems taking them. To do this, at intervals during the study we will ask you some questions about how you and your family manage the taking of these medicines. Children from the age of 12 will be asked to fill out their own questionnaire.

Are there any side effects?
Anti-HIV medicines, like all medicines, have side effects, which are sometimes (less than 1 in 20 cases) serious. The most common side effects of drugs used in ARROW are rash (nevirapine/efavirenz), anaemia (zidovudine) and allergic reactions (abacavir, please see also separate sheet). If your child is unwell, and you are worried about them, you should contact the clinic as soon as possible. If it is not serious then tell the clinic doctor at your child's next visit. It may be necessary to stop one or more of the medicines, after which the problem usually goes away. We can replace medicines causing problems with other medicines.

What about sexual heath advice and pregnancy?
Since the study includes boys and girls up to the age of 18, older children will be counselled about issues related to sexual health. Those who are sexually active will be counselled appropriately and girls will be counselled to use effective contraception. HIV treatment given early in pregnancy may harm the unborn child so pregnant and breast-feeding girls will not be entered into the ARROW study. For girls of appropriate age, a pregnancy test will be

carried out before entry to the study and at regular intervals during the study and girls will be encouraged to avoid becoming pregnant during the study. If they do become pregnant, they will continue to receive anti-HIV medicines and be followed up in the study, and will be closely monitored.

What happens to the information collected in the ARROW study?
ARROW clinic staff will collect information whenever they see your child. This information will be stored in a computer and an independent committee, the Data Monitoring Committee, will look at it regularly. They will inform the investigators if one treatment group looks better than the others, so that children could all be moved over to that group. Information from the ARROW study will be analysed and the results will be presented and published to improve the future care of children with HIV in Africa.

Confidentiality
Information about your child will be kept confidential and will not be made available to anyone who is not connected with the ARROW study without your, and if appropriate your child's, consent. In particular, your child's medical notes and study information will be available to study staff and may also be seen by other independent people authorised to ensure that the ARROW study is being properly carried out. Strict confidentiality will be maintained at all times. Names will not be used for study information and stored blood samples; these will be identified only by a study number, date of birth and initials. There will be one list which links this study number to your child's name, and this list will be safely kept private in a locked cabinet.

How can my child join the ARROW study?
After reading this information sheet you will be asked to give consent for your child to be seen by the doctor and to give a blood sample. After two weeks, the blood test results will come back to the clinic and you will be told whether your child is eligible to start anti-HIV therapy in ARROW. If they are, you will be asked to sign a form giving consent for your child to participate. A home visitor will come to your home with you at the time of enrolment so that during the study they can help remind you about clinic appointments, and make sure that you and your child are alright if you are not able to attend the clinic as planned.

If you would like more information or have any questions about the ARROW study please ask the doctors, nurses or counsellors. If you still need more information, please call:

Insert names and telephone numbers as appropriate:

Name:

Telephone Number: ……………………………………………….

If your child has an injury or illness during his/her participation in ARROW, please contact:

Dr…………………………………on………………………………

Nurse……………………………on………………………

6.2 Information sheet for children

[Each country to use its own translated Informed Consent according to local regulatory requirements, on local headed paper for each site]

ARROW (AntiRetroviral Research fOr Watoto) Study

Information for <u>children and young people</u>

Introduction

In this leaflet, your 'carer' means the person who takes care of you, this could be your parent, a family member or another adult. You are being invited to take part in a research study called ARROW, being carried out in Uganda and Zimbabwe. Please read this information carefully or have someone read it to you, and discuss it with the people who look after you. You can ask us if there is anything you don't understand or are worried about and we will be happy to give you more information. You will be given a copy of this information to keep.

If you or your carer decide you should not take part in this study you can be sure that this will not affect the medical care you will get in future. The doctors and nurses will not mind if you don't take part. If you join the study but change your mind later on, you can stop taking part and it will not affect the care you get in the future either.

What is the reason for doing the ARROW study?

HIV is the virus that causes AIDS. At the moment, there is no cure for HIV. However, there are anti-HIV medicines, called 'anti-retrovirals' that can help to control it. We want to find out how best to use these anti-HIV medicines in children and young people in Africa so that treating them is safe and effective. There is already a similar study (DART) being carried out in adults. Currently medicines are given daily and doctors use regular blood tests to see how well the medicines are working and to check for any side effects, even when patients appear to be benefiting from the treatment. The ARROW study is investigating two questions:

1) **Whether the medicines can be taken without doing so many blood tests.**
2) **Whether adding an extra medicine to the 3 standard medicines for a short period of time when you start therapy is more effective over the long term.**

What will happen when I take part?

Your carer will be invited to come with you to the clinic whenever possible. The doctor or nurse will make sure that both of you are kept informed about your participation in the study and you can ask questions at any time.

You will be started on anti-HIV medicines and will be put into one of two groups (decided by chance by a computer) to check how you are responding to your treatment. These groups are:

Clinically driven monitoring (CDM)	***Laboratory and clinical monitoring (LCM)***
Your doctor will decide on your care by seeing how you are and asking about any medical problems (the doctor can ask for the results of your blood tests if he or she needs them to assist with your care)	Your doctor will decide on your care by seeing how you are and asking about any medical problems AND by using the results of blood tests looking for side-effects and at how your immune system is responding to HIV

Whichever group you are in:

- you will come to the clinic every 4 weeks to see the nurse and either a doctor or counsellor. You will be asked to give a blood sample every three months.
- at these clinic visits you will be given a supply of anti-HIV medicines for you to take. You and your carer will be told exactly how and when you should take them.

You will be asked to make sure you take your medicines according to the instructions. This is very important if the medicines are to continue to work. Your carer will be asked to assist you.

How many medicines will I take?

When you enter the study you will be selected (again by chance, chosen by a computer) to receive one of three different combinations of anti-HIV medicines. The doctor will give you a card to explain which anti-HIV medicines you will be taking, and you will continue to take co-trimoxazole (septrin) to help protect you from getting infections.

How is it related to the adult DART study?

The DART study in adults is similar to ARROW. The reason for doing ARROW as well is that the answers we get may be different between children and young people and adults because the way children and young people fight infections like HIV is different to adults. Also children will have to take medicines for longer whilst growing and going through puberty.

How long will the study continue?

You will be in the study for between 3½ and 5 years. If you miss your clinic appointments we would like to send someone to your home to find out how you are.

How important is it to take the medicines regularly?

It is very important that

- you do not miss any doses
- the medicines are not shared with anyone else.

If anti-HIV medicines are not taken as the doctor tells you, they can sometimes lose their effect in fighting the virus.

We know that it can be difficult taking anti-HIV medicines every day. We want to know how well you take the medicines and if there are any particular problems with taking them. To do this, at intervals during the study we will ask you some questions about how you and your family manage the taking of these medicines.

Giving blood samples

Blood tests will be done when you see the doctor. This may cause some discomfort or cause a bruise at the place from where the blood is taken (usually from your arm). Some blood may be stored for later testing to find out exactly how the medicines might be helping you to fight HIV, and you may not be given these results.

What about side effects?

Anti-HIV medicines, like all medicines, can have side effects, which are sometimes serious. If you are unwell you should contact the clinic as soon as possible. If not serious then tell the clinic nurse at your next visit.

Confidentiality

Information about you will be kept confidential and will not be made available without your consent to anyone who is not connected with the ARROW study. Names will not be used for study information and stored blood samples; these will be identified only by a study number, date of birth and initials.

How can I join the ARROW study?

If you and your carer decide to take part, you will visit the doctor who will talk to you, examine you and take a small amount of blood for testing. After two weeks, the test results will come back to the clinic and you will be told whether you can start anti-HIV medicines in the study. If you then agree to join the study, your carer will be asked to sign a form to agree that you can take part.

If you would like more information, or have any questions, about the ARROW study please ask the doctors, nurses or counsellors. If you still need more information, call:

* Insert names and telephone numbers as appropriate

Name: ……………………………………………………………..

Telephone Number: ……………………………………………….

6.3 Information about abacavir for carers

About 5 in every 100 people who are treated with abacavir may develop a hypersensitivity reaction (serious allergic reaction). This reaction can sometimes be life threatening and in rare cases has been fatal.

Although hypersensitivity reaction (serious allergic reaction) to abacavir usually appears within the first six weeks of abacavir treatment, symptoms can occur at any time while being treated with abacavir. You will have been given a warning card from your child's abacavir medication to remind you about abacavir hypersensitivity and these symptoms. **You should keep this card with your child at all times**.

Most, but not all, patients have fever (high temperature) and/or rash as part of the reaction. Some of the other symptoms may include tiredness (or generally ill feeling) nausea, vomiting, diarrhoea, or abdominal pain, difficulty breathing, sore throat or cough. Other signs and symptoms may include muscle and joint aches and pains, swelling, numbness or tingling, headaches and anaphylaxis (a severe form of allergic reaction). Occasionally inflammation of the eye (conjunctivitis), ulcers in the mouth or low blood pressure may occur. Symptoms generally get worse with continued treatment.

If your child develops symptoms that may be suggestive of abacavir hypersensitivity, or if your child stops taking their study medication for any reason, and particularly due to possible side effects described above, you should contact the clinic as soon as possible and follow the detailed guidance printed on the warning card from your child's abacavir medication.

If your child becomes hypersensitive to abacavir, which will be ultimately decided in consultation with your child's study doctor, then you should return all of his/her unused abacavir (ZIAGEN) to the study site for proper disposal.

6.4 Screening Consent Form

Fill in with Forms 0 & 1	ARROW TRIAL<br>SCREENING CONSENT

This form should be completed before the screening visit for children whose carers are considering entry into the ARROW Trial. A patient information sheet should be provided before this form.

Centre: Entebbe ○ Harare ○ JCRC ○ Mulago ○

Child's Initials				Male ○<br>Female ○	Date/Year of Birth	D	D	M	M	M	Y	Y	Y	Y	Age (years)		

Date of Form	D	D	M	M	M	2	0	Y	Y	Clinic/Hospital Number								

ARROW TRIAL SCREENING CONSENT

Please initial (or mark) box if you agree:

**1)**	I/my child have read/been read the information sheet for the ARROW study and agree to my child being assessed for eligibility for this study.	
**2)**	I/my child understand this will involve my child being seen by the doctor, who will ask questions about my child's health and our household, and my child having some blood taken for testing, including an HIV test.	
**3)**	I/my child agree that the results of these tests, and any blood left over from them, may be kept by the ARROW team for further studies on HIV. I understand that these results and samples will not be identified by either my or my child's name.	

Carer's signature (or thumbprint)	Print name	Date
		D D M M M 2 0 Y Y
Child's signature (or thumbprint) *where appropriate*	Print name	Date
		D D M M M 2 0 Y Y
Witness's signature (if thumbprint used above)	Print name	Date
		D D M M M 2 0 Y Y
Doctor's signature	Print name	Date
		D D M M M 2 0 Y Y

IMPORTANT: one signed original to be given to carer, one signed original to be kept on file by the researcher, one signed original to be kept in the clinic notes.

6.5 Trial Consent Form

Fill in with Forms 2 & 4	ARROW TRIAL<br>TRIAL CONSENT

Centre: Entebbe ○ Harare ○ JCRC ○ Mulago ○

Child's Initials	Male ○<br>Female ○	Date/Year of Birth D D M M M Y Y Y Y	Age (years)
Randomisation/ Enrolment Date* D D M M M 2 0 Y Y	Clinic/Hospital Number		
Date of Form D D M M M 2 0 Y Y	ARROW Trial Number*		

*add after randomisation

ARROW TRIAL CONSENT for PARENTS/GUARDIANS and CHILDREN

Please initial (or mark) box if you agree:

1. I/My child have read/been read the information sheet for the ARROW study and any questions we had, have been satisfactorily answered. I/my child understand what is required if s/he participates in ARROW.
2. I/My child understand that s/he will be given anti-HIV drugs for 4-5 years while they are in the ARROW study. After the study, my child's healthcare will be provided by the national health system.
3. I/My child understand that I/my child may withdraw from the ARROW study at any time, without giving a reason and without affecting their normal care and management.
4. I/My child agree to allow blood samples to be taken from my child, and stored with anonymised labelling for testing later. I/my child understand we may not be given results of tests performed on stored samples.
5. I/My child are willing to allow access to her/his medical notes to check that the ARROW study is being carried out correctly and we understand that strict confidentiality will be maintained.
6. I/My child agree that s/he may take part in the ARROW study
7. I understand that if I am unable to continue to be the main carer for my child that I need to provide the clinic with the name of the person who will become the main carer so their consent can be requested.

ADHERENCE SUBSTUDY (optional):
I/My child agree to take part in the adherence substudy and to being visited at home as part of this substudy.

Signature	Print name	Date
Carer's signature (or thumbprint)		D D M M M 2 0 Y Y
Child's signature (or thumbprint) *where appropriate*		D D M M M 2 0 Y Y
Witness's signature (if thumbprint used above)		D D M M M 2 0 Y Y
Doctor's signature		D D M M M 2 0 Y Y

IMPORTANT: one signed original to be given to carer, one signed original to be kept on file by the researcher, one signed original to be kept in the clinic notes

6.6 Assent Form

ARROW TRIAL

TRIAL ASSENT

Centre: Entebbe ○ Harare ○ JCRC ○ Mulago ○

Child's Initials		Male ○ Female ○		Date/Year of Birth	D D M M M Y Y Y Y	Age (years)	
Randomisation/ Enrolment Date*	D D M M M 2 0 Y Y			Clinic/Hospital Number			
Date of Form	D D M M M 2 0 Y Y			ARROW Trial Number*			

ARROW TRIAL ASSENT for CHILDREN 13 TO 17 YEARS OLD

Please initial (or mark) box if you agree:

I have read and understood / been read to and understand the information sheet for the ARROW study.	
Any questions I had have been answered.	
I understand that my participation is voluntary and agree to continue participating in the ARROW study.	

Child's signature (or thumbprint) where appropriate	Print name	Date
		D D M M M 2 0 Y Y

Doctor's signature	Print name	Date
		D D M M M 2 0 Y Y

IMPORTANT: one signed original to be kept in ARROW trial file by the researcher,
one signed copy to be given to carer, one signed copy to be kept in the clinic notes.

Page 1 of 1 – v1.0, 18 April 2008 ISRCTN24791884

6.7 Stop or Continue cotrimoxazole randomisation

6.7.1 Information sheet for carers

[Each country to use its own translated Informed Consent according to local regulatory requirements, on local headed paper for each site]

ARROW (AntiRetroviral Research fOr Watoto)

STOP OR CONTINUE COTRIMOXAZOLE

Information for parents and carers

Introduction

In this leaflet, we refer to 'your child' to mean the child already taking part in the ARROW study. Here we are inviting you and your child to additionally join an extra part of the ARROW study. This is because we know that giving anti-retrovirals or ARVs to your child every day is difficult for long periods of time, so we want to look at ways to make taking ARVs long-term easier.

Please take time to read the following information carefully or have someone read it to you, and discuss it with others if you wish. You will be given a copy to keep. Ask the clinic nurses or counsellors if there is anything that is not clear or if you would like more information. Take time to decide whether or not you wish your child to take part. Joining or not joining this new ARROW substudy will not change how your child is treated in ARROW.

What is the reason for looking at stopping cotrimoxazole in the ARROW study?

Before your child started ARVs they would also have been given a common antibiotic, called cotrimoxazole (also called septrin), to take every day. HIV made it harder for your child to fight bugs, so this antibiotic helped to do this for them. Now your child has been on ARVs for over 18 months, you will have noticed that they are doing much better than before – and might wonder whether they still need to take cotrimoxazole with their ARVs, particularly as it is an extra pill every day. We want to find out whether taking cotrimoxazole with ARVs is worth it – does it actually still stop children getting sick when they've taken ARVs for over 18 months?

What will happen if my child takes part?

Your child will be put into one of two groups (chosen by chance by a computer)

- **stop daily cotrimoxazole and just take ARVs**

  OR

- **continue taking daily cotrimoxazole with ARVs**

Nothing else will change – your child will come to clinic just as before, will have the same blood tests, will still take the same ARVs and will continue to be managed by the doctor in the same way as before depending on whether they are in the LCM or CDM group.

What are the possible risks and benefits of stopping cotrimoxazole?

The possible risk of stopping cotrimoxazole is that your child will not be able to fight bugs as well as if they continued to take it, even though they are still on ARVs. In particular, cotrimoxazole can stop your child getting malaria. If you stop cotrimoxazole and you live somewhere where malaria is common, it is very important to make sure your child sleeps

under an insecticide treated bednet. If your child has a fever, contact the ARROW clinic immediately.

The possible benefit of stopping cotrimoxazole is that your child won't have to take so many drugs every day. This may make it easier to regularly take the ARV drugs which are essential to keep the HIV virus under control.

How long will the study continue?
The study will continue until the end of ARROW (end of March 2012). After the end of the study, care will be provided for your child by the national health system including provision of anti-HIV medicines in line with current national programmes.

What happens to the information collected in the ARROW study?
ARROW clinic staff will collect information whenever they see your child whether you join this extra study or not. This information will be stored in a computer and an independent committee, the Data Monitoring Committee, will look at it regularly. They will inform the investigators if the stop or continue cotrimoxazole group looks better, so that children could all be moved over to that group. Information from the ARROW study will be analysed and the results will be presented and published to improve the future care of children with HIV in Africa.

Confidentiality
Information about your child will be kept confidential and will not be made available to anyone who is not connected with the ARROW study without your, and if appropriate your child's, consent. In particular, your child's medical notes and study information will be available to study staff and may also be seen by other independent people authorised to ensure that the ARROW study is being properly carried out. Strict confidentiality will be maintained at all times. Names will not be used for study information and stored blood samples; these will be identified only by a study number, date of birth and initials. There will be one list which links this study number to your child's name, and this list will be safely kept private in a locked cabinet.

How can my child join the STOP or CONTINUE COTRIMOXAZOLE substudy?
After reading this information sheet you will be asked to sign a form giving consent for your child to participate the next time you see the doctor.

If you would like more information or have any questions about this study please ask the doctors, nurses or counsellors. If you still need more information, please call:

Insert names and telephone numbers as appropriate:

Name:
Telephone Number: ……………………………………………….

If your child has an injury or illness during his/her participation in ARROW, please contact:

Dr…………………………………on………………………………

Nurse……………………………on………………………

6.7.2 Information sheet for children

[Each country to use its own translated Informed Consent according to local regulatory requirements, on local headed paper for each site]

ARROW (AntiRetroviral Research fOr Watoto) Study

STOP OR CONTINUE COTRIMOXAZOLE

Information for children and young people

Introduction

In this leaflet, your 'carer' means the person who takes care of you, this could be your parent, a family member or another adult. Here we are inviting you to additionally join an extra part of the ARROW study. This is because we know that taking anti-retrovirals or ARVs every day is difficult for long periods of time, so we want to look at ways to make taking ARVs long-term easier.

Please read this information carefully or have someone read it to you, and discuss it with the people who look after you. You can ask us if there is anything you don't understand or are worried about and we will be happy to give you more information. You will be given a copy of this information to keep. The doctors and nurses will not mind if you don't take part. If you join this extra study but change your mind later on, you can stop taking part and it will not affect the care you get in ARROW in the future either.

What is the reason for looking at stopping cotrimoxazole in the ARROW study?

Before you started ARVs you may also have been given a common antibiotic, called cotrimoxazole (also called septrin), to take every day. HIV made it harder for you to fight bugs, so this antibiotic helped to do this for you. Now that you have been on ARVs for over 18 months, you will have noticed that you are doing much better than before – and might wonder whether you still need to take cotrimoxazole with your ARVs, particularly as it is an extra pill every day. We want to find out whether taking cotrimoxazole with ARVs is worth it – does it actually still stop you getting sick when you've taken ARVs for over 18 months?

What will happen if I take part?

You will be put into one of two groups (chosen by chance by a computer)

- **stop daily cotrimoxazole and just take ARVs**

  OR

- **continue taking daily cotrimoxazole with ARVs**

Nothing else will change – you will come to clinic just as before, will have the same blood tests, will still take the same ARVs and will continue to be managed by the doctor in the same way as before depending on whether you are in the LCM or CDM group.

What are the possible risks and benefits of stopping cotrimoxazole?

The possible risk of stopping cotrimoxazole is that you won't be able to fight bugs as well as if you continued to take it, even though you are on ARVs. In particular, cotrimoxazole can stop you getting malaria. If you stop cotrimoxazole and you live somewhere where malaria is common, it is very important to make sure you sleep under an insecticide treated bednet. If you has a fever, contact the ARROW clinic immediately.

The possible benefit of stopping cotrimoxazole is that you won't have to take so many drugs every day. This may make it easier for you to regularly take the ARV drugs which are essential to keep the HIV virus under control.

How long will the study continue?
The study will continue until the end of ARROW (end of March 2012).

Confidentiality
Information about you will be kept confidential and will not be made available without your consent to anyone who is not connected with the ARROW study. Names will not be used for study information and stored blood samples; these will be identified only by a study number, date of birth and initials.

How can I join the STOP or CONTINUE COTRIMOXAZOLE substudy?
After reading this information sheet you will be asked to sign a form giving consent to participate the next time you see the doctor.

If you would like more information, or have any questions, about the ARROW study please ask the doctors, nurses or counsellors. If you still need more information, call:

* Insert names and telephone numbers as appropriate

Name: ……………………………………………………………..

Telephone Number: ……………………………………………….

6.7.3 Stop or Continue Cotrimoxazole Consent Form

ARROW TRIAL

COTRIMOXAZOLE RANDOMISATION CONSENT

Centre: Entebbe ○ Harare ○ JCRC ○ Mulago ○

Child's Initials				Male ○ Female ○	Date/Year of Birth	D	D	M	M	M	Y	Y	Y	Y	Age (years)		

Randomisation Date	D	D	M	M	M	2	0	Y	Y	Clinic/Hospital Number								
Date of Form	D	D	M	M	M	2	0	Y	Y	ARROW Trial Number								

ARROW STOP OR CONTINUE COTRIMOXAZOLE RANDOMISATION CONSENT for PARENTS/CARERS and CHILDREN

Please initial (or mark) box if you agree:

I/my child have read/been read the information sheet for the Stop or Continue Cotrimoxazole Randomisation in ARROW, and any questions we had have been satisfactorily answered.	
I/my child understand that I/my child may withdraw from the ARROW study at any time, without giving a reason and without affecting their normal care and management.	
I/my child agree that s/he may take part in the Stop or Continue Cotrimoxazole Randomisation in ARROW	
I understand if I am unable to continue to be the main carer for my child that I need to provide the clinic with the name of the person who will become the main carer so their consent for the Stop or Continue Cotrimoxazole Randomisation in ARROW can be requested	

Carer's signature (or thumbprint)	Print name	Date
		D D M M M 2 0 Y Y
Child's signature (or thumbprint) *where appropriate*	Print name	Date
		D D M M M 2 0 Y Y
Witness's signature (if thumbprint used above)	Print name	Date
		D D M M M 2 0 Y Y
Doctor's signature	Print name	Date
		D D M M M 2 0 Y Y

IMPORTANT: one signed original to be kept in ARROW trial file by the researcher, one signed copy to be given to carer, one signed copy to be kept in the clinic notes.

6.7.4 Stop or Continue Cotrimoxazole Assent Form

ARROW TRIAL
COTRIMOXAZOLE RANDOMISATION ASSENT

Centre: Entebbe ○ Harare ○ JCRC ○ Mulago ○

Child's Initials		Male ○ Female ○	
Date/Year of Birth	D D M M M Y Y Y Y	Age (years)	
Randomisation/ Enrolment Date*	D D M M M 2 0 Y Y	Clinic/Hospital Number	
Date of Form	D D M M M 2 0 Y Y	ARROW Trial Number*	

ARROW STOP OR CONTINUE COTRIMOXAZOLE ASSENT for CHILDREN 13 TO 17 YEARS OLD

Please initial (or mark) box if you agree:

I have read and understood / been read to and understand the information sheet for the Stop or Continue Cotrimoxazole Randomisation in ARROW	
Any questions I had have been answered.	
I understand that my participation is voluntary and agree to continue participating in the randomisation to Stop or Continue Cotrimoxazole in ARROW	

Child's signature (or thumbprint) *where appropriate*	Print name	Date
		D D M M M 2 0 Y Y

Doctor's signature	Print name	Date
		D D M M M 2 0 Y Y

IMPORTANT: one signed original to be kept in ARROW trial file by the researcher, one signed copy to be given to carer, one signed copy to be kept in the clinic notes.

6.8 Once versus twice daily lamivudine+abacavir randomisation

6.8.1 Information sheet for carers

[Each country to use its own translated Informed Consent according to local regulatory requirements, on local headed paper for each site]

ARROW (AntiRetroviral Research fOr Watoto)

ONCE or TWICE DAILY 3TC+ABC

Information for parents and carers

Introduction

In this leaflet, we refer to 'your child' to mean the child already taking part in the ARROW study. Here we are inviting you and your child to additionally join an extra part of the ARROW study. This is because we know that giving anti-retrovirals or ARVs to your child every day is difficult for long periods of time, so we want to look at ways to make taking ARVs long-term easier.

Please take time to read the following information carefully or have someone read it to you, and discuss it with others if you wish. You will be given a copy to keep. Ask the clinic nurses or counsellors if there is anything that is not clear or if you would like more information. Take time to decide whether or not you wish your child to take part. Joining or not joining this new ARROW substudy will not change how your child is treated in ARROW.

What is the reason for looking at once and twice daily 3TC+ABC in the ARROW study?

Adults can take two of the ARVs your child is taking, 3TC (lamivudine) and ABC (abacavir) either once or twice a day and both are just as good at keeping the HIV virus under control. But no large studies have been done in children. What we know is that the overall levels of the drugs in the blood are similar in African children taking 3TC and ABC once or twice a day. Now we want to make sure that children are just as able to fight the HIV virus if they take these drugs once or twice a day.

What will happen if my child takes part?

Your child will be put into one of two groups (chosen by chance by a computer)

- **change to taking 3TC and ABC once a day (either in the morning or evening, whichever is more convenient)**

  OR

- **continue taking 3TC and ABC twice a day**

Nothing else will change – your child will come to clinic just as before, will have the same blood tests, will still take their other ARVs in the same way and will continue to be managed by the doctor in the same way as before depending on whether they are in the LCM or CDM group.

What are the possible risks and benefits of moving to once daily 3TC+ABC?

The possible risk of moving to once daily 3TC+ABC is that your child will not be able to control the HIV virus as well as if they were taking these drugs twice a day. One reason why this might happen is because if your child misses one dose when they are taking 3TC+ABC twice a day they still get some drug – whereas if they miss one dose when they are taking 3TC+ABC once a day they miss these drugs for the whole day.

The possible benefit of moving to once daily 3TC+ABC is that you and your child may find it easier to give no or fewer drugs in the morning or evening. If the other drugs your child takes can also be taken once a day, you could just give your child drugs in the morning or evening. Even if they still take some drugs twice a day, it may still be easier to only have to think about some of the drugs once a day, particularly if the child is spending time with different carers. Moving to once daily 3TC+ABC may make it easier to regularly take the ARV drugs which are essential to keep the HIV virus under control. Once it is decided whether your child will be on morning or evening dosing, they should not switch between the two but rather try to stick to **either** mornings only **or** evenings only as far as possible.

How long will the study continue?
The study will continue until the end of ARROW (end of March 2012). After the end of the study, care will be provided for your child by the national health system including provision of anti-HIV medicines in line with current national programmes.

What happens to the information collected in the ARROW study?
ARROW clinic staff will collect information whenever they see your child whether you join this extra study or not. This information will be stored in a computer and an independent committee, the Data Monitoring Committee, will look at it regularly. They will inform the investigators if the stop or continue cotrimoxazole group looks better, so that children could all be moved over to that group. Information from the ARROW study will be analysed and the results will be presented and published to improve the future care of children with HIV in Africa.

Confidentiality
Information about your child will be kept confidential and will not be made available to anyone who is not connected with the ARROW study without your, and if appropriate your child's, consent. In particular, your child's medical notes and study information will be available to study staff and may also be seen by other independent people authorised to ensure that the ARROW study is being properly carried out. Strict confidentiality will be maintained at all times. Names will not be used for study information and stored blood samples; these will be identified only by a study number, date of birth and initials. There will be one list which links this study number to your child's name, and this list will be safely kept private in a locked cabinet.

How can my child join the ONCE or TWICE daily 3TC+ABC substudy?
After reading this information sheet you will be asked to sign a form giving consent for your child to participate the next time you see the doctor.

If you would like more information or have any questions about this study please ask the doctors, nurses or counsellors. If you still need more information, please call:

Insert names and telephone numbers as appropriate:

Name:

Telephone Number: ……………………………………………….

If your child has an injury or illness during his/her participation in ARROW, please contact:
Dr……………………………….on……………………….………
Nurse……………………………on……………………….

6.8.2 Information sheet for children

[Each country to use its own translated Informed Consent according to local regulatory requirements, on local headed paper for each site]

ARROW (AntiRetroviral Research fOr Watoto) Study

ONCE OR TWICE DAILY 3TC+ABC

Information for <u>children and young people</u>

Introduction

In this leaflet, your 'carer' means the person who takes care of you, this could be your parent, a family member or another adult. Here we are inviting you to additionally join an extra part of the ARROW study. This is because we know that taking anti-retrovirals or ARVs every day is difficult for long periods of time, so we want to look at ways to make taking ARVs long-term easier.

Please read this information carefully or have someone read it to you, and discuss it with the people who look after you. You can ask us if there is anything you don't understand or are worried about and we will be happy to give you more information. You will be given a copy of this information to keep. The doctors and nurses will not mind if you don't take part. If you join this extra study but change your mind later on, you can stop taking part and it will not affect the care you get in ARROW in the future either.

What is the reason for looking at once and twice daily 3TC+ABC in the ARROW study?

Adults can take two of the ARVs you are taking, 3TC (lamivudine) and ABC (abacavir) either once or twice a day and both are just as good at keeping the HIV virus under control. But no large studies have been done in children or adolescents. What we know is that the overall levels of the drugs in the blood are similar in African children taking 3TC and ABC once or twice a day. Now we want to make sure that children and adolescents are just as able to fight the HIV virus if they take these drugs once or twice a day.

What will happen if I take part?

You will be put into one of two groups (chosen by chance by a computer)

- **change to taking 3TC and ABC once a day (either in the morning or evening, whichever is more convenient)**

  OR

- **continue taking 3TC and ABC twice a day**

Nothing else will change – you will come to clinic just as before, will have the same blood tests, will still take their other ARVs in the same way and will continue to be managed by the doctor in the same way as before depending on whether they are in the LCM or CDM group.

What are the possible risks and benefits of moving to once daily 3TC+ABC?

The possible risk of moving to once daily 3TC+ABC is that you will not be able to control the HIV virus as well as if you were taking these drugs twice a day. One reason why this might happen is because if you miss one dose when you are taking 3TC+ABC twice a day you will still get some drug – whereas if you miss one dose when you are taking 3TC+ABC once a day you will miss these drugs for the whole day.

The possible benefit of moving to once daily 3TC+ABC is that you may find it easier to take no or fewer drugs in the morning or evening. If the other drugs you take can also be taken

once a day, you could just take your ARVs in the morning or evening. Even if you still take some drugs twice a day, it may still be easier to only have to think about some of the drugs once a day, particularly if you spend time with different carers. Moving to once daily 3TC+ABC may make it easier to regularly take the ARV drugs which are essential to keep the HIV virus under control. Once it is decided which dosing you will be on, you should not switch between the two but rather try to stick to **either** mornings only **or** evenings only as far as possible.

How long will the study continue?
The study will continue until the end of ARROW (end of March 2012).

How can I join the ONCE or TWICE daily 3TC+ABC substudy?
After reading this information sheet you will be asked to sign a form giving consent to participate the next time you see the doctor.

If you would like more information, or have any questions, about the ARROW study please ask the doctors, nurses or counsellors. If you still need more information, call:

* Insert names and telephone numbers as appropriate

Name: …………………………………………………………………..

Telephone Number: ……………………………………………….

6.8.3 Once Daily Versus Twice Daily Randomisation Consent Form

ARROW TRIAL

ONCE DAILY VERSUS TWICE DAILY RANDOMISATION CONSENT

Centre: Entebbe ○ Harare ○ JCRC ○ Mulago ○

Child's Initials				Male ○ Female ○	Date/Year of Birth	D	D	M	M	M	Y	Y	Y	Y	Age (years)		

Randomisation Date	D	D	M	M	M	2	0	Y	Y	Clinic/Hospital Number								
Date of Form	D	D	M	M	M	2	0	Y	Y	ARROW Trial Number								

ARROW ONCE DAILY VERSUS TWICE DAILY RANDOMISATION CONSENT for PARENTS/CARERS and CHILDREN

Please initial (or mark) box if you agree:

I/my child have read/been read the information sheet for the Once Daily Versus Twice Daily Randomisation in ARROW, and any questions we had have been satisfactorily answered.	
I/my child understand that I/my child may withdraw from the ARROW study at any time, without giving a reason and without affecting their normal care and management	
I/my child agree that s/he may take part in the Once Daily Versus Twice Daily Randomisation in ARROW	
I understand if I am unable to continue to be the main carer for my child that I need to provide the clinic with the name of the person who will become the main carer so their consent for the Once Daily Versus Twice Daily Randomisation in ARROW can be requested	

Carer's signature (or thumbprint)	Print name	Date
		D D M M M 2 0 Y Y
Child's signature (or thumbprint) *where appropriate*	Print name	Date
		D D M M M 2 0 Y Y
Witness's signature (if thumbprint used above)	Print name	Date
		D D M M M 2 0 Y Y
Doctor's signature	Print name	Date
		D D M M M 2 0 Y Y

IMPORTANT: one signed original to be kept in ARROW trial file by the researcher, one signed copy to be given to carer, one signed copy to be kept in the clinic notes.

6.8.4 Once Daily Versus Twice Daily Randomisation Assent Form

ARROW TRIAL

ONCE DAILY VERSUS TWICE DAILY RANDOMISATION ASSENT

Centre: Entebbe ○ Harare ○ JCRC ○ Mulago ○

Child's Initials				Male ○ Female ○	Date/Year of Birth	D	D	M	M	M	Y	Y	Y	Y	Age (years)		

Randomisation Date	D	D	M	M	M	2	0	Y	Y	Clinic/Hospital Number								
Date of Form	D	D	M	M	M	2	0	Y	Y	ARROW Trial Number								

ARROW ONCE DAILY VERSUS TWICE DAILY COTRIMOXAZOLE ASSENT for CHILDREN 13 TO 17 YEARS OLD

Please initial (or mark) box if you agree:

I have read and understood / been read to and understand the information sheet for the Once Daily Versus Twice Daily Randomisation in ARROW	
Any questions I had have been answered.	
I understand that my participation is voluntary and agree to continue participating in the Once Daily Versus Twice Daily Randomisation in ARROW	

Child's signature (or thumbprint) *where appropriate*	Print name	Date
		D D M M M 2 0 Y Y

Doctor's signature	Print name	Date
		D D M M M 2 0 Y Y

IMPORTANT: one signed original to be kept in ARROW trial file by the researcher, one signed copy to be given to carer, one signed copy to be kept in the clinic notes.

Appendix 7.0 Adherence Questionnaires

7.1 Adherence questionnaire for carers

FORM 14a	ARROW TRIAL<br>**ADHERENCE – FOR CARERS**

Centre: Entebbe ○ Harare ○ JCRC ○ Mulago○

Child's Initials		Male ○<br>Female ○	Date/Year of Birth	D D M M M Y Y Y Y	Age (years)	

Week Number (If unscheduled tick extra)		Extra ☐	Clinic/Hospital Number	

Date of Form	D D M M M 2 0 Y Y	ARROW Trial Number		Arm of Study	CDM ○<br>LCM ○

To the nurse/counsellor (or doctor): Please go through the questionnaire with the carer by reading out the questions to them and explaining anything that they do not understand. Remind them that this questionnaire is only about the antiretroviral medicines that they are giving to the child.

1. What is your relationship to the child? (please tick one)

Mother .... ☐ Father .... ☐ Brother .... ☐ Sister .... ☐
Aunt .... ☐ Uncle .... ☐ Cousin .... ☐ Grandmother (maternal) . ☐
Grandmother (paternal) ... ☐ Grandfather (maternal) ... ☐ Grandfather (paternal) ..... ☐ Friend .... ☐

Other (specify) .... ☐______________________

2. Who gives antiretroviral medicines to the child? (please tick all that apply)

Mother .... ☐ Father .... ☐ Brother .... ☐ Sister .... ☐
Aunt .... ☐ Uncle .... ☐ Cousin .... ☐ Grandmother (maternal) . ☐
Grandmother (paternal) ... ☐ Grandfather (maternal) ... ☐ Grandfather (paternal) ..... ☐ Friend .... ☐

Other (specify) .... ☐______________________

3. Now I am going to review with you how your doctor/pharmacist has instructed you to give antiretroviral medicines to the child – thinking ONLY about the medicines you received at the last visit

	Name of Medicine(s) (carer may describe based on colour, size etc. If they don't know name; enter either medicine name or "don't know")	How many times a day should the medicine be given? (enter "don't know" if appropriate)	For pills/tablets only: Enter how many pills should be given in the morning		For pills/tablets only: Enter how many pills should be given in the evening	
1.			☐ ½ pills	☐ whole pills	☐ ½ pills	☐ whole pills
2.			☐ ½ pills	☐ whole pills	☐ ½ pills	☐ whole pills
3.			☐ ½ pills	☐ whole pills	☐ ½ pills	☐ whole pills
4.			☐ ½ pills	☐ whole pills	☐ ½ pills	☐ whole pills

4. How many times has the child missed a dose of their antiretroviral medicines over the last 3 days?
If no doses missed over the last 3 days tick here ☐

Medicine name	Yesterday	Day Before Yesterday	3 days ago
1.	..... dose(s) missed	..... dose(s) missed	..... dose(s) missed
2.	..... dose(s) missed	..... dose(s) missed	..... dose(s) missed
3.	..... dose(s) missed	..... dose(s) missed	..... dose(s) missed
4.	..... dose(s) missed	..... dose(s) missed	..... dose(s) missed

5. How much does giving antiretroviral medicines to the child interfere with YOUR everyday life? (tick one box):
A lot ☐ quite a lot ☐ not much ☐ not at all ☐
How? (please specify) ______________________

6. How much does giving antiretroviral medicines to your child interfere with THEIR everyday life? (tick one box):
A lot ☐ quite a lot ☐ not much ☐ not at all ☐
How? (please specify) ______________________

7. What reminds you to give the antiretroviral medicines? (tick all that apply)

Labels ☐ Timer/alarm clock/beeper ☐
Daily events (e.g. breakfast time) ☐ Support from the child/other family member ☐
Help from doctors/nurses at the clinic ☐ Knowing why my child needs to take medicines ☐
Other ☐ *Specify:* ______________________

Page 1 of 2 – v1.1, 27 March 2008 ISRCTN24791884

c8. If the child has missed any antiretroviral doses since last scheduled doctor visit, please tick the reason(s):
If no doses missed during last 12 weeks tick here ☐

Is it because:	Please tick one box per reason		
	Never	Sometimes	Often
You had run out of medicine?	☐	☐	☐
The child had problems taking the medicine? (e.g. hard to swallow or bad taste) Specify: ______	☐	☐	☐
The medicine made the child unwell?	☐	☐	☐
You had forgotten or were too busy?	☐	☐	☐
You think the medicines were harmful?	☐	☐	☐
Taking the medicine was difficult with school hours, meals, sleep etc?	☐	☐	☐
The child refused to take them?	☐	☐	☐
The child was being looked after by someone else?	☐	☐	☐
You did not want other people to know the child was taking medicine?	☐	☐	☐
The child was unwell?	☐	☐	☐
The medicine made the child feel hungry?	☐	☐	☐
Your routine, or the child's routine, was different from normal? (e.g. holidays, weekends etc)?	☐	☐	☐
You were too depressed?	☐	☐	☐
You were too unwell?	☐	☐	☐
You were fed up giving medicines?	☐	☐	☐
You wanted to give the child a break from the medicine?	☐	☐	☐
You did not want to give too much medicine?	☐	☐	☐

Further details or any other reason (please name them): ______

______

9. Is the child taking divided tablets? .... Yes ☐ No ☐
   (a) If yes, have you had any problems snapping them? .... Yes ☐ No ☐
   If yes, what problems have you had? (tick all that apply):
   Crumbling ☐ Uneven parts ☐ Difficult to snap by hand ☐ Other ☐ ______
   (b) If yes, have you had any problems remembering how many half or whole tablets should be given?
   .... Yes ☐ No ☐
   (c) If yes, have you lost a half tablet from a previous dose?.................................... Yes ☐ No ☐

10. If taking tablets:
   (a) How are you giving the tablets to your child?
   Dissolved in water.. Often ☐ Sometimes ☐ Never ☐
   Crumbled/mixed with food.. Often ☐ Sometimes ☐ Never ☐
   Whole/broken but not with anything else........................ Often ☐ Sometimes ☐ Never ☐
   (b) Does your child generally manage to swallow the tablets straight away or after several attempts?
   Swallows straight away? .... Often ☐ Sometimes ☐ Never ☐
   Swallows on second attempt? .... Often ☐ Sometimes ☐ Never ☐
   Swallows after several attempts? .... Often ☐ Sometimes ☐ Never ☐
   (c) If on whole tablets: since the last visit has your child choked on one of these tablets? Yes ☐ No ☐
   (d) If on half-tablets: since the last visit has your child choked on one of these tablets? Yes ☐ No ☐
   (e) Have you had any difficulty transporting tablets during your daily activities away from home?
   .... Yes ☐ No ☐

11. If taking syrups:
   (a) Have you had any problems with them? .... Yes ☐ No ☐
   If yes, what problems have you had? (tick all that apply):
   Too many bottles ☐ Too bulky to store ☐ Difficult to measure ☐ Difficult to keep cool ☐
   Too heavy to carry ☐ Too conspicuous ☐ Other (specify) ☐ ______
   (b) Have you had any difficulty transporting syrups during your daily activities away from home?
   .... Yes ☐ No ☐

12. Does the child know his or her infection status?... Yes ☐ No ☐

Thank you for taking the time to fill out this form; please add any comments you have:

______

______

______

Clinic Nurse or Counsellor's Signature	Print name	Date
		D D M M M 2 0 Y Y

Page 2 of 2 – v1.1, 27 March 2008 ISRCTN24791884

7.2 Adherence questionnaire for children/adolescents

FORM 14b	ARROW TRIAL<br>**ADHERENCE – FOR ADOLESCENTS**

Centre: Entebbe ○ Harare ○ JCRC ○ Mulago○

Child's Initials	☐☐☐	Male ○<br>Female ○	Date/Year of Birth	D D M M M Y Y Y Y	Age (years)	☐☐

Week Number (If unscheduled tick extra)	☐☐☐	Extra ☐	Clinic/Hospital Number	☐☐☐☐☐☐☐☐

Date of Form	D D M M M 2 0 Y Y	ARROW Trial Number	☐☐☐☐☐☐☐	Arm of Study	CDM ○<br>LCM ○

To the nurse/counsellor (or doctor): Please go through the questionnaire with the child or teenager by reading out the questions to them and explain anything that they do not understand. Remind them that this questionnaire is only about the antiretroviral medicines that they are taking.

1. First I am going to review with you how your doctor/pharmacist has instructed you to take the antiretroviral medicines – thinking ONLY about the medicines you received at the last visit

	Name of Medicine(s) (child/teenager may describe based on colour, size etc. If they don't know name; enter either medicine name or "don't know")	How many times a day should you take the medicine? (enter "don't know" if appropriate)	For pills/tablets only:<br>Enter how many pills should be taken in the morning		For pills/tablets only:<br>Enter how many pills should be taken in the evening	
1.			☐ ½ pills	☐ whole pills	☐ ½ pills	☐ whole pills
2.			☐ ½ pills	☐ whole pills	☐ ½ pills	☐ whole pills
3.			☐ ½ pills	☐ whole pills	☐ ½ pills	☐ whole pills
4.			☐ ½ pills	☐ whole pills	☐ ½ pills	☐ whole pills

2. How many times have you missed a dose of your medicines over the last 3 days?
If no doses missed over the last 3 days tick here ☐

Medicine Name	Yesterday	Day Before Yesterday	3 days ago
1.	..... dose(s) missed	..... dose(s) missed	..... dose(s) missed
2.	..... dose(s) missed	..... dose(s) missed	..... dose(s) missed
3.	..... dose(s) missed	..... dose(s) missed	..... dose(s) missed
4.	..... dose(s) missed	..... dose(s) missed	..... dose(s) missed

3. How much does taking the medicines interfere with your everyday life? (tick one box)
A lot ☐ quite a lot ☐ not much ☐ not at all ☐
How? (please specify)__________________________________

4. What reminds you to take the medicines? (tick all that apply)
Labels ☐ Timer/alarm clock/beeper ☐
Daily events (e.g. breakfast time) ☐ Support from Mum/Dad/Carer ☐
Help from doctors/nurses at the clinic ☐ Knowing why I need to take medicines ☐
Other ☐ *Specify:*______________________________

5. Does anyone remind you when to take your medicine? .... Yes ☐ No ☐
If yes, please tick all the people who remind you:
Mother .... ☐ Father .... ☐ Brother .... ☐ Sister .... ☐
Aunt .... ☐ Uncle .... ☐ Cousin .... ☐ Grandmother (maternal) . ☐
Grandmother (paternal) ... ☐ Grandfather (maternal) .... ☐ Grandfather (paternal) .... ☐ Friend .... ☐
Other (specify) .... ☐ ____________________

6. If you have missed any doses since the last scheduled doctor visit, please tick the reason(s):
If no doses missed over the last 12 weeks tick here ☐

Is it because:	Please tick one box per reason		
	No	Sometimes	Often
You had run out of medicine?	☐	☐	☐
You had problems taking the medicine? (e.g. can't swallow them or bad taste). Specify: ______	☐	☐	☐
The medicine made you feel unwell?	☐	☐	☐
You had forgotten or were too busy?	☐	☐	☐
You think the medicines were harmful?	☐	☐	☐
Taking the medicine is difficult with school hours, meals, sleep etc?	☐	☐	☐
You didn't want to take them?	☐	☐	☐
You were being looked after by someone else?	☐	☐	☐
You did not want other people to know you were taking medicine?	☐	☐	☐
You felt unwell?	☐	☐	☐
The medicine made you feel hungry?	☐	☐	☐
Your routine was different from normal (e.g. holidays, weekends etc)?	☐	☐	☐
You were too depressed?	☐	☐	☐
You were fed up taking medicine?	☐	☐	☐
You wanted a break from the medicine?	☐	☐	☐
You did not want to take too much medicine?	☐	☐	☐

Further details or any other reason (please name them): ______

______

______

7. Are you taking divided tablets? .... Yes ☐ No ☐
(a) If yes, have you had any problems snapping them? .... Yes ☐ No ☐
If yes, what problems have you had? (tick all that apply):
Crumbling ☐ Uneven parts ☐ Difficult to snap by hand ☐ Other ☐ ______
(b) If yes, have you had any problems remembering how many half or whole tablets to take?
.... Yes ☐ No ☐
(c) If yes, have you lost a half tablet from a previous dose?.................................... Yes ☐ No ☐

8. If taking tablets:
(a) How do you take them?
Dissolved in water.. Often ☐ Sometimes ☐ Never ☐
Crumbled/mixed with food.. Often ☐ Sometimes ☐ Never ☐
Whole/broken but not with anything else.................................. Often ☐ Sometimes ☐ Never ☐
(b) Do you generally manage to swallow the tablets straight away or after several attempts?
Swallows straight away? .... Often ☐ Sometimes ☐ Never ☐
Swallows on second attempt? .... Often ☐ Sometimes ☐ Never ☐
Swallows after several attempts? .... Often ☐ Sometimes ☐ Never ☐
(c) If on whole tablets: since the last visit have you choked on one of these tablets? Yes ☐ No ☐
(d) If on half-tablets: since the last visit have you choked on one of these tablets?...... Yes ☐ No ☐
(e) Have you had any difficulty transporting tablets during your daily activities away from home?
.... Yes ☐ No ☐

Thank you for taking the time to fill out this form; please add any comments you have:

______

______

______

Clinic Nurse or Counsellor's Signature	Print name	Date
		D D M M M 2 0 Y Y

Page 2 of 2 – v1.1 27 March 2008 ISRCTN24791884

Appendix 8.0 Trial consent withdrawal form

Fill in when a patient withdraws partial/full consent for the ARROW trial	ARROW TRIAL<br>**CONSENT WITHDRAWAL FORM**

Centre: Entebbe ○ Harare ○ JCRC ○ Mulago ○

Child's Initials				Male ○ Female ○	Date/Year of Birth	D D M M M Y Y Y Y	Age (years)		

Randomisation/ Enrolment Date	D D M M M 2 0 Y Y	Clinic/Hospital Number	

Date of Form	D D M M M 2 0 Y Y	ARROW Trial Number		Arm of Study	CDM ○ LCM ○

ARROW TRIAL CONSENT WITHDRAWAL for CARERS and CHILDREN

Please initial (or mark) box if you agree:

I/my child no longer wish to (or cannot) take trial anti-HIV drugs and do not wish to (or cannot) attend further visits. I/my child agree to being contacted in the future (home visits or telephone) and to my/my child's medical records being consulted in future to obtain clinical information for ARROW.	

Set in place procedure to follow the child up through visits and medical records and report any trial outcomes on the appropriate form. Inform the child and carer that s/he may still return for follow-up visits only or for trial drugs and follow-up visits at a later date.

I/my child no longer wish to (or cannot) take trial anti-HIV drugs, do not wish to (or cannot) attend further visits and do not want to be contacted in the future. I/my child agree to my/my child's medical records being consulted in future to obtain clinical information for ARROW.	

Set in place procedure to follow the child up through medical records alone and report any trial outcomes on the appropriate form. Inform the child and carer that s/he may still return for follow-up visits only or for trial drugs and follow-up visits at a later date.

I/my child no longer wish to (or cannot) take trial anti-HIV drugs and do not wish to (or cannot) attend further visits. I/my child do not agree to being contacted in the future or to my/my child's medical records being consulted in future to obtain clinical information for ARROW.	

Discontinue all follow up through medical records. The child and carer must sign a new consent form if s/he decides to rejoin the study at a later date.

Carer's signature (or thumbprint)	Print name	Date
		D D M M M 2 0 Y Y
Child's signature (or thumbprint) *where appropriate*	Print name	Date
		D D M M M 2 0 Y Y
Witness's signature (if thumbprint used above)	Print name	Date
		D D M M M 2 0 Y Y
Doctor's signature	Print name	Date
		D D M M M 2 0 Y Y

IMPORTANT: One signed original to be given to patient
One signed original to be kept on file by the researcher
One signed original to be kept in the clinic notes

Page 1 of 1 v1.0, 19 December 2006 ISRCTN24791884
